# Development of Lipopeptides as Orthoflavivirin Inhibitors with Low Micromolar Broad-Spectrum Antiorthoflaviviral Activity

**DOI:** 10.1021/acs.jmedchem.5c01364

**Published:** 2025-10-16

**Authors:** Lorenzo Cavina, Anna Alocén Portillo, Mike P. A. Balmer, Jenny C. Dammer, Danae Schillemans, Said Hakim Hamdani, Bart Ackerschott, Cindy E. J. Dieteren, Byron E. E. Martina, Bernd N. M. van Buuren, Alexandra Rockstroh, Sebastian Ulbert, Pedro H. H. Hermkens, Montse Llinàs Brunet, Daniel Gironés, Martin C. Feiters, Floris P. J. T. Rutjes

**Affiliations:** † Institute for Molecules and Materials, 6029 Radboud University, Heyendaalseweg 135, 6525 AJ Nijmegen, The Netherlands; ‡ Protinhi Therapeutics, Transistorweg 5, 6534 AT Nijmegen, The Netherlands; § Department of Infection Research and Diagnostics, 28433 Fraunhofer Institute for Cell Therapy and Immunology, Perlickstr. 1, 04103 Leipzig, Germany; ∥ HermkensPharmaConsultancy B.V., Gripper 1, 5348 KZ Oss, The Netherlands

## Abstract

Orthoflaviviral infections increasingly impact the global population; no specific therapeutic treatments are available. The orthoflaviviral protease NS2B-NS3 is a promising target for antiviral drug development. Here, we present the design, synthesis, structure–activity relationship (SAR), and in vivo PK study of a novel lipopeptide scaffold emerging from exploration of the previously investigated polycationic geminoids **4–6**. The *N*-palmitoyl moiety is essential for protease inhibition; optimization of the peptide sequence led to lipopeptides **73** and **79**, which selectively inhibited dengue virus (DENV2) NS2B-NS3 and exhibited low micromolar antiviral potency in DENV2-, West Nile virus (WNV)-, and Zika virus (ZIKV)-infected cells without significant cytotoxicity. Compound **73** (Palmitoyl-Lys-Ala-d-Ala-Lys-NH_2_) demonstrated a favorable in vivo pharmacokinetic profile in BALB/c mice following intravenous (IV), intraperitoneal (IP), and subcutaneous (SC) administration, showing stability and good tolerability. Herein, we detail the SAR of the lipopeptide scaffold and suggest its potential for in vivo therapeutic application administered 20 mg/kg subcutaneously *b.i.d.*.

## Graphical abstract

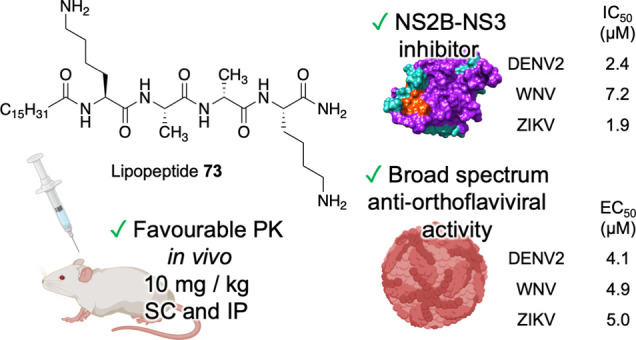

## Introduction

*Orthoflavivirus* is a *genus* of viruses from the family of *Flaviviridae* in which the virions are mostly characterized by thick protein envelopes which encapsulate a 9–13 kilobases positive-sense, single-stranded and nonsegmented RNA.^1,2^ Although viral transmission vectors and tissue tropism differ greatly between *Flaviviridae*, they share many structural similarities.^1,3^ Mammals are typical hosts for orthoflaviviruses, and those that are pathogenic to humans are commonly arthropod-borne, such as dengue virus (DENV, *O. denguei*), yellow fever virus (YFV, *O. flavi*), West Nile virus (WNV, *O. nilense*), Japanese encephalitis virus (JEV, *O. japonicum*), and Zika virus (ZIKV, *O. zikaense*). Orthoflaviviruses are vectored by mosquitoes of the *Aedes* and *Culex genera* and some species of ticks.^2–5^ The vectors are most commonly found in tropical and subtropical urban and semiurban areas of the world, but infections are also rapidly spreading through the northern hemisphere^6,7^ due to a plethora of factors, such as the adaptation of the vectors to colder climates,^8^ the trend of increasing median temperatures owing to global warming,^9^ and increased human travel because of globalization.^10^ DENV, the etiologic agent of dengue fever, is considered the most common representative of its *genus*, being endemic to areas of the world harboring in total half of the human population.^11^ Dengue fever generally causes severe joint pain, which is highly incapacitating, forcing the patient in an immobility status throughout the course of the infection. ZIKV has recently gained attention in the wake of the 2015–2016 pandemic, which highlighted the unexpectedly severe pathologies deriving from orthoflaviviral infections.^12^ Although these infections are seldom lethal, they can result in severe pathological outcomes, including microcephaly in unborned children and Guillain-Barré syndrome due to ZIKV infections,^13,14^ and poliomyelitis-like symptoms due to WNV infections,^15,16^ dengue hemorrhagic fever and shock syndrome due to DENV infections.^17^ To date, other than palliative care, there are no approved treatments for such morbidities, while vaccination clinical trials have very recently been prompted for ZIKV, WNV and other orthoflaviviruses.^18^ YFV has regained clinical attention after recent outbreaks^19^ but was not included in this study because a safe single-dose vaccine provides lifelong protection.^20^ Dengvaxia is the main vaccine available against DENV, but it has been approved only for people who had already been previously infected.^21,22^ Recent vaccination campaigns for DENV have shown dubious efficacy and larger risks than are acceptable for such a prophylactic therapy,^22^ thereby emphasizing once more the global need for a specific antiviral treatment. Upon infection by orthoflaviviruses, the viral RNA is translated by host machinery into a single viral polyprotein. Processing of the latter by one viral and several host proteases results in the release of three structural proteins and seven nonstructural proteins.^23^ The viral protease is a nonstructural protein known as NS3 containing an N-terminal serine protease domain joined to an RNA helicase domain by an 11-amino acid linker.^24^ The protease catalytic activity is mediated by the amino acid triad Ser135, His51, and Asp75. After self-cleavage from the viral polyprotein, NS3 forms a heterodimer with NS2B in order to fully exert its proteolytic activity on the viral polyprotein. The active orthoflaviviral protease, called orthoflavivirin and henceforth referred to as NS2B-NS3, cleaves the viral polyprotein at specific sites characterized by a sequence of cationic amino acids P1–P4,^25^ which are recognized by the specificity pockets S1–S4 near the active site of the protease.^26^ NS2B-NS3 is essential for viral maturation and has therefore emerged as a potential target in antiorthoflaviviral drug development.^27^ Exploiting the structure of the natural substrate of the viral protease as a scaffold for the development of antivirals is an effective strategy which has already been successfully applied in the development of marketed antivirals against HIV,^28^ HCV^29,30^ and SARS-CoV-2.^31^ Given the structural and functional similarities among orthoflaviviruses,^32^ we foresee that medicinal chemistry efforts devoted to the discovery of NS2B-NS3 inhibitors may deliver broad-spectrum antiviral compounds able to inhibit viral infection of members of the same *genus* showing similar antiviral activity.

Orthoflavivirins are generally selective for cleaving at the C-terminal side of a sequence of basic amino acids,^37^ hence it is not surprising that previous research aiming to develop DENV2 NS2B-NS3 competitive inhibitors led to compounds **1** and **2** (Figure 1), peptide-like viral protease inhibitors carrying basic features.^33^ One issue in the development of NS2B-NS3 competitive inhibitors has been the difficulty in translating the viral protease inhibitory potency obtained from a biochemical assay to effective cellular antiviral activity,^38^ because of the low cellular permeability of high molecular weight peptides with multiple charges.^34^ Further optimization of **1** aiming to improve its cellular activity led to compound **2** ^34^ and the recently reported compound **3** (Figure 1),^35^ which showed submicromolar cellular antiviral potency and cellular viral protease inhibition in a replicon assay.^35^ Despite the efforts in optimizing competitive peptidomimetic inhibitors, to date no such compound with in vivo efficacy has been reported.^39^ Our research group has previously described^36,40^ polybasic geminoid compounds such as compounds **4** and **5** (Figure 1/Table 1) which inhibit DENV2 NS2B-NS3 and show antiviral activity in DENV2 infected cells in the micromolar range. In the present work, the identification and optimization of a novel effective lipopeptide scaffold, derived after initial exploration of the SAR of geminoids, is described.

**Figure 1:**
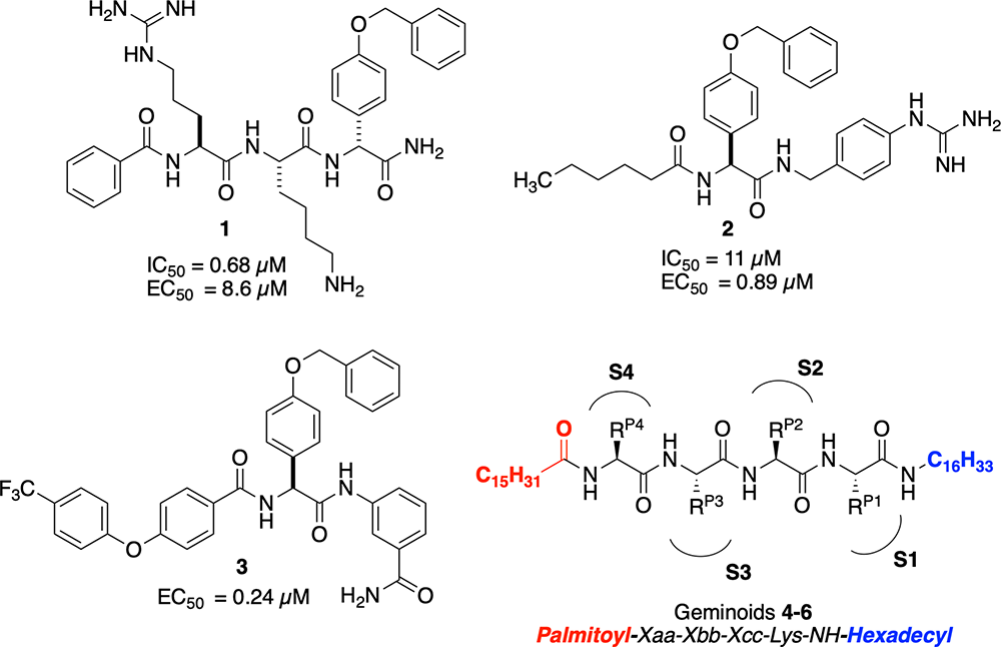
Previously reported^33–36^ DENV2 NS2B-NS3 protease competitive inhibitors showing relevant cellular antiviral EC_50_ values.

**Table 1:** Sequences, NS2B-NS3 Inhibition, and Antiviral Activity of Geminoids **4**–**6** against DENV2 ^36,40^

#	P4	P3	P2	P1	NS2B-NS3 IC_50_ (μM)	EC_50_ (μM)	ref
**4**		Lys	Ala	Lys	2.3	4.1	^36,40^
**5**	Lys	Ala	Ala	Lys	1.4	3.1	^36,40^
**6**	Ala	Arg	Gln	Lys	1.4	1.5	^40^

Geminoids **4**–**6** (Figure 1/Table 1) represent an attractive scaffold for the development of novel orthoflavivirin inhibitors, because of their activity in a cellular infection model and their low cellular toxicity.^36^ They are composed of a short peptide sequence, carrying basic residues, appended with two lipophilic substituents at their ends, a palmitoyl moiety at the N-terminus and a hexadecylamine at the C-terminus (summarized as geminoid scaffold, palmitoyl-XXXK-NH-hexadecyl). Geminoids **4** and **5** carry two Lys residues spaced by one or two Ala residues, respectively, while **6** carries an Arg and Lys, spaced by a Gln, and a N-terminal Ala. The sequence ARQK of geminoid **6** was explored before by our group because of similarity with the autocleavage site between NS2B and NS3,^40^ as reported earlier by Biedrzycka and co-workers.^41^ Attempts to rationalize the binding and inhibition of compounds **4**–**6** to NS2B-NS3 through in silico experiments (namely, docking and molecular dynamics) failed because of the shallow pocket characteristic of orthoflavivirins^42–44^ and the high conformational freedom of the lipophilic palmitoyl and hexadecyl substituents, which hindered the simulation of unambiguous binding poses. Supposedly, the peptidic sequence of **4–6** competes with the substrate of NS2B-NS3 occupying the S1–4 subsites, as schematically represented in Figure 1, given their similarity to other polycationic peptide-like orthoflavivirin inhibitors.^44–47^ The interaction contribution by the lipophilic substituents with NS2B-NS3 is poorly understood. The most relevant interaction for inhibition is supposedly that of the amino acid at the C-terminus (P1) with the S1 subsite. Geminoids **4–6** may not be considered drug-like^48^ because of the two symmetric linear alkyls at their ends and multiple protonable nitrogens, resulting in high molecular weight amphiphiles with a submillimolar critical micelle concentration (CMC).^36^ Micellar aggregation may interfere with the assays measuring inhibitory potency and antiviral activity, and may induce toxicity. The molecular weights of compounds **4–6** range from 806 Da (geminoid **4**) to 962 Da (geminoid **6**), exceeding greatly the 500 Da commonly accepted as drug-likeness parameter.^48^ There are several examples of successfully marketed protease inhibitors (such as HCV and HIV protease inhibitors)^49^ exceeding the 500 Da threshold, but they rarely reach or exceed 800 Da. Furthermore, compounds **4–6** have a very high degree of conformational freedom, with the two long linear alkyl substituents leading to a total of 43 rotatable bonds in the smallest geminoid (**4**). These factors likely result in poor oral bioavailability of the scaffold.^50^

In this work, exploration of the substituents at the N- and C-terminal ends of the geminoid scaffold (R^1^-XXXX-R^2^) in *Library I* led to the identification of the lipopeptide scaffold (palmitoyl-XXXX-NH_2_, Figure 2), having only the N-terminus palmitoylated and a primary amide at the C-terminus. Such derivatives are more drug-like than their parent compounds, and are found in this work to inhibit similarly the NS2B-NS3 protease and viral growth in a DENV2 infection model. We further investigated the SAR of lipopeptide scaffold (palmitoyl-XXXX-NH_2_), exploring the peptidic sequence in *Library II*, first by removing the Ala residues or by substituting them with a constrained amino acid (viz. Pro or Pip). concomitant with the exchange of cationic residues for aromatic residues and exploration of the epimers of the most relevant inhibiting sequences. As a result, two short lipopeptides were identified that efficiently lower the DENV2, ZIKV and WNV viral load in the low micromolar range, while devoid of cellular toxicity. The selected compounds inhibit NS2B-NS3 from DENV2, ZIKV, and WNV but not the host proteases trypsin and thrombin. Additionally, one compound was selected for in vivo pharmacokinetic profiling in BALB/c mice following intravenous (IV), intraperitoneal (IP), and subcutaneous (SC) administration, demonstrating good stability and tolerability.

**Figure 2:**
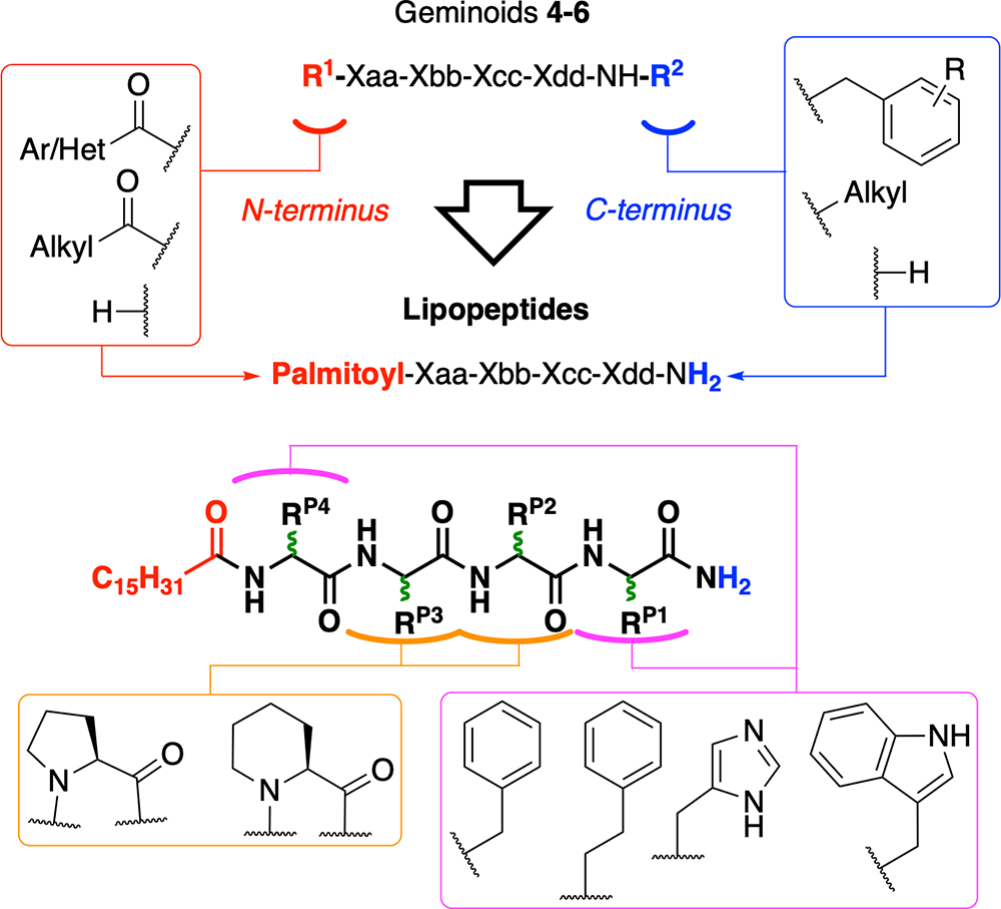
Initial SAR exploration of geminoid and lipopeptide scaffolds. Red rounded rectangle R^1^: moieties explored at the N-terminus. Blue rounded rectangle: moieties explored on the amide at the C-terminus. Magenta rounded rectangle: moieties explored around P1 and P4. Orange rounded rectangle: P^2^ or P^3^ was either removed or substituted by Pro or Pip. Green stereocenters: the respective epimers were explored. Ar: aryl and Het: heteroaryl.

## Results and Discussion

### Chemical Synthesis

Compounds **7**, **8**, **9**, **13**, **14**, **15**, **19**, **20**, **21**, **25**, **26**, **27,** carrying an acylated N-terminus and a C-terminal secondary amide (R^1^-XXXX-NH-R,^2^) were synthesized through a mixture of both SPPS and solution peptide synthesis. As shown in Scheme 1, side chain protected peptide intermediates with a free C-terminal carboxylic acid were obtained using standard semiautomated SPPS, deploying CTC resin,^51–54^ Fmoc amino acids, and DIPCDI/HOBT as coupling reagents.^55,56^ Amino acids carrying reactive side chains which would have interfered with SPPS were protected as follows: the side chain of Fmoc-Lys-OH was Boc-protected, of Fmoc-Arg-OH Pbf-protected, and of Fmoc-Gln-OH and Fmoc-His-OH Trt-protected. The acylation of the N-terminus by the carboxylic acid R^1^-OH required the use of HATU/DIPEA as coupling reagents. Compounds having a free N-terminus and a secondary amide C-terminus (H-XXXX-NH-R^2^, compounds **31, 32, 33, 34, 35, 36**), were synthesized analogously with the exception of the last amino acid, which was introduced as *N* ^*a*^-Boc-protected. After cleavage from the resin, the C-terminus of the protected peptide intermediate was coupled in solution to alkyl amines R^2^-NH_2_, using HATU/DIPEA as coupling reagents. A global deprotection was performed using 95% TFA and a 5% of scavengers (TIPS:H_2_O 1:1). The compounds carrying an acylated N-terminus and C-terminal primary amide (R^1^-XXXX-NH_2_, compounds **10**, **11**, **12**, **16**, **17**, **18**, **22**, **23**, **24**, **28**, **29**, **30**, **37**, **38**, **39, 40–79**), were synthesized via standard semiautomated SPPS, using Rink amide MBHA resin.^57,58^ Peptide coupling conditions and protecting groups deployed were analogous to those described above. Cleavage from the resin, concomitant with the global deprotection of the side chains of the protected amino acids, was performed with 95% TFA in the presence of scavengers (TIPS, water). All compounds were obtained as TFA salts, purified via preparative RP-HPLC, and submitted to lyophilization prior to solubilization for the bioassays. The final compounds were characterized with ^1^H NMR and LCMS (purity > 95%) and when possible, also by ^13^C NMR and HRMS.

**Scheme 1:**
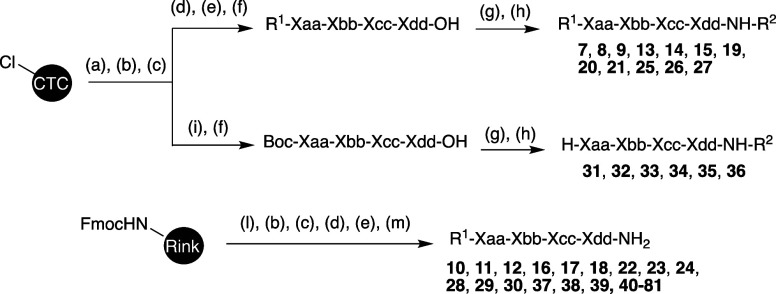
Synthetic Schemes to Obtain the Compounds Covered in This Work^a^

a Reagents and conditions: (a) (1) Fmoc-Xdd–OH, collidine, DCM, 16 h; (2) MeOH, DIPEA, 0.25 h; (3) 20% piperidine, DMF, 0.33 h; (b) (1) Fmoc-Xcc–OH, DIPCDI, HOBT, DMF, 2 h; (2) 20% piperidine, DMF, 0.33 h; (c) (1) Fmoc-Xbb–OH, DIPCDI, HOBT, DMF, 2 h; (2) 20% piperidine, DMF, 0.33 h; (d) (1) Fmoc-Xaa–OH, DIPCDI, HOBT, DMF, 2 h; (2) 20% piperidine, DMF, 0.33 h; (e) R^1^-OH, HATU, DIPEA, DMF, 2 h; (f) 10% AcOH, 10% TFE, DCM, 2 h; (g) R^2^-NH_2_, HATU, DIPEA, DMF, 3 h; (h) 95% TFA, 2.5% TIPS, 2.5% H_2_O, 1.5 h; (i) (1) Boc-Xaa–OH, DIPCDI, HOBT, DMF, 2 h; (l) (1) 20% piperidine, DMF, 0.33 h; (2) Fmoc-Xdd-OH, DIPCDI, HOBT, DMF, 16 h; (3) 20% piperidine, DMF, 0.33 h; (m) 95% TFA, 2.5% TIPS, 2.5% H_2_O, 2 h. The detailed experimental procedures and characterization data are provided in the SI.

### Structure–Activity Relationships

We synthesized 73 peptide-like compounds, which were clustered in two libraries, one where we explored modifications of the C- and N-terminus (library I), and a second investigating several peptide sequences by sequential iterative design (library II). The compounds were screened at 50 μM for inhibition of DENV2 NS2B-NS3 in a biochemical enzymatic FRET-assay;^59,60^ for compounds showing strong inhibition (>60%) an IC_50_ was measured. Selected compounds (generally showing IC_50_ < 10 μM) were tested by applying two different protocols for cellular DENV2 infection assays (Protocol 1, in LLC-MK2 or Vero cells; Protocol 2, in BHK cells), that are equally suitable to determine inhibition of virus replication, as detailed in the SI. For relevant compounds the cytotoxicity was measured as percentage of uninfected cells at specific compound concentration compared to DMSO treated uninfected control cells.

### Library I: N-Terminus and C-Terminus Substituents

In *Library I*, we aimed to investigate how the palmitoyl and *n*-(C_16_H_33_) moieties of geminoids **4–6** affect the inhibition of DENV2 NS2B-NS3, exploring whether one or two alkyl substituents are needed at their ends, or if moieties other than palmitoyl and hexadecyl are tolerated. Peptides KAK, KAAK and ARQK were used as scaffold sequences, from which we designed analogues carrying variations at the N-terminus and the C-terminal amide (R^1^ and R^2^ in Figure 3, respectively). Such variations included compounds carrying substituents at both ends (R^1^-XXXX-NH-R^2^), carrying a substituent only on the N-terminus and a primary amide at the C-terminus (R^1^-XXXX-NH_2_), or having a free N-terminus and secondary alkyl amide at the C-terminus (H-XXXX-NH-R^2^). Acyl substituents at the N-terminus included capping moieties reported previously in analogous polybasic peptidic DENV NS2B-NS3 inhibitors, namely benzoyl,^33,61^ [1,1′-biphenyl]-4-carbonyl,^33^ [2,2′-bithiophene]-5-carbonyl,^34^ palmitoyl and a shorter acyl, namely heptanoyl. Compounds carrying substituents at both ends included those on the amide at the C-terminus having a benzyl moiety substituted to different degrees. We included shorter geminoid analogues carrying an hexyl moiety on the C-terminal amide and an heptanoyl substituent on the N-terminus (heptanoyl-XXXX-NH-hexyl, **25–27**), as well as only the hexyl moiety (H-XXXX-NH-C_6_, **31–33**). As shown in Figure 3 only the compounds that carried a hexadecyl or palmitoyl substituent at the C-terminal amide (**34**–**36,** H-XXXX-NH*-n*-(C_16_H_33_)) or the N-terminus (**37**–**39,** palmitoyl-XXXX-NH_2_), could effectively inhibit DENV2 NS2B-NS3. The substitutions at one or both ends by shorter alkyls (carrying one viz. compounds **28**–**33**, or in geminoids carrying two as compounds **25**–**27**) or aromatic substituents (compounds **7**–**24**) yielded compounds that even at 50 μM showed only 10–30% protease inhibition. It is clear that the palmitoyl or hexadecyl moieties are necessary for inhibition of DENV2 NS2B-NS3. The lipopeptides carrying a C-terminal hexadecyl amide, viz. **34**–**36** or a N-terminal palmitoyl **37**–**39** were selected to have their full DRC and IC_50_ determined.

**Figure 3:**
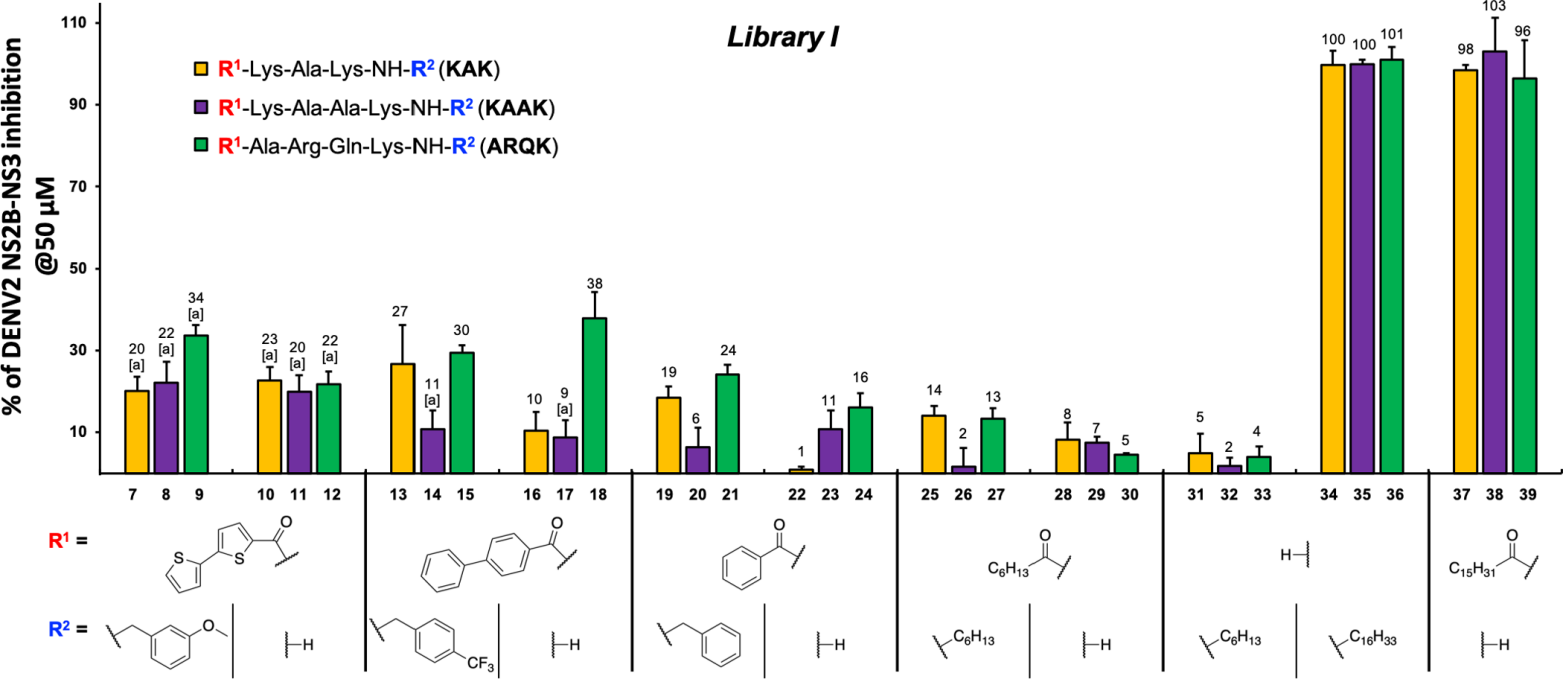
DENV2 NS2B-NS3 percentage of inhibition at 50 μM for compounds in Library I. The percentage of inhibition was measured by a FRET-assay as described by Klein and co-workers.^59,60^ ^[a]^The percentage of inhibition was measured by means of HPLC^60^ because the aromatic substituents appeared to interfere with the fluorometric measurement.

As shown in Table 2 lipopeptides **34**–**39** all inhibit DENV2 NS2B-NS3 similarly in the low micromolar range (IC_50_ = 1.5–3.9 μM). Supposedly, the peptide moieties of lipopeptides **34**–**39** can be recognized by DENV2 NS2B-NS3 as target sequences because of their polybasic features, similarly to **1**, but the main contributor to the inhibition are the palmitoyl/hexadecyl substituents, which can be placed either at the N-terminus or at the C-terminus. Despite the contribution of those lipophilic substituents, lipopeptides **34**–**39** were between about 5–20-fold less potent than **1**, probably because the longer distance between the cationic amino acids of **34**–**39** may not allow binding to the protease as efficiently as observed for **1** (Bz-RK-*p*-BnOPhg-NH_2_). In fact, compounds **22**–**24**, having the same sequences as **34**–**39** and the same N-terminal capping moiety as compound **1** (viz. Bz-XXXX-NH_2_), do not inhibit the protease significantly at 50 μM. The ARQK sequence is 2-fold more efficient in inhibiting the protease than the KAK and KAAK sequences (ARQK range IC_50_ 1.5–2.0 μM; KAK-KAAK range IC_50_ 2.9–3.9 μM), and **36** (H-ARQK-NH*-n*-(C_16_H_33_)) was the most potent in the biochemical assay among the compounds in Figure 3. The Arg residue of the ARQK sequence might allow **36** and **39** to interact with NS2B-NS3 more efficiently than the Lys in P3/P4 of the sequences KAK-KAAK. The guanidinium group of the Arg residue of **36** and **39** does not only have a higher p*K*_a_, but it may also form more hydrogen bonds than the amino group of Lys in **34** and **35**. The amide side chain of the Gln residue of **36** may also contribute more efficiently to binding than the simple methyl of the Ala in the same position of **34** and **35**.

**Table 2:** DENV2 NS2B-NS3 IC_50_, Antiviral EC_50_ against DENV2 and Toxicity for Selected Compounds from Library I with General Structure R^1^-Xaa-Xbb-Xcc-Xdd-NH-R^2^ ^a^

		P4/R^1^	P3	P2	P1				
#	R^1^	Xaa	Xbb	Xcc	Xdd	R^2^	IC_50_ (μM)^b^	EC_50_ (μM)^c^	toxicity^d^
**34**		H	Lys	Ala	Lys	*n*-(C_16_H_33_)	3.1	9.5	TT @ 50 μM
**35**	H	Lys	Ala	Ala	Lys	*n*-(C_16_H_33_)	3.9	3.7	≫50 μM
**36**	H	Ala	Arg	Gln	Lys	*n*-(C_16_H_33_)	1.5	2.0	TTT @ 50 μM
**37**		palmitoyl	Lys	Ala	Lys	H	2.8	40% @ 50 μM^e^	≫50 μM
**38**	palmitoyl	Lys	Ala	Ala	Lys	H	2.9	3.8	TTT @ 50 μM
**39**	palmitoyl	Ala	Arg	Gln	Lys	H	2.3	4.0	TTT @ 50 μM

a Full DRCs are provided in the SI.

b The biochemical IC_50_ was measured following the protocol detailed by Klein and co-workers through FRET.^59,60^

c Antiviral EC_50_ against DENV2 and viability were measured following protocol 1 in LLC-MK2 cells.

d Toxicity is expressed as percentage of not viable cells @ concentration compared to cells treated with DMSO: *T* = mild toxicity (5–25%); TT = medium toxicity (25–50%); and TTT = severe toxicity (>50%).

e EC_50_ ≫ 50 μM, herein, reported the viral load reduction % compared to DMSO treated at highest concentration tested.

Lipopeptides **34–39** were selected to be tested in the DENV2 cellular infection assay (Protocol in LLC-MK cells). As shown in Table 2, all lipopeptides except **37** were able to reduce DENV2 infection in LLC-MK cells, with EC_50_ values in the low micromolar range (EC_50_ range 2.0–9.5 μM), which is comparable to their IC_50_ (Table 2). It may be expected that a cellular EC_50_ is considerably higher than the biochemical IC_50,_ as previously observed for other peptide-like NS2B-NS3 inhibitors, such as compound **1** (see Figure 1, notably, it achieves maximum 50% total viral load reduction at higher concentrations),^34^ because the latter entails a single purified protein, while the former implies crossing several hydrophobic barriers before reaching the target.^45^ It appears that the hydrophobic substituents at the C- or N-terminus of lipopeptides **34**–**39** play a significant role in improving their cellular profile, and we hypothesize that they improve cell permeability and/or induce delivery of the compounds to the specific cellular location where viral NS2B-NS3 inhibition is most relevant (i.e., the endoplasmic reticulum).^62^ Furthermore, it is possible that the lipopeptides in Table 2 inhibit the DENV2 NS2B-NS3 artificial construct used to measure the IC_50_ to a lesser extent than the wild-type present in the DENV2 antiviral cellular infection assay.^38,63^ The artificial construct consists of a fusion protein of NS2B and NS3 joined by a “mixed” Gly/Ser linker (GGGSGGG) holding the heterodimer together;^59,60^ therefore the lipophilic tail of the lipopeptides might have less access to the hydrophobic area between NS2B and NS3 than in the wild-type. Compound **36** (H-ARQK-NH*-n*-(C_16_H_33_)) was the most effective in *library I* (EC_50_ 2.0 μM). The lipopeptides of sequence KAAK **35** (H-KAAK-NH*-n*-(C_16_H_33_)­C_16_) and **38** (palmitoyl-KAAK-NH_2_) could also effectively reduce the DENV2 infection in LLC-MK2 cells (EC_50_ 3.7 and 3.8 μM, respectively) in the low micromolar range. The antiviral activity of **38** (palmitoyl-KAAK-NH_2_) could be a result of the cytotoxicity, and it might be due to a possible off-target interaction with the host machinery. Compounds **39** (palmitoyl-ARQK-NH_2_) and **34** (H-KAK-NH*-n*-(C_16_H_33_)) also showed cytotoxicity but mild (<25%) and only at high concentration (25 μM). Removal of one lipophilic tail from geminoid **4** (palmitoyl-KAK-NH*-n*-(C_16_H_33_)) was detrimental for the cellular antiviral activity, since **37** (palmitoyl-KAK-NH_2_) could not effectively inhibit the infection in cells (EC_50_ ≫ 50 μM, max viral load reduction 40% at 50 μM) and **34** (H-KAK-NH*-n*-(C_16_H_33_)) was approximately 3-fold less potent (EC_50_ 9.5 μM) than its analogues in Table 2. Lipopeptides **37** and **34** inhibit the viral protease in the biochemical assay similarly to geminoids **4**–**6**,^36,40^ suggesting that their reduced antiviral effect is due to an unspecified cellular host metabolism, which does not occur with other sequences or when both palmitoyl/hexadecyl terminal substituents are present. In particular, the N-terminal acylated lipopeptide **37** was the least potent in the cellular infection assay among the compounds in Table 2. From the results in Figure 3, it is clear that a palmitoyl or *-n*-(C_16_H_33_) at the ends of peptide sequences KAK, AQRK and KAAK is essential to show viral protease inhibition. The lipopeptides alkylated on the C-terminal amide (**34–36**; H-XXXX-NH*-n*-(C_16_H_33_)) showed a slightly stronger antiviral effect and slightly lower cytotoxicity in cells than their analogues acylated on the N-terminus (**37–39**; palmitoyl-XXXX-NH_2_). We decided, however, to pursue further the chemotype with the acyl substituent attached to the N-terminus (viz. **37−39**; palmitoyl-XXXX-NH_2_, Figure 2). This choice was principally driven by the hypothesis that the *n*-hexadecylamide moiety of **34–36** might be hydrolyzed by NS2B-NS3 or other proteases, and that the loss of the alkyl substituent would result in loss of inhibitory potency over time. Furthermore, **34–36** carry three protonable nitrogens, making the compounds very dense in cationic features, which might result in poor ADMET profiles in the development pipeline. Finally, peptides with a C-terminal primary amide of general structure palmitoyl-XXXX-NH_2_ could be synthesized swiftly by SPPS alone, using standard Rink amide resin, whilst C-terminal alkylated amide lipopeptides such as **34–36** (H-XXXX-NH*-n*-(C_16_H_33_)) required additional in-solution chemical modifications or the use of a more advanced resin for SPPS. Next, we sought to improve the potency and tolerability of compounds **37–39** exploring analogues varying the peptidic sequence, while keeping the palmitoyl N-terminus and the primary amide C-terminus.

### SAR Library II: Sequence Screening

The compounds presented until now have a molecular weight which exceeds the nominal 500 associated with drug-likeness; therefore we synthesized shorter peptides. We removed one Ala spacer from compounds **37** (palmitoyl-KAK-NH_2_), **38** (palmitoyl-KAAK-NH_2_) and **39** (palmitoyl-ARQK-NH_2_), generating palmitoyl lipopeptide amides with combinations of Lys, Arg and Gln as shown in Table 3 (compounds **40**–**44**). Since cationic short peptides are known substrates of human proteases,^64,65^ we included a Pro residue to constrain the peptide in a horseshoe conformation, thereby making most sequences less susceptible to splitting by common proteases and potentially reducing their off-target activity.^64,66,67^ To this purpose we synthesized lipopeptides **45**–**47** and **60–63**, in which the spacer Ala in the sequences KAK and KAAK was swapped for a Pro residue or its Pip analogue. While the presence of one protonable nitrogen improves water solubility, the presence of two such groups may interfere with their uptake in cells via passive diffusion, in spite of their relatively small size (<800 Da).^68^ Therefore, we explored the removal and/or substitution of one of the cationic amino acids from **37** (KAK), **38** (KAAK), **47** (KPAK) and **39** (ARQK). Previous studies showed successful peptide-based orthoflaviviral NS2B-NS3 protease inhibitors^33–35,69,70^ where the P1 residue was exchanged for an aromatic residue (e.g., compounds **1**–**3,** Figure 1). The crystal structure of NS2B-NS3 ^44^ bound to the covalent inhibitor Bz-nKRR-H shows that the S1 pocket stabilizes the cationic guanidinium group of the P1 residue Arg of the ligand through a cation-pi interaction with a Tyr residue. This suggests that the cationic residues in P1 can be replaced by an aromatic residue to give pi-pi stacking in the S1 pocket. To this end we synthesized compounds **56–63**. Additionally, we included analogues of **37** and **38** where both Lys were substituted by Phe (compounds **54** and **59**), and analogues carrying His or Phg in P2 in parallel with other modifications (**63**–**66**). In order to probe the DENV2 NS2B-NS3 recognition subsites, and potentially improve the cellular tolerability profile, we synthesized analogues of some selected sequences which contained one d-amino acid, screening for P1–P4 positions on selected sequences.

**Table 3:** DENV2 NS2B-NS3 IC_50_, Antiviral EC_50_, and Viability against DENV2 for Compounds in Library II, with General Structure Palmitoyl-Xaa-Xbb-Xcc-Xdd-NH_2_ ^a^

	Xaa	Xbb	Xcc	Xdd	IC_50_ (μM)^b^	EC_50_ (μM)^c^	CC_50_ (μM)^d^	cell line
compound	P4	P3	P2	P1				
**40**			Lys	Lys	4.9	49% @ 50 μM	≫50	LLC-MK2
**41**			Lys	Arg	3.0	37% @ 50 μM	≫50	LLC-MK2
**42**			Arg	Arg	1.8	2.6	12	Vero
**43**			Arg	Lys	2.4	25% @ 50 μM	≫50	LLC-MK2
**44**		Arg	Gln	Lys	4.1	3.5	22	Vero
**45**		Lys	Pro	Lys	5.3	12	≫50	LLC-MK2
**46**	Lys	Ala	Pro	Lys	4.7	31	≫50	LLC-MK2
**47**	Lys	Pro	Ala	Lys	5.2	6.4	≫50	LLC-MK2
**48**			Arg	His	4.6	7.4^e^	T @2 μM	BHK
**49**		Arg	Ala	Phe	2.5	13	≫50	Vero
**50**		Arg	Gln	Hph	2.2	12	≫50	Vero
**51**	Ala	Arg	Gln	Phe	2.5	9.2	≫50	Vero
**52**		Lys	Ala	Phe	2.1 (70%)^f^	6.6^e^	≫50	BHK
**53**		Lys	Ala	His	2.9	5.9	TT @50 μM	LLC-MK2
**54**		Phe	Ala	Phe	n.d. (18%)^f^	-	-	-
**55**	Lys	Ala	Ala	Phe	8.1	<1% @ 10 μM	T @ 2 μM	BHK
**56**	Lys	Ala	Ala	Hph	4.9	<1% @ 10 μM	TT @ 10 μM	BHK
**57**	Lys	Ala	Ala	His	4.1	9.3^e^	TT @ 10 μM	BHK
**58**	Lys	Ala	Ala	Trp	6.8	7.9^e^	TT @ 10 μM	BHK
**59**	Phe	Ala	Ala	Phe	n.d. (<0%)^f^	-	-	-
**60**	Lys	Pro	Ala	Phe	7.5	6.9^e^	T @ 2 μM	BHK
**61**	Lys	Pro	Ala	Hph	7.5	6.6^e^	T @ 5 μM	BHK
**62**	Lys	Pro	Ala	Trp	14	-	-	-
**63**	Lys	Pro	His	Hph	7.0	4.3^e^	T @ 2 μM	BHK
**64**	Lys	Pro	Phg	Lys	3.6	59% @ 25 μM	-	Vero
**65**	Lys	Pip	His	Lys	5.0	16	-	Vero
**66**	Lys	Pip	Phg	Lys	2.6	59% @ 25 μM	-	Vero
**67**		d-Lys	Ala	Lys	8.1	37% @ 50 μM	≫50	LLC-MK2
**68**		Lys	d-Ala	Lys	4.4	31	≫50	LLC-MK2
**69**		Lys	Ala	d-Lys	4.7	2.5% @ 50 μM	≫50	LLC-MK2
**70**		d-Lys	Ala	Phe	4.5	2.6	TTT @ 10 μM	BHK
**71**	d-Lys	Ala	Ala	Lys	3.7	6.3	≫50	LLC-MK2
**72**	Lys	d-Ala	Ala	Lys	5.3	5.9	TTT @ 25 μM	LLC-MK2
**73**	Lys	Ala	d-Ala	Lys	2.4	4.1	≫50	LLC-MK2
**74**	d-Lys	Ala	Ala	Phe	4.4	<1% @ 10 μM	TT @ 10 μM	BHK
**75**	Lys	Pro	Ala	d-Lys	7.7	11	≫50	Vero
**76**	Lys	Pro	Ala	d-Phe	7.8	5.8^e^	≫50	BHK
**77**	Lys	Pro	Ala	d-Hph	7.8	6.6^e^	T @ 5 μM	BHK
**78**	Lys	Pro	Ala	d-Trp	9.0	5.4^e^	≫50	BHK
**79**	d-Lys	Pro	Ala	Trp	8.7	9.0^e^	-	BHK

a - = not measured. Full DRC curves are provided in the SI.

b The biochemical IC_50_ was measured following the protocol detailed by Klein and co-workers through FRET.^59,60^ Unless specified otherwise, an IC_50_ was determined for compounds showing >95% inhibition of NS2B-NS3 at 50 μM.

c EC_50_ was determined only for compounds with >70% viral load reduction @ the highest concentration point tested. For compounds where an EC_50_ was not determined because it was outside the tested concentration range, the viral load reduction % compared to DMSO at the highest concentration point used is reported. Compounds were tested in LLC-MK2 and Vero cells following protocol 1, while in BHK cells following protocol 2.

d When a CC_50_ value was not determined, cytotoxicity is expressed as percentage of not viable cells @ concentration compared to cells treated with DMSO: *T* = mild toxicity (5–25%); TT = medium toxicity (25–50%); and TTT = severe toxicity (>50%).

e EC_50_ was calculated from fitting of a three- or four-point measurement.

f Percentage of DENV2 NS2B-NS3 inhibition at 50 μM.

Among the lipopeptides in Table 2, **54** (palmitoyl-FAF-NH_2_) and **59** (palmitoyl-FAAF-NH_2_) are the only compounds that do not carry nitrogen atoms protonable at physiological pH, and do not inhibit significantly DENV2 NS2B-NS3 at 50 μM. The other compounds in Table 3, carrying at least one protonable nitrogen (Arg or Lys), inhibit NS2B-NS3 with an IC_50_ in the low micromolar range (range IC_50_ = 14–1.8 μM, average IC_50_ = 5.3 μM), similarly or slightly worse (2- to 7-fold less) than their parent compounds (**37–39**, IC_50_ ∼ 2–3 μM). Among those, compound **52**, analogue of **37** carrying a Phe P1, was the only one inhibiting the protease less than 90%. Introduction of a Pro residue on KAK and KAAK sequences resulted in a 2-fold increase in IC_50_. Exchanging the P1 Lys by an aromatic amino acid was well tolerated on compounds carrying an Arg in the scaffold (IC_50_ = 2.2–2.5 μM, compounds **49–52**) while it was detrimental among the compounds **60–64** carrying a Pro residue in P3 (IC_50_ = 7–14 μM). Similar modification on the KAK and KAAK sequence affected the inhibition to varying degrees, resulting in IC_50_ similar or 4-fold higher than their parent compounds. Introduction of a d-amino acid was tolerated at any position, resulting in compounds with similar and up to 4-fold the IC_50_ of their parent epimers. The sequence Arg-Arg (**42**) yielded the most potent lipopeptide in the series (IC_50_ 1.8 μM), inhibiting DENV2 NS2B-NS3 more strongly than parent compound **39**, because Arg may bind stronger than Lys in the recognition subsites of DENV2 NS2B-NS3. In general, Arg containing compounds (either in P1/P2 or P3, such as **41–43** and **49–52**), showed an IC_50_ similar to that of their parent compounds and were among those in Table 3 with IC_50_ < 3 μM. Other notable compounds (IC_50_ < 3 μM) were **73** and **66**, being respectively. the P2 epimer of **38**, and a derivative of **47** where the P3 Pro was exchanged for an analogous Pip, and the P2 Ala was exchanged for an His residue. These results are in line with the recognition subsites of the protease being particularly shallow, which can bind cationic amino acids regardless of their sequence. Furthermore, the relevant pharmacophores identified until now (amine from Lys, guanidinium from Arg and palmitoyl) are installed at the end of or composed of linear alkyl chains which have several rotatable bonds and hence a high-degree of conformational freedom, potentially allowing them to bind by an induced fit in the protease.

Viral protease inhibition did not correlate directly with viral load reduction in the cellular infection models used herein. Potent NS2B-NS3 inhibitors such as Arg containing compounds or **66**, either showed high cytotoxicity (**42** and **44**) or a significantly higher (5- to >100-fold) EC_50_ than their parent lipopeptides. The lipopeptides in Table 2 were generally well tolerated in LLC-MK2, Vero and BHK cells, with most compounds showing no or only mild (**48, 54, 60, 61, 63, 77, 79**) cellular toxicity. It is important to note that most mild and medium to severe toxicity events were observed in BHK cells, as expected due to their higher metabolic rate than LLC-MK2 or Vero cells.^71,72^ While mild cytotoxicity in BHK cells was not considered alarming, medium to severe toxicity in BHK were still considered as disqualifying as in LLC-MK2 and Vero. Cytotoxic compounds such as **56, 57, 58, 53, 70, 72**, and **74** are direct analogues of **37–38** with an aromatic amino acid in P1 and/or a d-amino acid in P3–P4. Interestingly, the Pro analogues of the toxic compounds and of parent compound **38** were less or not cytotoxic. These results suggest sequence specific and/or metabolic cytotoxic pathways to be involved, rather than nonspecific cytotoxicity due to the amphiphilic character of the scaffold. For nontoxic compounds and those for which an EC_50_ could be determined in the tested concentration range, the EC_50_ varied from 4.1 (**73**) to 31 μM (**68** and **46**). In general, the tested compounds show a heterogeneous profile of antiviral activity. Shorter lipopeptides **40–43** showed no EC_50_ within the tested concentration range or cytotoxicity. Removal of Ala from compound **39** resulted in a toxic compound. While the presence of the Ala amino acids from compounds **37–39** does not seem to be relevant for the viral protease inhibition, it affects positively cellular toxicity and antiviral activity of the lipopeptides. Introduction of a Pro or of a d-amino acid on compound **37,** such as in **45** and **68–70** did not improve the antiviral profile, and in the case of a d-amino acid in P1, completely ablated any antiviral activity (**69**). Introduction of a Phe in P1 (compound **52**), improved the antiviral profile compared to **37**, however with an EC_50_ 2-fold lower than compound **38.** On the contrary, derivatives of **38** with an aromatic amino acid in P1 (**54–58, 74**) were inactive, toxic or 2- to 3-fold less active than compound **38.** Introduction of a Pro residue at the P3 position of compound **38** (affording compound **47**) improved the cellular viability, while maintaining a comparable EC_50_. When a Pro was instead introduced in P2 (compound **46**) it resulted in a 10-fold increase in EC_50_. Swapping the Pro for a Pip residue was detrimental for the cellular activity (compounds **65**–**66**). Derivatives of **47** carrying Phe or Hph in P1 instead of Lys (**60** and **61**), showed a similar antiviral activity to compound **47**. Introduction of His in compound **61** did not affect antiviral activity. P1 (compounds **76–78**) and P4 (**79**) epimers of **60–62** showed similar EC_50_ as their parent compounds (**62** was not tested in cells because its IC_50_ was >10 μM). Interestingly the P1 epimer of compound **47** (compound **75**) showed an EC_50_ > 2-fold that of its parent compound. Lipopeptides **40**–**79** showed a heterogeneous antiviral and toxicity profile in a cellular context, with modifications such as epimerization and substitution of Ala with Pro yielding varying effects according to the position in the sequence. Such fluctuations observed in the cellular antiorthoflaviviral SAR suggest a sequence-specific mechanism, rather than nonspecific assay interference.^73^ It should be noted that several compounds (**37**, **40**, **41**, **43**, **46**, **56, 64, 66, 67, 69** and **74**) that were tested in the cellular antiviral assays presented above, did not show significant antiorthoflaviviral effect in spite of their cationic amphiphilic character, substantiating that sequence-specific features play a role in the antiviral mechanism more than physicochemical properties (as e.g., in phospholipidosis^74^). In addition to viral protease inhibition, the cellular SAR of the lipopeptide scaffold may be affected by structure specific processes in the host cell, such as intra/subcellular delivery^62,75^ and metabolic processing,^67,76–79^ or from the viral maturation process, as inhibition of critical protein–protein interactions during viral polyprotein processing.^80^ Further studies are required to substantiate that the antiviral activity of compound **73** and similar lipopeptides is mediated by NS2B-NS3 inhibition.

This sequence screening allowed to improve the cellular antiviral profile of lipopeptide **38**, yielding compound **73.** The latter compound showed antiviral and tolerability profiles in DENV infected cells similar to its parent geminoid **5**. The SAR shows in general that modification of the KAAK sequence was more effective than modifications on the KAK and ARQK lipopeptides. Introduction of a Pro in P3 or of epimerization of P2 seem to be the most promising modifications on the sequence of **38.** Introduction of a Pro residue allows further modification in P1 without affecting the antiviral activity, resulting however in lipopeptides still less active than compound **73**.

### Broad-Spectrum Antiorthoflaviviral Properties and Host Proteases Selectivity

Lipopeptides **73**, **78** and **79** were selected for further screening against NS2B-NS3 from WNV and ZIKV: **73** because it showed the most promising antiviral cellular profile against DENV2, **78** and **79** because of the indolyl of their P1 Trp resembles the benzimidazole fragment that has been reported^81^ to fit efficiently in the S1 subsite of ZIKV NS2B-NS3. As shown in Table 4, compounds **73**, **78** and **79** inhibited NS2B-NS3 from WNV and ZIKV in the low micromolar range; in particular, the inhibition of the ZIKV viral protease correlated well with that of DENV2. These results are consistent with the established conserved structure of NS2B-NS3 among the *genera*,^27,32^ allowing to expand the scope of the DENV2 antiviral lipopeptides described here to broad-spectrum antiorthoflavivirals. Compound **73** was the most potent inhibitor of ZIKV protease in Table 4 (IC_50_ = 1.9 μM), while **79** was the most potent against WNV protease (IC_50_ = 6.7 μM). Lipopeptide **78** inhibited WNV and ZIKV NS2B-NS3 less potently than **79** or **73** and was therefore not tested further in the cellular infection models. The lipopeptides in Table 4 were also tested for trypsin and thrombin inhibition, and none of the compounds inhibited significantly either of the host serine proteases (inhibition of trypsin at 50 μM ≤ 16% and thrombin 25 μM ≤ 5%). This indicates that the lipopeptide scaffold is promising for further development as it shows selectivity toward the viral protease. Both lipopeptides **73** and **79** exerted strong antiviral activity against WNV and ZIKV in the low micromolar range. The antiviral EC_50_ of **73** in cells was similar among DENV, WNV or ZIKV (respectively EC_50_ 4.1, 4.9, 5.0 μM) infection, while **79** was more active against ZIKV (EC_50_ 1.7 μM) and WNV (EC_50_ 3.2 μM) than against DENV2 (EC_50_ 9.9 μM). Lipopeptides **79** and in particular **73** represent novel attractive scaffolds for the development of broad-spectrum antiorthoflavivirals, given their activity in cellular infection models of DENV2, WNV and ZIKV, and high cellular tolerability.

**Table 4:** DENV2, WNV, ZIKV NS2B-NS3 IC_50_,^a^ Cellular Antiviral EC_50_ against WNV and ZIKV,^b^ and Trypsin, Thrombin Inhibition for **73**, **78**, and **79**

	WNV		ZIKV			
#	NS2B-NS3 IC_50_ (μM)	EC_50_ (μM)	NS2B-NS3 IC_50_ (μM)	EC_50_ (μM)	trypsin at 50 μM	thrombin at 25 μM
**73**	7.2	4.9	1.9	5.0	11% ± 12.3	0%
**78**	13		8.8		16% ± 7.7	0% ± 4.2
**79**	6.7	3.2	8.5	1.7	6% ± 5.2	5% ± 0.8

a All compounds showed >95% inhibition of NS2B-NS3 at 50 μM. Full DRC are reported in the SI. The biochemical IC_50_ and of N2B-NS3 inhibition was measured following the protocol detailed by Klein and co-workers through FRET.^59,60^ Analogous procedures were used to obtain inhibition values of the human proteases trypsin^82^ and thrombin,^59^ and the other orthoflavivirins WNV NS2B-NS3 ^34^ and ZIKV NS2B-NS3.^83^

b EC_50_ measured in infected Vero cells.

### Kinetics of Inhibition of Compound **73**

The kinetics of NS2B-NS3 inhibition by compound **73** were assessed in the FRET biochemical assay as described by Klein and co-workers,^60^ evaluating potential IC_50_ shifts at different substrate concentrations (50–100 μM). As shown in Figure 4A, the IC_50_ of compound **73** remained consistently around 7 μM (SD < 0.2 μM) with a minor decrease at the highest tested substrate concentration (200 μM). This indicates noncompetitive binding kinetics in which the enzymatic activity is reduced by the inhibitor binding irrespective of the presence, absence, or concentration of the substrate. Based on this interpretation and the Cheng–Prussof plot, the *K*_i_ value of the tested compound is identical to the IC_50_ (*K*_i_ = 7 μM at 100 μM substrate concentration). It is important to note that the IC_50_ value reported in Table 3, measured under nominally analogous conditions at 50 μM substrate concentration, differed slightly from this kinetic study. In each assay run was also included compound **1** as reference, which did not show the same variability. This discrepancy may reflect unique sensitivity of compound **73** to subtle differences in assay conditions and its distinct binding kinetics or inhibitory mechanism, which can influence apparent potency in a manner not observed for the reference inhibitor, compound **1**.^84,85^ Although the noncompetitive binding mode suggests that compound **73** might not be an orthosteric inhibitor of DENV2 NS2B-NS3, previous studies have reported orthosteric NS2B-NS3 inhibitors which inhibit the protease noncompetitively.^38^ Further studies are required to understand whether compound **73** interacts with the active site at all, or binds to an allosteric site of the protease.

**Figure 4:**
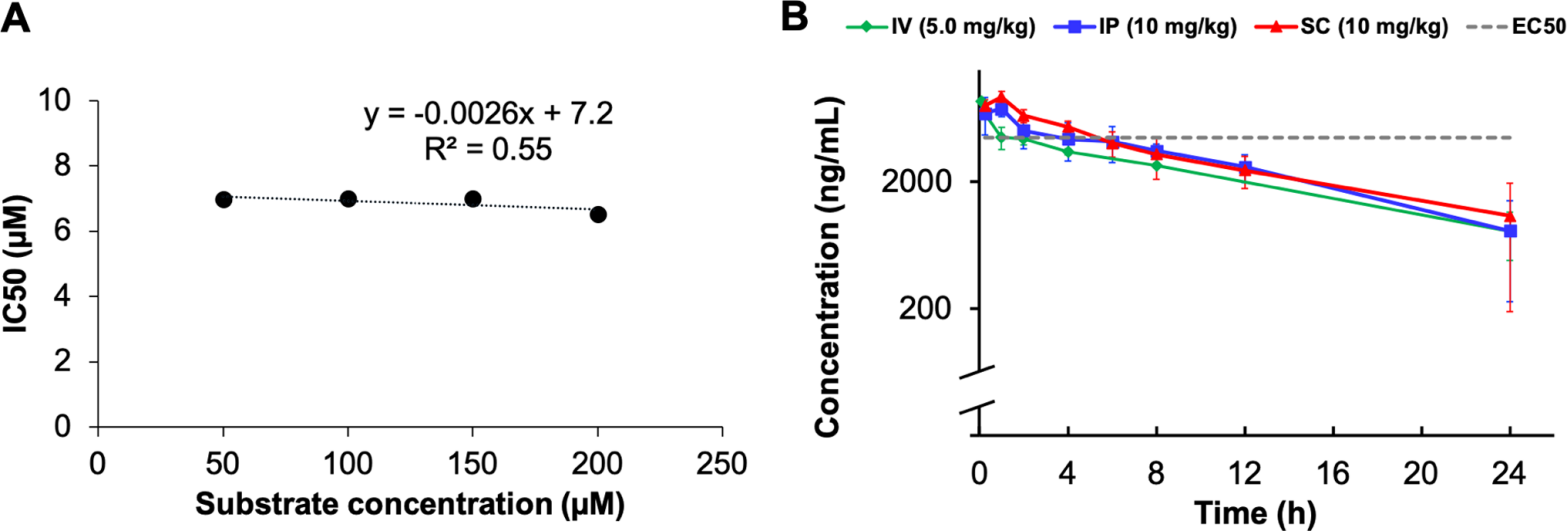
(A) Cheng–Prussoff plot for compound **73**. Linear regression calculated with MS Excel, taking in consideration only points 50–150 μM, *R* ^2^ = 0.96. (B) Plasma concentration over time (24 h) of compound **73** after single administration to female C57BL/6 Mice (*N* = 3).

### In Vivo PK of Compound **73**

The pharmacokinetic profile of compound **73** was assessed in female C57BL/6 mice (*N* = 3) following single administration by three routes: intravenous (IV) at a dose of 5 mg/kg, and intraperitoneal (IP) and subcutaneous (SC) routes at a dose of 10 mg/kg. Pharmacokinetic profiling was performed prior to in vivo efficacy studies, as is standard practice, to establish compound stability and tolerability and to ethically determine appropriate dosing levels before disease models are pursued. All animals were healthy throughout the study. The plasma concentration vs time profile is depicted in Figure 4B, and key pharmacokinetic parameters are summarized in Table 5. Following IV administration, **73** displayed predictable pharmacokinetics with a moderate elimination half-life (*t*_1_/_2_) of approximately 9.8 ± 2.8 h and a low clearance rate of 1.27 ± 0.25 mL/min/kg. The volume of distribution (*V*_d_) was close to total body water, suggesting limited distribution into peripheral tissues and consistent systemic exposure.

**Table 5:** Pharmacokinetic Parameters of 73 upon IV, IP, and SC Administration to Female C57BL/6 Mice (*N* = 3)^a^

route	IV	IP	SC
dose (mg/kg)	5	10	10
*C*_0_/*C*_max_ (ng/mL)	9600 ± 310 (3%)	8300 ± 1370 (17%)	9300 ± 1020 (11%)
*T*_max_ (h)		0.8 ± 0.4 (58%)	1
*t*_1_/_2_ (h)	9.8 ± 2.8 (29%)	7.7 ± 3.3 (42%)	28 ± 37 (130%)
AUC_0‑last_ (ng h/mL)	55,100 ± 5200 (9%)	67,900 ± 17,600 (26%)	73,700 ± 6,900 (9%)
AUC_0‑infinite_ (ng h/mL)	67,400 ± 12,100 (18%)	78,900 ± 28,500 (36%)	147,000 ± 113,000 (77%)
Cl or Cl/F (mL/min/kg)	1.27 ± 0.25 (20%)	2.30 ± 0.81 (35%)	1.57 ± 0.84 (53%)
*V*_d_ or *V*_d_/F (L/kg)	1.03 ± 0.13 (13%)	1.41 ± 0.19 (14%)	2.05 ± 1.50 (73%)
MRT (h)	8.3 ± 0.8 (9%)	8.2 ± 0.6 (7%)	7.9 ± 1.2 (15%)
%*F*	100	57 ± 19 (33%)	63 ± 4 (6%)

a Values are presented as mean ± standard deviation (SD), with the coefficient of variation (%CV) in parentheses.

IP and SC administrations resulted in a bioavailability of 57 ± 19% and 63 ± 4%, respectively, compared to IV. The apparent volumes of distribution (*V*_d_/F) were increased for both routes compared to IV administration, particularly upon SC administration (2.05 ± 1.50 L/kg). Notably, the SC route demonstrated significant variability in both elimination half-life and volume of distribution, with coefficients of variation (%CV) of 130 and 73%, respectively, indicating inconsistent absorption kinetics and distribution among subjects. This variability may be attributed to interindividual differences in subcutaneous tissue composition, which can influence the rate and extent of drug absorption. Differences in tissue binding, sequestration within adipose tissue, or variable permeability of the subcutaneous tissue may lead to a depot effect with variable release rates, affecting both the duration of action and systemic exposure. A depot effect could result in flip-flop kinetics, where the rate of absorption is slower than the rate of elimination, explaining the longer and more variable half-life upon SC administration. Degradation at the administration site or presystemic metabolism may also affect variability due to differences in subcutaneous enzymatic activity, altering the amount of drug reaching systemic circulation. Additionally, the physicochemical properties of **73**, such as its high molecular weight and amphiphilic character, may influence particularly its absorption and distribution, contributing to fluctuations in pharmacokinetic parameters. Since DENV at first infects cells in peripheral tissues, the higher tissue distribution (*V*_d_) following SC administration may be beneficial enhancing its therapeutic efficacy. Furthermore, the depot effect could provide sustained release of the drug and prolonged exposure at the target sites. Based on the plasma concentration–time profiles, at the current dosages, **73** sustains concentrations above the EC_50_ for about 6 h following SC and IP administration routes. These observations suggest that doubling the dosage of **73** (20 mg/kg for IP and SC routes) and administering it twice daily may be necessary to achieve sustained therapeutic efficacy. Compound **73** is stable and well-tolerated in vivo, and exhibits a promising pharmacokinetic profile, suggesting that SC administration could offer therapeutic advantages. However, the high variability in pharmacokinetic parameters with SC administration may impact the predictability of therapeutic outcomes. Further studies investigating formulation approaches, such as controlled-release formulations or absorption enhancers, may help reduce this variability and improve in vivo efficacy.

## Conclusion

In this work, we have optimized the geminoid scaffold (compounds **4–6**, palmitoyl-XXXX-NH*-n*-C_16_H_33_, containing KAK, KAAK and ARQK, respectively, Table 1), obtaining more drug-like derivatives with comparable viability and antiviral EC_50_ in vitro*,* and good stability and tolerability in vivo. The SAR revealed that one alkyl substituent (either C-terminal or N-terminal) is required to exert viral protease inhibition and cellular antiviral activity. Therefore, we identified short *N*-palmitoylated lipopeptides (palmitoyl-XXXX-NH_2_) as a novel scaffold of interest. We optimized the peptide sequence including constrained amino acids, swapping the cationic residue in P1 for an aromatic amino acid and including epimers carrying one d-amino acid at various positions of the peptide sequence. The KAAK sequence emerged as most promising, showing that P3 substitution with a Pro (**47**) or epimerization of P2 (**73**) resulted in improved cellular profile. After several iterations of in vitro experiments and SAR exploration of the lipopeptide scaffold, compounds **73** and **79** emerged as NS2B-NS3 inhibitors which exerted antiorthoflaviviral activity in the low micromolar range in DENV2, WNV and ZIKV cellular infection models. Although **79** was more effective against WNV and ZIKV than **73**, the latter showed higher broad-spectrum antiorthoflaviviral activity. Lipopeptide **79** and in particular **73** showed antiviral potency similar to that of their parent geminoids **4–6**, while being more drug-like. The fluctuations in the cellular antiviral activity observed while exploring the SAR of the lipopeptide scaffold suggest a sequence-dependent SAR, rather than unspecific assay interference due the cationic amphiphilic character of the compounds.^73,74^ Lipopeptides **73** and **79** do not inhibit the serine host proteases trypsin and thrombin and are well tolerated in cells. Binding kinetic studies in the used biochemical NS2B-NS3 inhibition assay revealed that compound **73** is a noncompetitive inhibitor. Further structural studies are required to understand the mechanism of NS2B-NS3 inhibition by **73**. Compound **73** exhibits favorable pharmacokinetics and good tolerability in vivo, following single administration of 5 mg/kg IV and 10 mg/kg SC or IP in C57BL/6 Mice. SC administration provided advantages in tissue distribution and prolonged plasma levels. Lipopeptide **73** maintains plasma concentrations above the EC_50_ for 6 h when administered IP and SC, suggesting a dosing regimen of twice daily 20 mg/kg to achieve therapeutic efficacy in mice. To enable translation in humans, new strategies to improve scaffold potency are required, along with further experiments to assess oral bioavailability. Nevertheless, we warrant further in vivo studies in animals to fully evaluate and confirm therapeutic efficacy of **73** against DENV2, WNV and ZIKV infection. The lipopeptide scaffold described herein, and in particular compound **73**, represent promising leads for the development of broad-spectrum antiorthoflaviviral agents.

## Experimental Section

### Chemistry

For the detailed synthesis and characterization of all compounds we refer to the Supporting Information. Herein we report in brief the general procedures, synthesis and characterization of compound **73**.

#### General Procedures

Standard semiautomated SPPS was performed in empty open-top column cartridges with a plastic frit, which were agitated via an orbital shaker. All peptides were purified via RP-HPLC (unless stated otherwise) using a Shimadzu LC-20A Prominence system. All final compounds are >95% pure by HPLC analysis. LCMS and HPLC spectrograms were recorded on a Thermo Finnigan LCQ-Fleet ion trap mass spectrometer (ESI-IT-MS) coupled to a Shimadzu analytical HPLC [LC-20AD (pump) and SPD-M30A (photodiode array detector)], equipped with a Gemini C18 110A column, 50 mm × 2 mm, particle size 3 μm (Phenomenex, Utrecht, The Netherlands), eluting with 0.1% formic acid in a MeOH/Milli-Q H_2_O solution (isocratic 5% MeOH in H_2_O over 5 min, gradient from 5 to 95% MeOH in H_2_O over 20 min, with a solvent flow rate of 1.0 mL/min). NMR spectra were recorded using either a Bruker Avance 400 (400 MHz) or a Bruker Avance III (500 MHz) spectrometer, in D_2_O, MeOH-*d*_4_, CDCl_3_, or DMSO-*d*_6_ solutions, unless stated otherwise. Chemical shifts are given in ppm with respect to residual nondeuterated solvents or TMS as internal standard for CDCl_3_. Coupling constants are reported as *J*-values in Hz. The following abbreviations are used to explain multiplicities: s = singlet, d = doublet, t = triplet, q = quartet, dd = doublet of doublets, ddd = doublet of doublet of doublets, dtd = doublet of triplet of doublets, td = triplet of doublets, m = multiplet, br = broad signal. High resolution mass spectra (HRMS) were recorded on a JEOL AccuToF CS JMS-T100CS (ESI-HRMS).

#### Fmoc Deprotection

The resin was swollen with DCM (10 mL/gram of resin, 1 min) and DMF (2 × 10 mL/gram of resin, 1 min) and treated with 20% piperidine in DMF (10 mL/gram of resin) and left to shake for 20 min. The suspension was filtered and the resin was washed with DMF (2 × 10 mL/gram of resin, 1 min) and treated with a second portion of 20% piperidine in DMF (10 mL/gram of resin) and left to shake for 10 min. The suspension was then filtered and the resin was washed with DMF (3 × 10 mL/gram of resin, 1 min), DCM (3 × 10 mL/gram of resin, 1 min) and MeOH (3 × 10 mL/gram of resin, 1 min). Deprotection efficiency was determined by means of Kaiser or chloranil tests (for Pro deprotection).

#### Loading of the First Amino Acid (Rink Amide MBHA Resin)

The resin was swollen with DCM (1 min) and DMF (2 × 1 min). Fmoc–amino acid (3 equiv) and HOBt (3 equiv) were dissolved in DMF (10 mL/gram of resin) and the resulting solution was added to the resin. Next DIPCDI (3 equiv) was added and the reactor was left to shake for 16 h. The suspension was then filtered and the resin was washed with DMF (3 × 1 min), DCM (3 × 1 min), before treating with a capping solution of acetic anhydride/pyridine (3:2, 10 mL/gram of resin) for 20 min The suspension was then filtered and the resin was washed with DMF (3 × 1 min), DCM (3 × 1 min) and MeOH (3 × 1 min). Coupling efficiency was determined by means of a Kaiser test.

#### Peptide Coupling

The resin was swollen with DCM (1 min) and DMF (2 × 1 min). Fmoc–amino acid (3 equiv) and HOBt (3 equiv) were dissolved in DMF (10 mL/gram of resin) and the resulting solution was added to the resin. Next DIPCDI (3 equiv) was added and left to shake for 3 h. The suspension was then filtered and the resin was washed with DMF (3 × 1 min), DCM (3 × 1 min) and MeOH (3 × 1 min). Coupling efficiency was determined by means of a Kaiser or chloranil tests (for couplings on Pro).

#### Coupling to Palmitic Acid

The resin was swollen with DCM (1 min) and DMF (2 × 1 min). Palmitic acid (3 equiv) and HATU (2.9 equiv) were dissolved in DCM/DMF (1:1, 10 mL/gram of resin) and the resulting solution was added to the resin. Next DIPEA (3 equiv) was added and the reactor was left to shake for 3 h. The suspension was then filtered and the resin was washed with DMF (3 × 1 min), DCM (3 × 1 min) and MeOH (3 × 1 min). Coupling efficiency was determined by means of Kaiser or chloranil tests (for couplings on Pro).

#### Peptide Cleavage (Rink Amide MBHA Resin)

The peptidyl-resin was washed with DCM (3 × 1 min) and dried under nitrogen. The resin was treated with a cleavage solution (95% TFA, 2.5% TIPS, 2.5% H_2_O, 5 mL) and left to shake for 2 h (unless stated otherwise). The mixture was filtered and the resin was washed with DCM (3 × 1 min), filtrates were collected, combined and volatiles were removed in vacuo. The crude residue was triturated in dry diethyl ether and after centrifuge the precipitate was collected by decantation. Solvent leftovers were removed under high-vacuum. The crude material was dissolved in minimal amount of MeOH (unless stated otherwise), filtered through a 0.20 μm syringe filter, and purified using preparative RP-HPLC (isocratic 20% MeCN in H_2_O over 5 min, gradient from 20 to 80% MeCN in H_2_O over 15 min, with a solvent flow rate of 10.0 mL/min, at 30 °C), unless stated otherwise. All fractions containing product were combined, concentrated to 5 mL in vacuo, prior to lyophilization to obtain the pure materials

**Palmitoyl-Lys-Ala-** d **-Ala-Lys-NH**_**2**_ **bisTFA (73)** was synthesized according to the general procedure for SPPS, using MBHA Rink amide resin (0.30 g). After purification via RP-HPLC, compound **73** (83 mg, 45%) was obtained as a white powder. ^1^H NMR (400 MHz, MeOH-*d*_4_): δ 4.39–4.20 (m, 4H), 3.02–2.84 (m, 4H), 2.27 (dd, *J* = 8.3, 6.9 Hz, 2H), 2.03–1.55 (m, 10H), 1.55–1.17 (m, 34H), 0.97–0.84 (m, 3H). ^13^C NMR (101 MHz, MeOH-*d*_4_): δ 177.0, 176.9, 175.3, 175.2, 174.4, 54.8, 54.3, 51.2, 50.8, 40.7, 40.6, 36.9, 33.2, 32.4, 32.3, 30.94, 30.93, 30.90, 30.8, 30.7, 30.6, 30.5, 28.6, 28.1, 27.0, 24.0, 23.89, 23.88, 17.6, 14.6. HRMS (ESI): *m*/*z* calcd for C_34_H_67_N_7_O_5_^+^ [M + H]^+^, 654.5276; found 654.5275. Analytical RP-HPLC, method A (per general procedures) *t*_R_ = 13.54 min; purity = 96,47% (PDA, 210 nm); method B [XBridge Peptide C18, 300 Å column, 4.6 × 250 mm, particle size 5 μm; eluting with 0.1% TFA in MeCN/Milli-Q H_2_O solution (gradient from 30% to 95% MeCN in H_2_O over 14 min, isocratic 95% until 20 min, with a solvent flow rate of 1.0 mL/min)] *t*_R_ = 10.341 min; purity = 99,66% (PDA, 210 nm). NMR and HPLC traces for compound **73** are included in the Supporting Information.

### Biology

For the complete experimental procedures to perform the biological characterization of the compounds, we refer to the Supporting Information.

#### Biochemical Protease Assays

Single-dose screening of compounds and/or IC_50_ determination was performed by a fluorimetric assay for DENV protease as described before.^59,60^ In brief, activity was measured with a FRET substrate (2-Abz-Nle-Lys-Arg-Arg-Ser-(3-NO_2_)-Tyr-NH_2_; *K*_m_ 105 μM). Compounds were serially diluted from 10 mM DMSO stocks and tested in triplicate in 50 mM Tris-HCl pH 9.0, 10% v/v ethylene glycol, and 0.0016% Brij 58. Inhibitors were preincubated 15 min with DENV protease (100 nM), reactions were initiated by substrate addition (50 μM; 100 μL per well), and fluorescence was recorded for 15 min at Ex 320 nm/Em 405 nm. Initial rates (RFU/s) were fitted to derive IC_50_ values. Assays for WNV^34^ and ZIKV^83^ proteases and selectivity counterscreens against human trypsin^82^ and thrombin followed the same format; full protocols are provided in the Supporting Information.

#### Cellular Viral Infection Assays

##### Protocol 1, LLC-MK2 Cells

DENV2 immunoperoxidase (IPOX) assay, in brief. LLC-MK2 cells (CCL-7.1) were seeded at 1 × 10^5^ cells per well (96-well plates) in EMEM-based assay medium and incubated overnight at 37 °C/5% CO2. Compounds (DMSO stocks) were tested as duplicate eight-point, 2-fold serial dilutions starting at 50 μM; ribavirin served as a positive control. Cells were infected with DENV2 (New Guinea strain; 100 TCID_50_ per well), incubated 2 h, washed, and overlaid with compound dilutions. After 48 h, infection was quantified by NS1 immunoperoxidase staining (primary anti-NS1, HRP secondary, AEC chromogen), and virus-positive cells were counted microscopically. Percent inhibition was calculated versus DMSO virus controls, and EC_50_ values were obtained from fitted dose–response curves. Visual cytotoxicity scoring was recorded in parallel. Full protocol in the Supporting Information.

##### Protocol 1, Vero Cells

DENV2 immunofluorescence assay, in brief. Vero cells were plated at 1.5 × 10^4^ cells per well (DMEM, 10% FBS), infected the next day with DENV2 (1000 TCID_50_ per well; 90 min adsorption), washed, and treated with duplicate eight-point, 2-fold serial dilutions of compounds (50 to 0.4 μM). Plates contained virus, cell, positive, and negative controls. After ∼48 h, cells were fixed, permeabilized, stained for NS3 (primary) and Alexa Fluor 488 (secondary), counterstained with DAPI, imaged on a Cytation1 V reader, and quantified in Gen5. Percent inhibition was calculated from infected-cell counts relative to virus controls, and EC_50_ values were derived from fitted curves. Cytotoxicity (MTT): Vero cells were plated as above and exposed to duplicate eight-point, 2-fold serial dilutions of compounds (50 to 0.4 μM) for ∼48 h. MTT was added, formazan was solubilized, and absorbance was read at 570/≥650 nm. Percent viability was calculated versus untreated cells, and CC_50_ values were obtained from fitted curves. Details in the Supporting Information.

##### Protocol 2, Anti-Dengue Cytoprotection (BHK-21/XTT)

in brief, BHK-21 cells were plated at 5 × 10^3^ cells per well in DMEM-based assay medium and incubated overnight. Compounds were tested as single doses and/or five half-log dilutions (to 50 μM; triplicate). Cells were infected with DENV2 (New Guinea strain) at an input yielding 85–95% virus-induced cell death at day 6. After incubation, XTT/PMS was added for 4 h, plates were mixed, and absorbance was read at 450/650 nm. Antiviral efficacy (CPE protection) and compound toxicity were calculated relative to DMSO controls to derive EC_50_ and CC_50_ values. Full method in the Supporting Information.

##### Protocol 3, WNV/ZIKV Focus-Forming Assay

in brief, Confluent Vero E6 monolayers were infected with 50–150 ffu of WNV (NY99) or ZIKV (Dominican Republic/2016/PD1) in the presence of 2-fold serial dilutions of compounds (50 to 0.39 μM). After 1 h from the inoculation, cells were overlaid with compound dilutions in DMEM/2% FCS/1.2% Avicel for 24–30 h. Cells were fixed, permeabilized, and stained with 4G2 primary and HRP-conjugated secondary antibodies; foci were developed with TrueBlue and counted on an ELISpot reader. ffu were normalized to DMSO controls, and EC_50_ values were calculated by variable-slope fits (GraphPad Prism). Qualitative cytotoxicity was monitored in parallel. Full details in the Supporting Information.

#### In Vivo Pharmacokinetics

The in vivo pharmacokinetic studies were conducted at Aragen Life Sciences Pvt Ltd. (Hyderabad, India), a fully AAALAC International accredited facility that operates in accordance with the *Guide for the Care and Use of Laboratory Animals*.^86^ All animal experiments were performed under institutional ethical guidelines and approvals in place at Aragen. A copy of the current AAALAC accreditation certificate has been provided as supporting documentation. No human studies were involved in this work; therefore, informed consent was not applicable. In brief. Female C57BL/6 mice (*n* = 3 per route) received compound **73** as 5 mg/kg IV (tail vein; 20% HPβCD + 5% DMSO) or 10 mg/kg IP/SC (20% HPβCD). Blood was collected at 0.083–24 h (IV) or 0.25–24 h (other routes), plasma was prepared, and compound was quantified by LC–MS/MS using telmisartan as internal standard. Pharmacokinetic parameters were obtained by noncompartmental analysis (Phoenix). Full protocol and bioanalytical conditions in the Supporting Information.

## Supplementary Material





## Notes

Experimental procedures and characterization of all compounds; NMR and HPLC traces for compounds **73** and **79**; and DRC curves (PDF)
Molecular formula strings and the associated biochemical and biological data (CSV)

## Abbreviations

AAALAC: Association for Assessment and Accreditation of Laboratory Animal Care
AcOH: acetic acid
Ala: alanine
Arg: arginine
AUC_0-infinite_: area under the plasma concentration–time curve from time zero to infinity (ng h/mL)
AUC_0-last_: area under the plasma concentration–time curve from time zero to the last measurable concentration (ng h/mL)
BHK: baby hamster kidney
Boc: *tert*-butyloxycarbonyl
C: capsid protein
*C*_0_/*C*_max_ (ng/mL): initial concentration or maximum plasma concentration
Cl or Cl/F (mL/min/kg): clearance or apparent clearance
CTC: chlorotrityl chloride
DBU: 1,8-diazabicyclo­[5.4.0]­undec-7-ene
DCM: dichloromethane
DENV: dengue virus
DIPCDI: diisopropyl carbodiimide
DIPEA: *N,N*-diisopropyl-*N*-ethylamine
DMF: *N,N*-dimethylformamide
DMP: Dess–Martin periodinane
DMSO: dimethyl sulfoxide
DRC: dose response curve
E: envelope protein
EC_50_: half-maximal effective concentration
Fmoc: fluorenylmethyloxycarbonyl
FRET: fluorescence resonance energy transfer
Gln: glutamine
HATU: hexafluorophosphate azabenzotriazole tetramethyl uronium
HCV: hepatitis C virus
HIV: human immunodeficiency virus
HOBT: hydroxybenzotriazole
Hph: homophenylalanine
IC_50_: half-maximal inhibitory concentration
IP: intraperitoneal
IV: intravenous
LLC-MK2: rhesus monkey kidney cells
Lys: lysine
MBHA: 4-methylbenzhydrylamine
Me: methyl
MeOH: methanol
MRT (h): mean residence time
NS: nonstructural
Pbf: 2,2,4,6,7-pentamethyldihydrobenzofuran-5-sulfonyl
Ph: phenyl
Phe: phenylalanine
Pip: pipecolic acid
Pro: proline
RP-HPLC: reversed-phase high-performance liquid chromatography
SAR: structure–activity relationship
SC: subcutaneous
SPPS: solid-phase peptide synthesis
TEA: triethyl amine
TFA: trifluoroacetic acid
TFE: trifluoroethanol
THF: tetrahydrofuran
TIPS: triisopropyl silane
*T*_max_ (h): time to reach maximum plasma concentration
TMS: trimethyl silane
Trt: trityl
TsCl: tosyl chloride
*V*_d_ or *V*_d/F_ (L/kg): volume of distribution or apparent volume of distribution
WNV: West Nile virus
ZIKV: Zika virus
%*F*: percentage bioavailability
