# Supplementary material for: Development of Lipopeptides as Orthoflavivirin Inhibitors with Low Micromolar Broad-Spectrum Antiorthoflaviviral Activity
Source: J Med Chem. 2025 Oct 16;68(21):22386–400. doi: 10.1021/acs.jmedchem.5c01364 (PMC12621197; doi:10.1021/acs.jmedchem.5c01364)
Supplement: Supplementary file 1 [file jm5c01364_si_001.pdf]

# Development of lipopeptides as orthoflavivirin inhibitors with low-micromolar broad-spectrum anti-orthoflaviviral activity

Lorenzo Cavina,[a,*] Anna Alocén Portillo,[a] Mike P. A. Balmer,[a] Jenny C. Dammer,[a] Danae Schillemans,[a] Said Hakim Hamdani,[b] Bart Ackerschott, [b] Cindy E. J. Dieteren,[b] Byron E. E. Martina,[b] Bernd N. M. van Buuren,[b] Alexandra Rockstroh,[c] Sebastian Ulbert,[c] Pedro H. H. Hermkens,[d] Montse Llinàs Brunet[b], Daniel Gironés,[a,b] Martin C. Feiters,[a,] * Floris P. J. T. Rutjes.[a,] *

a) Institute for Molecules and Materials, Radboud University, Heyendaalseweg 135, 6525 AJ Nijmegen, The Netherlands.

b) Protinhi Therapeutics, Transistorweg 5, 6534 AT Nijmegen, The Netherlands.

c) Fraunhofer Institute for Cell Therapy and Immunology, Department of Infection Research and Diagnostics, Perlickstr. 1, 04103 Leipzig, Germany.

d) HermkensPharmaConsultancy B.V., Gripper 1, 5348 KZ Oss, The Netherlands.

Antiviral, lipopeptides, WNV, ZIKV, DENV, protease inhibitor

**Corresponding Authors**

* lorenzo.cavina@ru.nl

* martin.feiters@ru.nl

* floris.rutjes@ru.nl

# Supplementary Information

## Table of Contents

**Abbreviations** **S2**
**Experimental Procedures** **S2**
Chemistry S2
General Experimental S2
General Procedure for SPPS S2
General procedure A for mixed SPPS / in solution peptide synthesis S4
Synthesis and characterization of compounds S5
Biology S20
Biochemical Protease Assays S20
Cellular Viral Infection Assays S20
In vivo PK S23
**Supplementary Data** **S25**
Comparative view amongst the DENV2 antiviral cellular assays used in this work S25
Dose Response Curves (DRCs) S27
Library I S27
NS2B-NS3 inhibition S27
DENV2 cellular infection DRC S28
Viability S29
Library II S29
DENV2 NS2B-NS3 biochemical inhibition S29
DENV2 cellular infection S33
Viability S38
WNV and ZIKV NS2B-N3 inhibition S40
WNV and ZIKV cellular viral infection data S41
**Spectral Material** **S42**
LCMS traces S42
HPLC S43
NMR spectra S44
**AAALAC accreditation certificate** **S48**
**References** **S49**

**Abbreviations**

AAALAC Association for Assessment and Accreditation of Laboratory Animal Care; AcOH Acetic acid; Ala Alanine; API Atmospheric-pressure ionization; Arg Arginine; BHK Baby hamster kidney; Boc tert-butyloxycarbonyl; BSA Bovine serum albumin; BSL-2 Biosafety level 2; BSL-3 Biosafety level 3; C18 Octadecylsilyl silica; CAD Collision-activated dissociation; CDD Vault Collaborative Drug Discovery Vault; CE Collision energy; $CC_{50}$ 50% cytotoxic concentration; CPE Cytopathic effect; CTC Chlorotrityl chloride; CUR Curtain gas; CXP Collision cell exit potential; $D_2O$ Deuterium oxide; DCM Dichloromethane; DENV Dengue virus; DENV2 Dengue virus serotype 2; *N*,*N*-diisopropylethylamine ; *N*,*N'*-diisopropylcarbodiimide ; DMEM Dulbecco's

modified Eagle's medium; DMF N,N-dimethylformamide; DMSO-$d_6$ Deuterated dimethyl sulfoxide; DP Declustering potential; DRC Dose–response curve; $EC_{50}$ Half-maximal effective concentration; ELISpot Enzyme-linked immunospot; ESI Electrospray ionization; ESI-IT-MS Electrospray ionization ion-trap mass spectrometry; $Et_2O$ Diethyl ether; EtOAc Ethyl acetate; FCS Fetal calf serum; ffu Focus-forming units; Fmoc 9-fluorenylmethoxycarbonyl; Gln Glutamine; GS1 Nebulizer gas setting 1; GS2 Nebulizer gas setting 2; *O*-(7-azabenzotriazol-1-yl)-*N*,*N*,*N*′,*N*′-tetramethyluronium hexafluorophosphate ; His Histidine; HOBt 1-Hydroxybenzotriazole; HPLC High-performance liquid chromatography; HRMS High-resolution mass spectrometry; HRP Horseradish peroxidase; Hz Hertz; IgG Immunoglobulin G; IHE Instrument heater enable; ISV Ion spray voltage; J Coupling constant; LC-MS/MS Liquid chromatography–tandem mass spectrometry; LCMS Liquid chromatography–mass spectrometry; LLC-MK2 Rhesus monkey kidney cell line; Lys Lysine; MBHA 4-methylbenzhydrylamine; MeOH-$d_4$ Deuterated methanol; MeCN Acetonitrile; MeOH Methanol; MOI Multiplicity of infection; NCA Non-compartmental analysis; NMR Nuclear magnetic resonance; NS2B-NS3 Viral protease complex non-structural protein 2B and non-structural protein 3; Pbf 2,2,4,6,7-pentamethyldihydrobenzofuran-5-sulfonyl; PBS Phosphate-buffered saline; PDA Photodiode array; Phg Phenylglycine; Pip Pipecolic acid; PK Pharmacokinetics; PMS Phenazine methosulfate; ppm Parts per million; Pro Proline; RP-HPLC Reversed-phase high-performance liquid chromatography; RPMI1640 Roswell Park Memorial Institute medium 1640; RT Retention time; $R_t$ Retention time (chromatography); SPPS Solid-phase peptide synthesis; TEM Source temperature; TFA Trifluoroacetic acid; TFE 2,2,2-Trifluoroethanol; TIPS Triisopropylsilane; TMS Tetramethylsilane; Trt Triphenylmethyl (trityl); UPLC Ultra-performance liquid chromatography; UV Ultraviolet; Vero African green monkey kidney cell line; Vero E6 African green monkey kidney E6 subline; WNV West Nile virus; XTT 2,3-bis(2-methoxy-4-nitro-5-sulfophenyl)-5-[(phenylamino)carbonyl]-2H-tetrazolium hydroxide; ZIKV Zika virus;

# Experimental Procedures

## Chemistry

### General Experimental

Standard semi-automated SPPS was performed in empty open-top column cartridges with a plastic frit, which were agitated via an orbital shaker. All peptides were purified via **RP-HPLC** (unless stated otherwise) using a Shimadzu LC-20A Prominence system (Shimadzu, 's Hertogenbosch, The Netherlands) equipped with a C18 Gemini-NX column, 150 × 21.20 mm, particle size 10 µm (Phenomenex, Utrecht, The Netherlands), a pre-column guard and UV detection at 215 and 254 nm. RP-HPLC elution was performed with 0.1% TFA in a MeCN / milliQ $H_2O$ solution (isocratic 10% MeCN in $H_2O$ over 5 min, gradient from 10 to 100% MeCN in $H_2O$ over 20 min, with a solvent flow rate of 10.0 mL/min, unless stated otherwise). All final compounds are >95% pure by HPLC analysis. Chemicals were purchased from Fluorochem, VWR, Fischer Scientific or Sigma-Aldrich and used as received, unless stated otherwise. Reactions were magnetically stirred and carried out under inert atmosphere of dry nitrogen or argon, unless stated otherwise. Standard syringe techniques were applied for the transfer of dry solvents and air- or moisture-sensitive reagents. Reactions were followed via analytical LCMS. LCMS spectrograms were recorded on a Thermo Finnigan LCQ-Fleet ion trap mass spectrometer (ESI-IT-MS) coupled to a Shimadzu analytical HPLC [LC-20AD (pump) and SPD-M30A (photodiode array detector)], equipped with a Gemini C18 110A column, 50 mm××2 mm, particle size 3 µm (Phenomenex, Utrecht, The Netherlands), eluting with 0.1% formic acid in a MeOH / milliQ $H_2O$ solution (isocratic 5% MeOH in $H_2O$ over 5 min, gradient from 5 to 95% MeOH in $H_2O$ over 20 min, with a solvent flow rate of 1.0 mL/min). NMR spectra were recorded using either a Bruker Avance 400 (400 MHz) or a Bruker Avance III (500 MHz) spectrometer, in $D_2O$, MeOH-$d_4$, $CDCl_3$, or DMSO-$d_6$ solutions, unless stated otherwise. Chemical shifts are given in ppm with respect to residual non-deuterated solvents or TMS as internal standard for $CDCl_3$. Coupling constants are reported as J-values in Hz. The following abbreviations are used to explain multiplicities: s = singlet, d = doublet, t = triplet, q = quartet, dd = doublet of doublets, ddd = doublet of doublet of doublets, dtd = doublet of triplet of doublets, td = triplet of doublets, m = multiplet, br = broad signal. High resolution mass spectra (HRMS) were recorded on a JEOL AccuToF CS JMS-T100CS (ESI-HRMS). Regular MS (ESI-MS) measurements were recorded on Thermo Finnigan LCQ-Q Advantage Max.

### General Procedure for SPPS

***Fmoc deprotection.*** The resin was swollen with DCM (10 mL/gram of resin , 1 min) and DMF (2 × 10 mL/gram of resin, 1 min) and treated with 20% piperidine in DMF (10 mL/gram of resin) and left to shake for 20 min. The suspension was filtered and the resin was washed with DMF (2 × 10 mL/gram of resin, 1 min) and treated with a second portion of 20% piperidine in DMF (10 mL/gram of resin) and left to shake for 10 min. The suspension was then filtered and the resin was washed with DMF (3 × 10 mL/gram of resin, 1 min), DCM (3 × 10 mL/gram of resin, 1 min) and MeOH (3 × 10 mL/gram of resin, 1 min). Deprotection efficiency was determined by means of Kaiser or chloranil tests (for Pro deprotection).

***Loading of the first amino acid (Rink Amide MBHA resin).*** The resin was swollen with DCM (1 min) and DMF (2 × 1 min). Fmoc–amino acid (3 equiv.) and HOBt (3 equiv.) were dissolved in DMF (10 mL/gram of resin) and the resulting solution was added to the resin. Next DIPCDI (3 equiv.) was added and the reactor was left to shake for 16 h. The suspension was then filtered and the resin was washed with DMF (3 × 1 min), DCM (3 × 1 min), before treating with a

capping solution of acetic anhydride / pyridine (3 : 2, 10 mL/gram of resin) for 20 min The suspension was then filtered and the resin was washed with DMF (3 × 1 min), DCM (3 × 1 min) and MeOH (3 × 1 min). Coupling efficiency was determined by means of a Kaiser test.

***Loading of the first amino acid (CTC-Cl Barlos' resin).*** The Fmoc-amino acid (3 equiv.) was dissolved in dry DCM (10 mL/gram of resin) and collidine (3 equiv.), and the resulting solution was added to resin. The reactor was left shaking overnight, and the suspension was filtered before treating with a capping solution of 5% DIPEA in MeOH (10 mL/gram of resin) for 15 min The suspension was then filtered and the resin was washed with DMF (3 × 1 min), DCM (3 × 1 min) and MeOH (3 × 1 min).

***Peptide coupling.*** The resin was swollen with DCM (1 min) and DMF (2 × 1 min). Fmoc–amino acid or $R^1$-OH carboxylic acid (3 equiv.) and HOBt (3 equiv.) were dissolved in DMF (10 mL/gram of resin) and the resulting solution was added to the resin. Next DIPCDI (3 equiv.) was added and left to shake for 3 h. The suspension was then filtered and the resin was washed with DMF (3 × 1 min), DCM (3 × 1 min) and MeOH (3 × 1 min). Coupling efficiency was determined by means of a Kaiser or chloranil tests (for couplings on Pro).

***Coupling to carboxylic acid $R^1$-OH.*** The resin was swollen with DCM (1 min) and DMF (2 × 1 min). Palmitic acid (3 equiv.) and HATU (2.9 equiv.) were dissolved in DCM / DMF (1 : 1, 10 mL/gram of resin) and the resulting solution was added to the resin. Next DIPEA (3 equiv.) was added and the reactor was left to shake for 3 h. The suspension was then filtered and the resin was washed with DMF (3 × 1 min), DCM (3 × 1 min) and MeOH (3 × 1 min). Coupling efficiency was determined by means of Kaiser or chloranil tests (for couplings on Pro).

***Peptide cleavage (Rink Amide MBHA resin).*** The peptidyl-resin was washed with DCM (3 × 1 min) and dried under nitrogen. The resin was treated with a cleavage solution (95% TFA, 2.5% TIPS, 2.5% $H_2O$, 5 mL) and left to shake for 2 h (unless stated otherwise). The mixture was filtered and the resin was washed with DCM (3 × 1 min), filtrates were collected, combined and volatiles were removed *in vacuo*. The crude residue was triturated in dry $Et_2O$ and after centrifuge the precipitate was collected by decantation. Solvent leftovers were removed under high-vacuum.

***Peptide cleavage (CTC Barlos' resin).*** The peptidyl-resin was washed with DCM (3 × 1 min) and dried under nitrogen. The resin was treated with a cleavage solution (1 : 1 : 8 = AcOH : TFE : DCM, 10 mL / g of resin) and left to shake for 2 h (unless stated otherwise). The mixture was filtered and the resin was washed with further cleavage solution (1 : 1 : 8 = AcOH : TFE : DCM, 10 mL / g of resin) and with DCM (3 × 1 min). The filtrates were collected, combined and volatiles were removed *in vacuo*. The crude residue was triturated in dry $Et_2O$ and after centrifuge the precipitate was collected by decantation. Solvent leftovers were removed under high-vacuum. The crude materials were used as such in the next synthetic steps.

***Purification.*** The crude material was dissolved in minimal amount of MeOH (unless stated otherwise), filtered through a 0.20 µm syringe filter, and purified using preparative RP-HPLC (isocratic 20% MeCN in $H_2O$ over 5 min, gradient from 20 to 80% MeCN in $H_2O$ over 15 min, with a solvent flow rate of 10.0 mL/min, at 30 °C), unless stated otherwise. All fractions containing product were combined, concentrated to 5 mL *in vacuo*, prior to lyophilization to obtain the pure materials.

**General procedure A for mixed SPPS / in solution peptide synthesis**

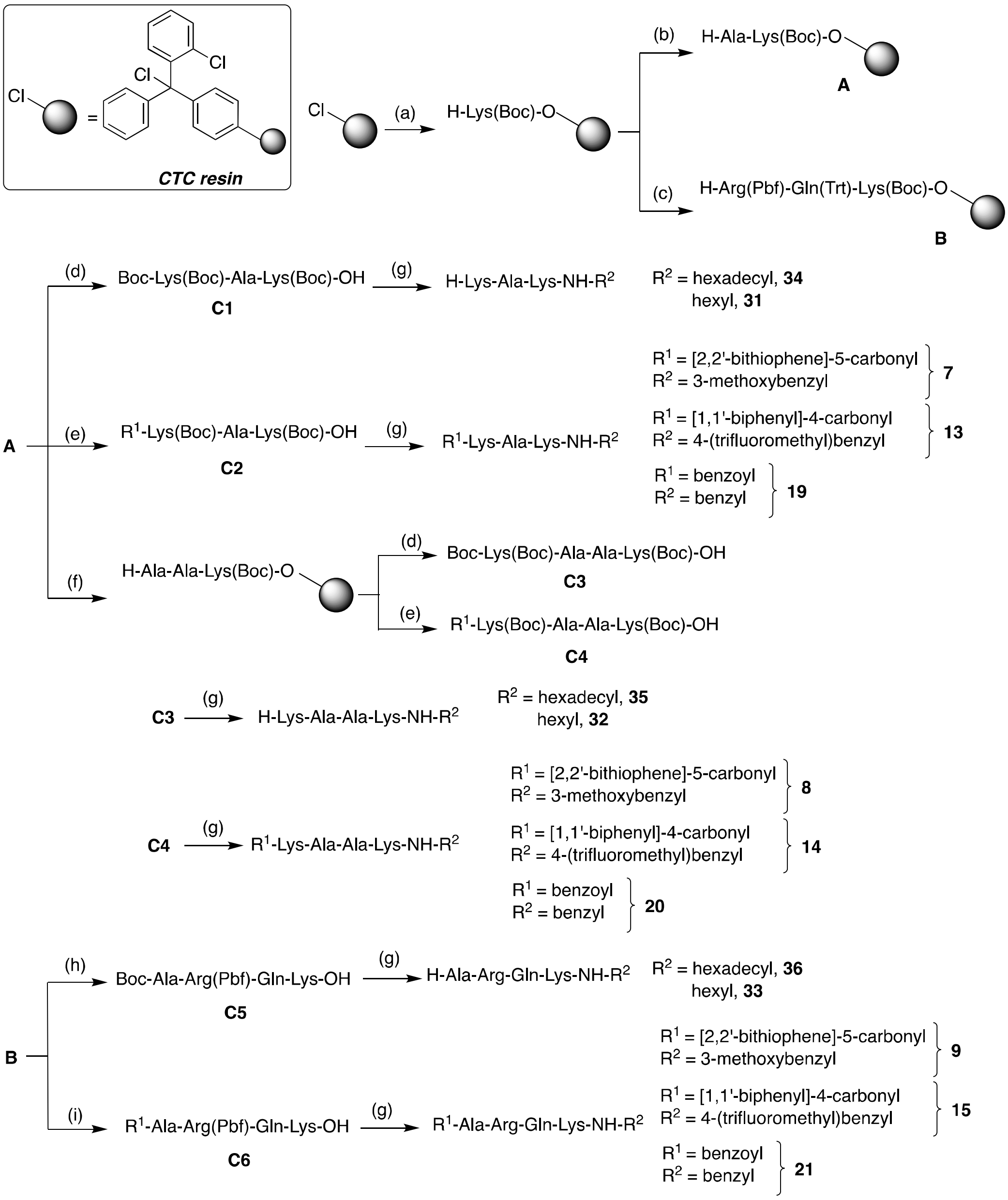


**Scheme S1. Synthetic scheme for mixed SPPS / in solution peptide synthesis.**| Reagents and conditions: (a) 1) Fmoc-Lys(Boc)-OH, DCM, collidine, 16 h; 2) MeOH, DIPEA, 0.25 h; 3) 20% piperidine in DMF, 0.5 h; (b) 1) Fmoc-Ala-OH, DIPCDI, HOBT, DMF, 3 h; 2) 20% piperidine in DMF, 0.5 h; (c) 1) Fmoc-Gln(Trt)-OH, DIPCDI, HOBT, DMF, 3 h; 2) 20% piperidine in DMF, 0.5 h; 3) Fmoc-Arg(Pbf)-OH, DIPCDI, HOBT, DMF, 3 h; 4) 20% piperidine in DMF, 0.5 h; (d) 1) Boc-Lys(Boc)-OH, DIPCDI, HOBT, DMF, 3 h; 2) AcOH / TFE / DCM (1 : 1 : 8), 2h; (e) 1) Fmoc-Lys(Boc)-OH, DIPCDI, HOBT, DMF, 3 h; 2) 20% piperidine in DMF, 0.5 h; 3) $R^1$-OH, HATU, DIPEA, DMF, 3 h; 4) AcOH / TFE / DCM (1 : 1 : 8), 2h; (f) 1) Fmoc-Ala-OH, DIPCDI, HOBT, DMF, 3 h; 2) 20% piperidine in DMF, 0.5 h; (g) 1) Amine $R^2$-$NH^2$, HATU, DIPEA, DMF, 3 h; 2) H2O / TIPS / TFA (2.5 : 2.5 : 95), 1.5 h; (h) 1) Boc-Ala-OH, DIPCDI, HOBT, DMF, 3 h; 4) AcOH / TFE / DCM (1 : 1 : 8), 2h; (i) 1) Fmoc-Ala-OH, DIPCDI, HOBT, DMF, 3 h; 2) 20% piperidine in DMF, 0.5 h; 3) Carboxylic acid $R^1$-OH, HATU, DIPEA, DMF, 3 h; 4) AcOH / TFE / DCM (1 : 1 : 8), 2h;

Side-chain protected peptide carboxylic acids **C1-6** were synthesized according to the general procedure for SPPS using CTC-Cl Barlos' resin. To a DMF or DCM solution (0.1 M) of crude peptide carboxylic acids **C1-6** (1.0 equiv.), amine **$R^2$-$NH_2$** (1.5 equiv.) was added at 0 °C, followed by HATU (1.2 equiv.) and DIPEA (3.0 equiv.). The resulting mixture was warmed to room temperature and stirred for 3 h, before it was quenched by diluting with water (10 volumes of the reaction solvent). The aqueous layer was extracted with EtOAc (3 × 5 volumes of the reaction solvent) and the combined organic layer was washed with 5% aqueous $NaHCO_3$ (3 × 5 volumes of the reaction solvent), before it was dried over

$Na_2SO_4$ and it was concentrated *in vacuo*. The crude residue was dissolved in TFA / TIPS / water (95 : 2.5 : 2.5, 1.0 M) and the resulting mixture was stirred for 1.5 h, before it was concentrated *in vacuo*. The crude residue was triturated in cold $Et_2O$ (3 × 10 volumes of the reaction solvent), the solid was decanted and it was purified via RP-HPLC. Fractions containing the purified peptide (>95% purity by LCMS) were combined, concentrated to half of the original volume and lyophilized, to recover the final compounds as TFA salt.

**Synthesis and characterization of compounds**

**[2,2'-Bithiophene]-5-carbonyl-Lys-Ala-Lys-(3-methoxy)benzylamide bisTFA (7)** was synthesized according to general procedure A, using **[2,2'-bithiophene]-5-carboxylic acid** as $R^1$-OH and **(3-methoxy)benzyl amine** as $R^2$-$NH_2$. After purification via RP-HPLC, compound **7** (41 mg) was obtained as a white powder. $^1H$ NMR (400 MHz, DMSO-$d_6$): δ 7.83 – 7.82 (m, 1H), 7.59 (d, $J$ = 5.2 Hz, 1H), 7.42 (d, $J$ = 3.6 Hz, 1H), 7.34 (d, $J$ = 3.6 Hz, 1H), 7.25 – 7.19 (m, 1H), 7.18 – 7.11 (m, 1H), 6.82 – 6.78 (m, 3H), 4.40 – 4.35 (m, 1H), 4.60 -- 4.38 (m, 4H), 3.78 – 3.70 (m, 3H), 2.74 (dt, $J$ = 7.2, 11.6 Hz, 1H), 1.85 – 1.22 (m, 18H). LCMS (ESI): *m/z* calcd for $C_{32}H_{45}N_6O_5S_2^+$ [M + H]$^+$, 657.29; found 657.34.

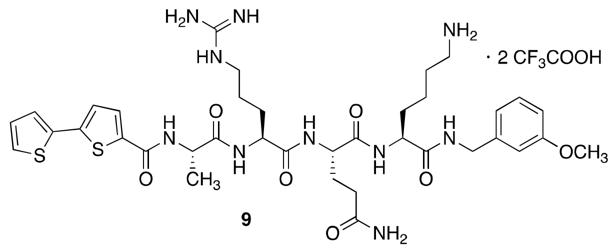

**9**

**(2,2'-Bithiophene)-5-carbonyl-Lys-Ala-Ala-Lys-NH-3-(methoxy)benzyl bisTFA (8)** was synthesized according to general procedure A, using **[2,2'-bithiophene]-5-carboxylic acid** as $R^1$-OH and **(3-methoxy)benzyl amine** as $R^2$-$NH_2$. After purification via RP-HPLC, compound **8** (58 mg) was obtained as a white powder. $^1H$ NMR (400 MHz, DMSO-$d_6$): δ 8.60 (d, $J$ = 8.0 Hz, 1H), 8.45 – 8.38 (m, 1H), 8.15 (d, $J$ = 7.6 Hz, 1H), 8.02 (d, $J$ = 7.2 Hz, 1H), 7.94 – 7.84 (m, 2H), 7.67 (br, 5H), 7.61 (d, $J$ = 5.6 Hz, 1H), 7.43 (d, $J$ = 4.0 Hz, 1H), 7.35 (d, $J$ = 3.6 Hz, 1H), 7.25 – 7.18 (m, 1H), 7.15 – 7.12 (m, 1H), 6.84 – 6.76 (m, 3H), 4.40 – 4.35 (m, 1H), 4.32 – 4.18 (m, 5H), 3.73 (s, 3H), 2.82 – 2.71 (m, 4H), 1.85 – 1.18 (m, 18H). LCMS (ESI): *m/z* calcd for $C_{35}H_{49}N_7O_6S_2^+$ [M + H]$^+$, 728.33; found 728.51.

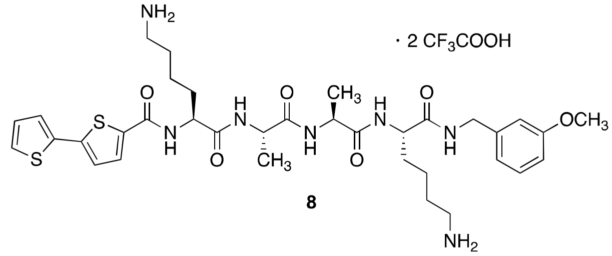

**8**

**[2,2'-Bithiophene]-5-carbonyl-Ala-Arg-Gln-Lys-3-(methoxy)benzylamide bisTFA (9)** was synthesized according to general procedure A, using **[2,2'-bithiophene]-5-carboxylic acid** as $R^1$-OH and **(3-methoxy)benzyl amine** as $R^2$-$NH_2$. After purification via RP-HPLC, compound **9** (43 mg) was obtained as a white powder. $^1H$ NMR (400 MHz, DMSO-$d_6$): δ 8.95 (br, 1H), 8.48 – 8.21 (m, 4H), 8.13 (d, $J$ = 8.0 Hz, 1H), 7.94 (d, $J$ = 3.6 Hz), 7.73 – 7.49 (m, 3H), 7.48 – 7.16 (m, 6H), 7.12 (dd, $J$ = 4.0, 4.8 Hz, 1H), 6.75 – 6.68 (m, 4H), 4.50 – 4.39 (m, 1H), 4.35 – 4.10 (m, 5H), 3.72 (s, 3H), 3.15 – 2.95 (m, 4H), 2.75 – 2.65 (m, 2H), 2/22 – 2.0 (m, 2H), 1.99 – 1.43 (m, 10H), 1.42 – 1.20 (m, 5H). LCMS (ESI): *m/z* calcd for $C_{37}H_{53}N_{10}O_7S_2^+$ [M + H]$^+$, 813.35; found 813.61.

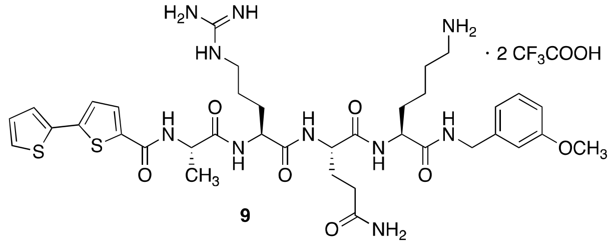

**9**

**[2,2'-Bithiophene]-5-carbonyl-Lys-Ala-Lys-$NH_2$ bisTFA (10)** was synthesized according to the general procedure for SPPS, using MBHA Rink amide resin and **[2,2'-bithiophene]-5-carboxylic acid** as $R^1$-OH. After purification via RP-HPLC, compound **10** (39 mg) was obtained as a white powder. $^1H$ NMR (400 MHz, DMSO-$d_6$): δ 8.62 (d, $J$ = 10.8 Hz, 1H), 8.17 (d, $J$ = 9.6 Hz, 1H), 7.88 (d, $J$ = 4.8 Hz, 1H), 7.82 (d, $J$ = 10.8 Hz, 1H), 7.72 (br, 5H), 7.63 – 7.58 (m, 1H), 7.45 – 7.41 (m, 1H), 7.35 (d, $J$ = 4.8 Hz, 1H), 7.30 (br, 1H), 7.13 (dd, $J$ = 4.8, 6.8 Hz, 1H), 7.06 (br, 1H), 4.45 – 4.34 (m, 1H), 4.32 – 4.20 (m, 1H), 4.18 – 4.08 (m, 1H), 2.85 – 2.68 (m, 4H), 1.86 – 1.18 (m, 15H). LCMS (ESI): *m/z* calcd for $C_{24}H_{37}N_6O_4S_2^+$ [M + H]$^+$, 537.23; found 537.32.

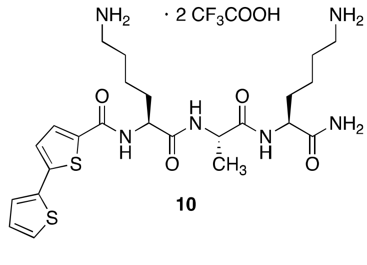

**10**

**(2,2'-Bithiophene)-5-carbonyl-Lys-Ala-Ala-Lys-NH2 bisTFA (11)** was synthesized according to the general procedure for SPPS, using MBHA Rink amide resin and **[2,2'-bithiophene]-5-carboxylic acid** as $R^1$-OH. After purification via RP-HPLC, compound **11** (14 mg) was obtained as a white powder. $^1$H NMR (400 MHz, DMSO-$d_6$): δ 8.58 (d, $J$ = 10.8 Hz, 1H), 8.17 (d, $J$ = 9.6 Hz, 1H), 8.01 (d, $J$ = 9.6 Hz, 1H), 7.87 (d, $J$ = 5.6 Hz, 1H), 7.82 – 7.47 (m, 5H), 7.45 – 7.41 (m, 1H), 7.38 – 7.28 (m, 2H), 7.18 – 7.08 (m, 2H), 4.48 – 4.10 (m, 4H), 2.84 – 2.70 (m, 4H), 1.80 – 1.10 (m, 18H). LCMS (ESI): $m/z$ calcd for $C_{27}H_{42}N_7O_5S_2^+$ $[M + H]^+$, 608.27; found 608.49.

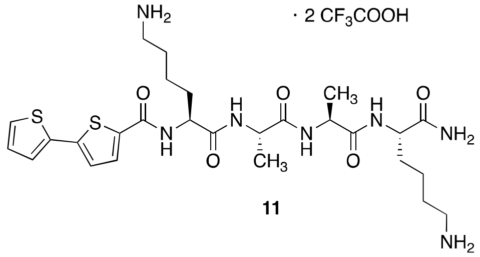

**[2,2'-Bithiophene]-5-carbonyl-Ala-Arg-Gln-Lys-NH2 bisTFA (12)** was synthesized according to the general procedure for SPPS, using MBHA Rink amide resin and **[2,2'-bithiophene]-5-carboxylic acid** as $R^1$-OH. After purification via RP-HPLC, compound **12** (45 mg) was obtained as a white powder. $^1$H NMR (400 MHz, DMSO-$d_6$): δ 8.65 (d, $J$ = 7.2 Hz, 1H), 8.18 (d, $J$ = 7.2 Hz, 1H), 7.97 (d, $J$ = 7.6 Hz, 1H), 7.90 – 7.80 (m, 2H), 7.72 (br, 3H), 7.63 – 7.50 (m, 2H), 7.47 – 7.38 (m, 1H), 7.37 – 7.19 (m, 4H), 7.19 – 6.90 (m, 4H), 6.76 (s, 1H), 4.55 – 4.39 (m, 1H), 4.32 – 4.05 (m, 3H), 3.29 – 3.04 (m, 2H), 2.85 – 2.71 (m, 2H), 2.20 – 2.06 (m, 2H), 2.00 – 1.87 (m, 1H), 1.85 – 1.63 (m, 3H), 1.62 – 1.43 (m, 6H), 1.42 – 1.18 (m, 5H). LCMS (ESI): $m/z$ calcd for $C_{29}H_{45}N_{10}O_6S_2^+$ $[M + H]^+$, 693.30; found 693.52.

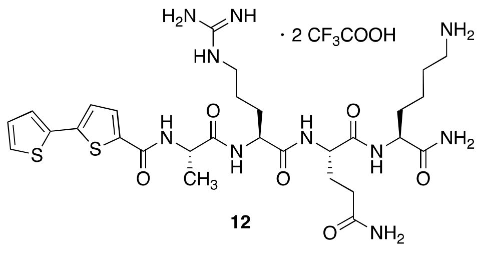

**[1,1'-Biphenyl]-4-carbonyl-Lys-Ala-Lys-4-(trifluoromethyl)benzylamide bisTFA (13)** was synthesized according to general procedure A, using **[1,1'-biphenyl]-4-carboxylic acid** as $R^1$-OH and **4-(trifluoromethyl)benzyl amine** as $R^2$-$NH_2$. After purification via RP-HPLC, compound **13** (13 mg) was obtained as a white powder. $^1$H NMR (400 MHz, DMSO-$d_6$): δ 8.55 (d, $J$ = 7.6 Hz, 1H), 8.47 (t, $J$ = 6.4 Hz, 1H), 8.08 (d, $J$ = 6.8 Hz, 1H), 8.05 – 7.97 (m, 3H), 7.81 – 7.67 (m, 11 H), 7.52 – 7.39 (m, 5H), 4.50 – 4.17 (m, 5H), 2.77 (dt, $J$ = 6.0, 16.4 Hz, 4H), 1.85 – 1.21 (m, 15H). LCMS (ESI): $m/z$ calcd for $C_{36}H_{46}F_3N_6O_4^+$ $[M + H]^+$, 683.35; found 683.55.

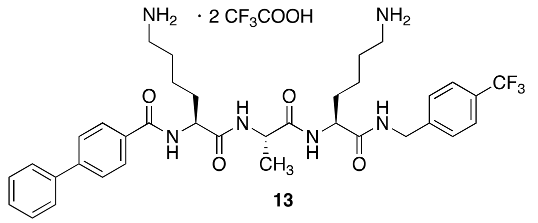

**(1,1'-Biphenyl)-4-carbonyl-Lys-Ala-Ala-Lys-NH-4-(trifluoromethyl)benzyl bisTFA (14)** was synthesized according to the general procedure for SPPS using MBHA Rink amide resin and **[1,1'-biphenyl]-4-carboxylic acid** as $R^1$-OH. After purification via RP-HPLC, compound **14** (16 mg) was obtained as a white powder. $^1$H NMR (400 MHz, DMSO-$d_6$): δ 8.59 – 8.48 (m, 2H), 8.14 – 7.98 (m, 5H), 7.84 – 7.58 (m, 10H), 7.54 – 7.40 (m, 5H), 4.50 – 4.18 (m, 6H), 2.85 – 2.68 (m, 4H), 1.85 – 1.15 (m, 18H). LCMS (ESI): $m/z$ calcd for $C_{39}H_{51}F_3N_7O_5^+$ $[M + H]^+$, 754.39; found 754.66.

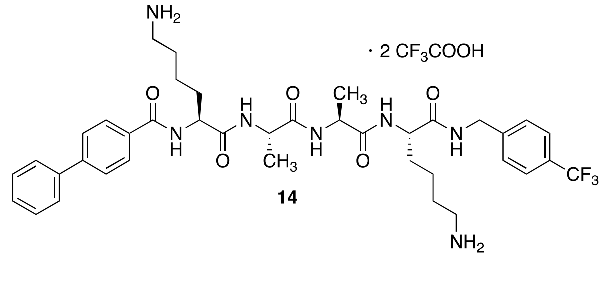

**[1,1'-Biphenyl]-4-carbonyl-Ala-Arg-Gln-Lys-NH-4-(trifluoromethyl)benzyl bisTFA (15)** was synthesized according to general procedure A, using **[1,1'-biphenyl]-4-carboxylic acid** as $R^1$-OH and **4-(trifluoromethyl)benzyl amine** as $R^2$-$NH_2$. After purification via RP-HPLC, compound **15** (36 mg) was obtained as a white powder. $^1$H NMR (400 MHz, DMSO-$d_6$): δ 8.62 (d, $J$ = 6.8 Hz, 1H), 8.51 (t, $J$ = 6.0 Hz, 1H), 8.15 (d, $J$ = 7.6 Hz, 1H), 8.18 – 7.94 (m, 4H), 7.84 – 7.63 (m, 8H), 7.56 (br, 1H), 7.53 – 7.38 (m, 5H), 7.25 (s, 1H), 6.78 (s, 1H), 4.53 – 4.47 (m, 1H), 4.44 – 4.31 (m, 2H), 4.30 – 4.19 (m, 3H), 3.18 – 3.04 (m, 2H), 2.80 – 2.70 (m, 2H), 2.19 – 2.06 (m, 2H), 2.03 – 1.86 (m, 1H), 1.85 – 1.65 (m, 3H), 1.64. – 1.46 (m, 6H), 1.43 – 1.20 (m, 5H). LCMS (ESI): $m/z$ calcd for $C_{41}H_{54}F_3N_{10}O_6^+$ $[M + H]^+$, 839.42; found 839.62.

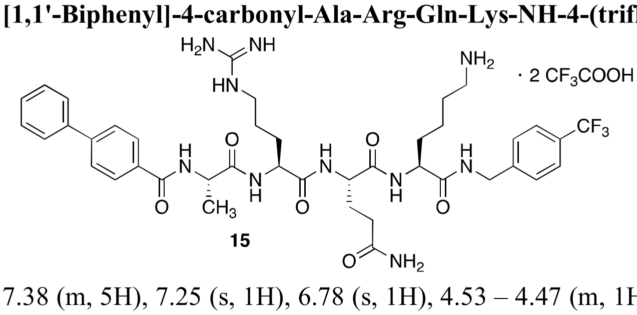

**[1,1'-Biphenyl]-4-carbonyl-Lys-Ala-Lys-$NH_2$ bisTFA (16)** was synthesized according to the general procedure for SPPS using MBHA Rink amide resin and **[1,1'-biphenyl]-4-carboxylic acid** as $R^1$-OH. After purification via RP-HPLC, compound **16** (33 mg) was obtained as a white powder. $^1$H NMR (400 MHz, DMSO-$d_6$): δ 8.55 (d, $J$ = 8.0 Hz, 1H), 8.07 (d, $J$ = 7.6 Hz, 1H), 8.03 – 7.96 (m, 2H), 8.85 – 7.56 (m, 10H), 7.54 – 7.47 (m, 2H), 7.46 – 7.39 (m, 1H), 7.25 (s, 1H), 7.02 (s, 1H), 4.49 – 4.39 (m, 1H), 4.28 (dt, $J$ = 7.6, 14.4 Hz, 1H), 4.13 (dt, $J$ = 3.2, 11.2 Hz, 1H), 2.85 – 2.68 (m, 4H), 2.17 – 2.08 (m, 2H), 1.87 – 1.18 (m, 15H). LCMS (ESI): $m/z$ calcd for $C_{28}H_{41}N_6O_4^+$ $[M + H]^+$, 525.32; found 525.44.

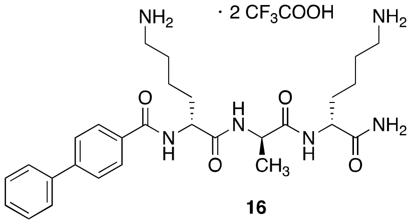

**16**

**(1,1'-Biphenyl)-4-carbonyl-Lys-Ala-Ala-Lys-$NH_2$ bisTFA (17)** was synthesized according to the general procedure for SPPS, using MBHA Rink amide resin and **[1,1'-biphenyl]-4-carboxylic acid** as $R^1$-OH. After purification via RP-HPLC, compound **17** (19 mg) was obtained as a white powder. $^1$H NMR (400 MHz, DMSO-$d_6$): δ 8.58 – 8.52 (m, 1H), 8.12 (d, $J$=10.0 Hz, 1H), 7.88 – 7.68 (m, 6H), 7.64 – 7.38 (m, 6H), 7.31 (s, 1H), 7.08 (s, 1H), 4.50 – 4.10 (m, 5H), 2.83 – 2.71 (m, 3H), 1.97 – 1.18 (m, 18H). LCMS (ESI): $m/z$ calcd for $C_{31}H_{46}N_7O_5^+$ $[M + H]^+$, 596.36; found 596.54.

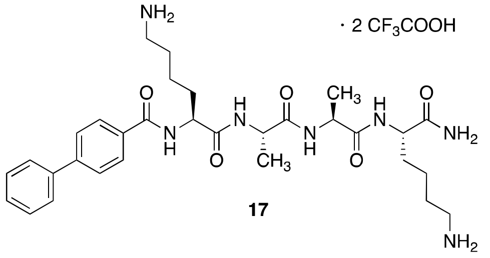

**17**

**[1,1'-Biphenyl]-4-carbonyl-Ala-Arg-Gln-Lys-$NH_2$ bisTFA (18)** was synthesized according to the general procedure for SPPS, using MBHA Rink amide resin and **[1,1'-biphenyl]-4-carboxylic acid** as $R^1$-OH. After purification via RP-HPLC, compound **18** (27 mg) was obtained as a white powder. $^1$H NMR (400 MHz, DMSO-$d_6$): δ 8.65 (d, $J$ = 8.8 Hz, 1H), 8.18 (d, $J$ = 10.0 Hz, 1H), 8.46 – 7.93 (m, 3H), 7.93 – 7.66 (m, 1H), 7.85 – 7.65 (m, 6H), 7.59 – 7.47 (m, 3H), 7.46 – 7.38 (m, 1H), 7.37 – 7.19 (m, 3H), 7.09 (s, 1H), 6.96 – 6.46 (m, 3H), 4.55 – 4.41 (m, 1H), 4.30 – 4.10 (m, 3H), 3.18 – 3.05 (m, 2H), 2.86 – 2.67 (m, 2H), 2.20 – 2.05 (m, 2H), 2.04 – 1.88 (m, 1H), 1.87 – 1.45 (m, 9H), 1.45 –1.23 (m, 5H). LCMS (ESI): $m/z$ calcd for $C_{33}H_{49}N_{10}O_6^+$ $[M + H]^+$, 681.38; found 681.51.

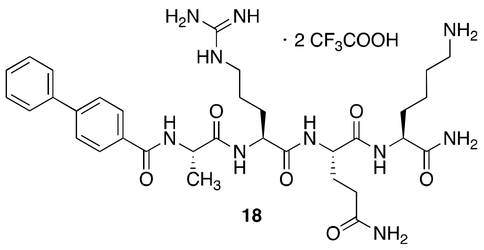

**18**

**Benzoyl-Lys-Ala-Lys-benzylamide bisTFA (19)** was synthesized according to general procedure A, using **benzoic acid** as $R^1$-OH and **benzyl amine** as $R^2$-$NH_2$. After purification via RP-HPLC, compound **19** (55 mg) was obtained as a white powder. $^1$H NMR (400 MHz, DMSO-$d_6$): δ 8.47 (d, $J$ = 7.6 Hz, 1H), 8.38 – 8.30 (m, 1H), 8.17 – 7.87 (m, 4H), 7.59 – 7.45 (m, 4H), 7.34 – 7.28 (m, 2H), 7.26 – 7.20 (m, 3H), 4.48 – 4.78 (m, 1H), 4.66 – 4.38 (m, 4H), 2.71 (dt, $J$ = 7.2, 19.6 Hz, 4H), 1.85 – 1.20 (m, 16H). LCMS (ESI): $m/z$ calcd for $C_{29}H_{43}N_6O_4^+$ $[M + H]^+$, 539.33; found 539.50.

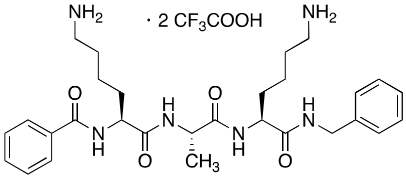

**19**

**Benzoyl Lys-Ala-Ala-Lys-NH-benzyl bisTFA (20)** was synthesized according to general procedure A, using **benzoic acid** as $R^1$-OH and **benzyl amine** as $R^2$-$NH_2$. After purification via RP-HPLC, compound **20** (40 mg) was obtained as a white powder. $^1$H NMR (400 MHz, DMSO-$d_6$): δ 8.52 – 8.36 (m, 2H), 8.08 (d, $J$ = 9.6 Hz, 1H), 8.2 (d, $J$ = 9.6 Hz, 1H), 7.94 – 7.84 (m, 3H), 7.66 (br, 5H), 7.59 – 7.44 (m, 3H), 7.37– 7.20 (m, 5H) 4.50 – 4.18 (m, 6H), 2.85 – 2.68 (m, 4H), 1.85 – 1.15 (m, 18H). LCMS (ESI): $m/z$ calcd for $C_{32}H_{48}N_7O_5^+$ $[M + H]^+$, 610.37; found 610.60.

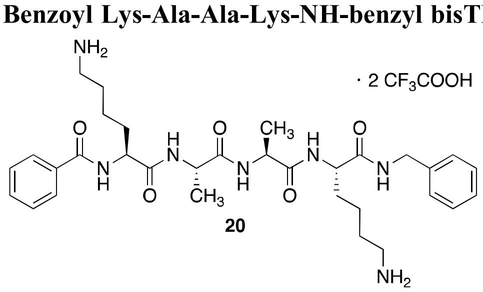

**20**

**Benzoyl-Ala-Arg-Gln-Lys-NH-benzyl bisTFA (21)** was synthesized according to general procedure A, using **benzoic acid** as $R^1$-OH and **benzyl amine** as $R^2$-$NH_2$. After purification via RP-HPLC, compound **21** (55 mg) was obtained as a white powder. $^1$H NMR (400 MHz, DMSO-$d_6$): δ 8.54 (d, $J$ = 6.4 Hz, 1H), 8.42 – 8.34 (m, 1H), 8.12 (d, $J$ = 8.0 Hz, 1H), 8.00 (d, $J$ = 8.4 Hz, 1H), 7.96 – 7.86 (m, 3H), 7.70 – 7.59 (m, 2H), 7.58 – 7.51 (m, 1H), 7.57 – 7.44 (m, 3H), 7.34 – 7.28 (m, 2H), 7.26 – 7.18 (m, 3H), 7.17 – 6.80 (m, 3H), 6.78 (s, 1H), 4.50 – 4.40 (m, 1H), 4.37 – 4.15 (m, 5H), 3.15 – 3.05 (m, 2H), 2.80 – 2.68 (2H), 2.15 – 2.07 (m, 2H), 2.00 – 1.90 (m, 1H), 1.85 – 1.65 (m, 3H), 1.64 – 1.42 (6H), 1.40 – 1.20 (m, 5H). LCMS (ESI): $m/z$ calcd for $C_{34}H_{51}N_{10}O_6^+$ [M + H]$^+$, 695.40; found 695.50.

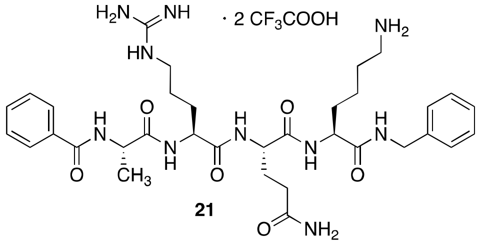

**21**

**Benzoyl-Lys-Ala-Lys-NH₂ bisTFA (22)** was synthesized according to the general procedure for SPPS, using MBHA Rink amide resin and **benzoic acid** as $R^1$-OH. After purification via RP-HPLC, compound **22** (13 mg) was obtained as a white powder. $^1$H NMR (400 MHz, DMSO-$d_6$): δ 8.48 (d, $J$ = 7.6 Hz, 1H), 8.05 (d, $J$ = 7.2 Hz, 1H), 7.92 – 7.86 (m, 2H), 7.80 (d, $J$ = 8.4 Hz, 1H), 7.77 – 7.44 (m, 8H), 7.25 (s, 1H), 7.01 (s, 1H), 4.45 – 4.34 (m, 1H), 4.27 (dt, $J$ = 7.2, 14.0 Hz, 1H), 4.18 – 4.08 (m, 1H), 2.85 – 2.68 (m, 4H), 1.86 – 1.18 (m, 15H). LCMS (ESI): $m/z$ calcd for $C_{22}H_{37}N_6O_4^+$ [M + H]$^+$, 449.29; found 449.33.

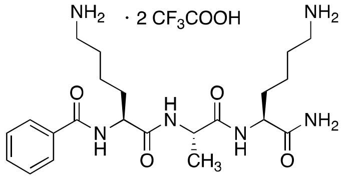

**22**

**Benzoyl-Lys-Ala-Ala-Lys-NH₂ bisTFA (23)** was synthesized according to the general procedure for SPPS, using MBHA Rink amide resin and **benzoic acid** as $R^1$-OH. After purification via RP-HPLC, compound **23** (95 mg) was obtained as a white powder. $^1$H NMR (400 MHz, DMSO-$d_6$): δ 8.49 (d, $J$ = 10.0 Hz, 1H), 8.12 (d, $J$ = 9.2 Hz, 1H), 8.01 (d, $J$ = 9.2 Hz, 1H), 7.74 – 7.88 (m, 2H), 7.87 – 7.60 (m, 7H), 7.58 – 7.44 (m, 3H), 7.31 (s, 1H), 7.07 (m, 1H), 4.50 – 4.36 (m, 1H), 4.35 – 4.09 (m, 3H), 2.84 – 2.68 (m, 4H), 1.86 – 1.13 (m, 18H). LCMS (ESI): $m/z$ calcd for $C_{25}H_{42}N_7O_5^+$ [M + H]$^+$, 520.32; found 520.42.

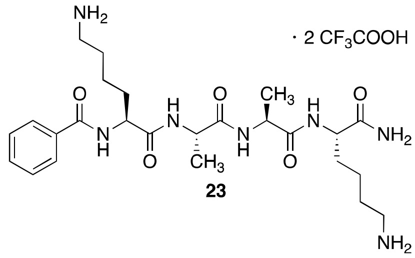

**23**

**Benzoyl-Ala-Arg-Gln-Lys-NH₂ bisTFA (24)** was synthesized according to the general procedure for SPPS, using MBHA Rink amide resin and **benzoic acid** as $R^1$-OH. After purification via RP-HPLC, compound **24** (26 mg) was obtained as a white powder. $^1$H NMR (400 MHz, DMSO-$d_6$): δ 8.65 (d, $J$ = 7.2 Hz, 1H), 8.18 (d, $J$ = 7.2 Hz, 1H), 7.97 (d, $J$ = 7.6 Hz, 1H), 7.90 – 7.80 (m, 2H), 7.72 (br, 3H), 7.63 – 7.50 (m, 2H), 7.47 – 7.38 (m, 1H), 7.37 – 7.19 (m, 4H), 7.19 – 6.90 (m, 4H), 6.76 (s, 1H), 4.55 – 4.39 (m, 1H), 4.32 – 4.05 (m, 3H), 3.29 – 3.04 (m, 2H), 2.85 – 2.71 (m, 2H), 2.20 – 2.06 (m, 2H), 2.00 – 1.87 (m, 1H), 1.85 – 1.63 (m, 3H), 1.62 – 1.43 (m, 6H), 1.42 – 1.18 (m, 5H). LCMS (ESI): $m/z$ calcd for $C_{29}H_{45}N_{10}O_6S_2^+$ [M + H]$^+$, 605.30; found 605.45.

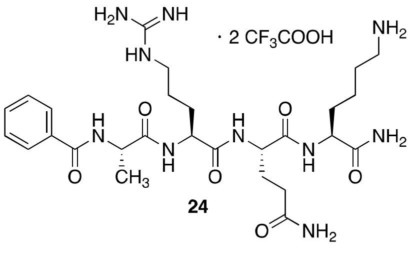

**24**

**Heptanoyl-Lys-Ala-Lys-NH-*n*-hexyl bisTFA (25)** was synthesized according to general procedure A, using **heptanoic acid** as $R^1$-OH and **hexyl amine** as $R^2$-$NH_2$. After purification via RP-HPLC, compound **25** (68 mg) was obtained as a white powder. $^1$H NMR (400 MHz, DMSO-$d_6$): δ 7.95 (d, $J$ = 7.6 Hz, 1H), 7.90 (d, $J$ = 6.8 Hz, 1H), 7.81 (d, $J$ = 8.4 Hz, 1H), 7.74 (t, $J$ = 5.2 Hz, 1H), 7.64 (br, 5H), 4.35 – 4.10 (m, 3H), 3.10 – 2.95 (m, 2H), 2.80 – 2.70 (m, 4H), 2.17 – 2.08 (m, 2H), 1.70 – 1.58 (m, 2H), 1.54 – 1.42 (m, 8H), 1.41 – 1.16 (m, 21H), 0.86 (t, $J$ = 6.4 Hz, 6H). LCMS (ESI): $m/z$ calcd for $C_{28}H_{57}N_6O_4^+$ [M + H]$^+$, 541.44; found 541.55.

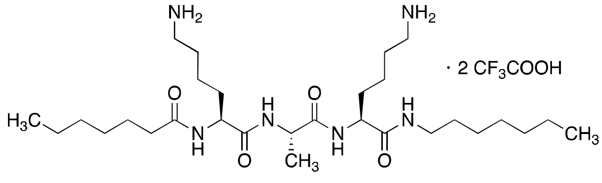

**25**

**Heptanoyl-Lys-Ala-Ala-Lys-NH-*n*-hexyl bisTFA (26)** was synthesized according to general procedure A, using **heptanoic acid** as $R^1$-OH and ***n*-hexadecyl amine** as $R^2$-$NH_2$. After purification via RP-HPLC, compound **26** (24 mg) was obtained as a white powder. $^1$H NMR (400 MHz, DMSO-$d_6$): δ 7.98 – 7.92 (m, 3H), 7.88 – 7.50 (m, 8H), 4.30 – 4.10 (m, 4H), 3.08 – 2.98 (m, 2H), 2.78 – 2.68 (m, 4H), 2.17 – 2.08 (m, 2H), 1.72 – 1.57 (m, 2H), 1.56 – 1.43 (m, 8H), 1.42 – 1.12 (m, 24H), 0.86 (t, *J* = 6.4 Hz, 6H). LCMS (ESI): *m/z* calcd for $C_{31}H_{62}N_7O_5^+$ [M + H]$^+$, 612.48; found 612.65.

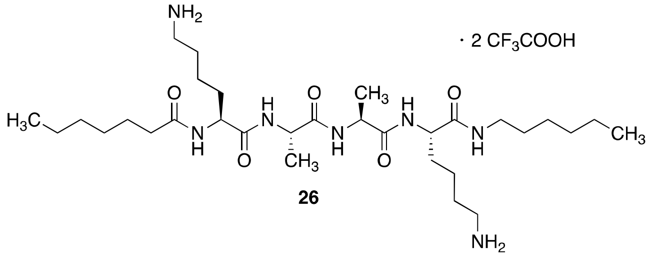

**26**

**Heptanoyl-Ala-Arg-Gln-Lys-NH-*n*-hexyl bisTFA (27)** was synthesized according to general procedure A, using **heptanoic acid** as $R^1$-OH and ***n*-hexyl amine** as $R^2$-$NH_2$. After purification via RP-HPLC, compound **27** (68 mg) was obtained as a white powder. $^1$H NMR (400 MHz, DMSO-$d_6$): δ 8.07 – 7.95 (m, 2H), 7.94 – 7.83 (m, 2H), 7.82 – 7.72 (m, 1H), 7.72 – 7.42 (m, 4H), 7.23 (s, 1H), 7.20 – 6.80 (m, 3H), 6.77 (s, 1H), 4.40 – 4.0 (m, 4H), 3.20 – 2.90 (m, 5H), 2.84 – 2.66 (m, 2H), 2.22 – 1.99 (m, 4H), 1.98 – 1.83 (m, 1H), 1.82 – 1.34 (14 H), 1.34 – 1.09 (m, 17H), 0.85 (t, *J* = 6.8 Hz, 6H). LCMS (ESI): *m/z* calcd for $C_{33}H_{65}N_{10}O_6^+$ [M + H]$^+$, 697.51; found 697.68.

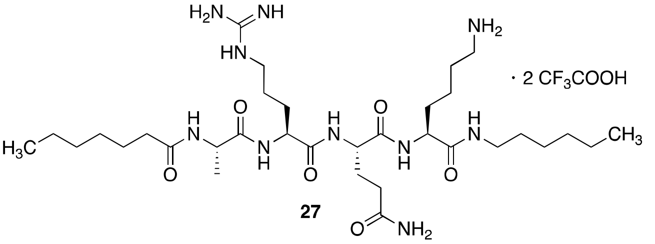

**27**

**Heptanoyl-Lys-Ala-Lys-NH₂ bisTFA (28)** was synthesized according to the general procedure for SPPS, using MBHA Rink amide resin and **heptanoic acid** as $R^1$-OH. After purification via RP-HPLC, compound **28** (13 mg) was obtained as a white powder. $^1$H NMR (400 MHz, DMSO-$d_6$): δ 7.98 – 7.92 (m, 2H), 7.77 (d, *J* = 8.4 Hz, 1H), 7.68 (br, 6H), 7.24 (s, 1H), 7.02 (s, 1H), 4.36 – 4.07 (m, 3H), 2.83 – 2.70 (m, 4H), 2.17 – 2.08 (m, 2H), 1.73 – 1.60 (m, 2H), 1.58 – 1.42 (m, 8H), 1.40 – 1.17 (m, 13H), 0.86 (t, *J* = 6.4 Hz, 3H). LCMS (ESI): *m/z* calcd for $C_{22}H_{45}N_6O_4^+$ [M + H]$^+$, 457.35; found 457.38.

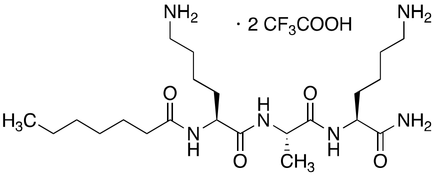

**28**

**Heptanoyl-Lys-Ala-Ala-Lys-NH₂ bisTFA (29)** was synthesized according to the general procedure for SPPS, using MBHA Rink amide resin and **heptanoic acid** as $R^1$-OH. After purification via RP-HPLC, compound **29** (11 mg) was obtained as a white powder. LCMS (ESI): *m/z* calcd for $C_{25}H_{50}N_7O_5^+$ [M + H]$^+$, 528.4; found 528.4.

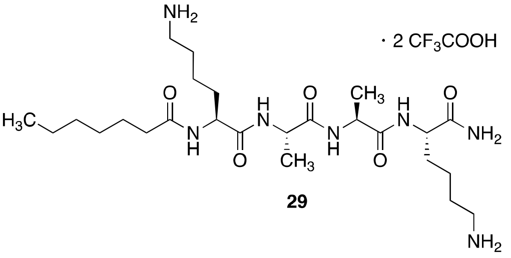

**29**

**Heptanoyl-Ala-Arg-Gln-Lys-NH₂ bisTFA (30)** was synthesized according to the general procedure for SPPS, using MBHA Rink amide resin and **heptanoic acid** as $R^1$-OH. After purification via RP-HPLC, compound **30** (22 mg) was obtained as a white powder. $^1$H NMR (400 MHz, DMSO-$d_6$): δ 8.11 – 7.94 (m, 2H), 7.90 (d, *J* = 7.6 Hz, 1H), 7.83 (d, *J* = 8.4 Hz, 1H), 7.65 (br, 3H), 7.53 – 7.42 (m, 1H), 7.33 – 6.83 (m, 6H), 6.76 (s, 1H), 4.33 – 4.07 (m, 4H), 3.15 – 3.05 (m, 2H), 2.82 – 2.71 (m, 2H), 2.20 – 2.00 (m, 4H), 1.99 – 1.85 (m, 1H), 1.83 – 1.61 (m, 3H), 1.60 – 1.40 (m, 8H), 1.39 – 1.12 (m, 11H), 0.86 (t, *J* = 6.8 Hz, 3H). LCMS (ESI): *m/z* calcd for $C_{27}H_{53}N_{10}O_6^+$ [M + H]$^+$, 613.41; found 613.51.

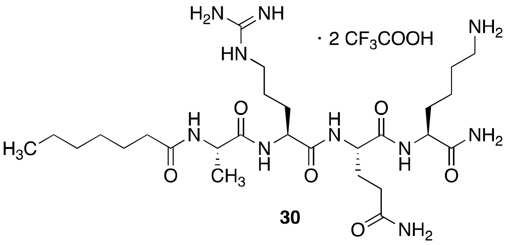

**30**

**H-Lys-Ala-Lys-NH-*n*-hexyl trisTFA (31)** was synthesized according to general procedure A, using **hexyl amine** as $R^2$-$NH_2$. After purification via RP-HPLC, compound **31** (53 mg) was obtained as a white powder. $^1$H NMR (400 MHz, DMSO-$d_6$): δ 8.62 (d, $J$ = 6.8 Hz, 1H), 8.17 (d, $J$ = 8.4 Hz, 1H), 8.10 (br, 3H), 7.85 (t, $J$ = 5.6 Hz, 1H), 7.70 (br, 6H), 4.45 – 4.48 (m, 1H), 4.20 – 4.10 (m, 1H), 3.82 – 3.75 (m, 1H), 3.12 – 2.92 (m, 2H), 2.80 – 2.70 (m, 4H), 1.73 – 1.58 (m, 3H), 1.56 – 1.45 (m, 5H), 1.42 – 1.18 (m, 14H), 0.86 (t, $J$ = 7.2 Hz, 3H). LCMS (ESI): *m/z* calcd for $C_{21}H_{45}N_6O_3^+$ [M + H]$^+$, 429.35; found 429.32.

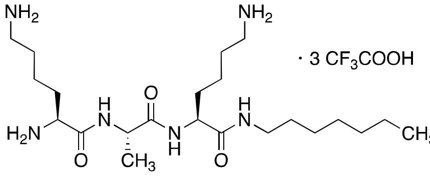


**31**

**H-Lys-Ala-Ala-Lys-NH-*n*-hexyl trisTFA (32)** was synthesized according to general procedure A, using ***n*-hexyl amine** as $R^2$-$NH_2$. After purification via RP-HPLC, compound **32** (40 mg) was obtained as a white powder. $^1$H NMR (400 MHz, DMSO-$d_6$): δ 8.28 – 8.02 (m, 3H), 7.88 – 7.76 (m, 2H), 4.30 – 4.10 (m, 2H), 3.17 – 3.11 (m, 1H), 3.08 – 2.95 (m, 2H), 2.62 – 2.52 (m, 4H), 1.70 – 1.14 (m, 23H), 0.90 – 0.80 (m, 3H). LCMS (ESI): *m/z* calcd for $C_{24}H_{50}N_7O_5^+$ [M + H]$^+$, 500.39; found 500.55.

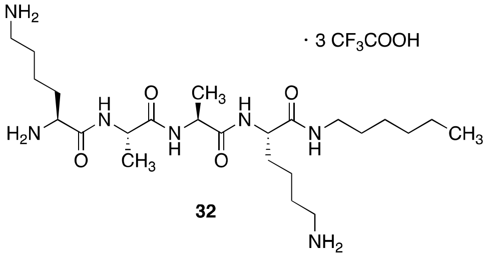


**32**

**H-Ala-Arg-Gln-Lys-NH-*n*-hexyl trisTFA (33)** was synthesized according to general procedure A, using ***n*-hexadecyl amine** as $R^2$-$NH_2$. After purification via RP-HPLC, compound **33** (45 mg) was obtained as a white powder. $^1$H NMR (400 MHz, DMSO-$d_6$): δ 8.53 (d, $J$ = 7.2 Hz, 1H), 8.22 – 7.98 (m, 3H), 7.97 – 7.82 (m, 3H), 7.81 – 7.56 (m, 4H), 7.34 – 6.90 (m, 4H), 6.78 (s, 1H), 4.38 – 4.10 (m, 3H), 3.80 – 3.95 (m, 1H), 3.19 – 2.90 (m, 4H), 2.80 – 2.70 (m, 2H), 2.17 – 2.03 (m, 2H), 1.95 – 1.80 (m, 1H), 1.79 – 1.43 (m, 9H), 1.42 – 1.14 (m, 13H), 0.86 (t, $J$ = 6.0 Hz, 3H). LCMS (ESI): *m/z* calcd for $C_{26}H_{53}N_{10}O_5^+$ [M + H]$^+$, 585.42; found 585.58.

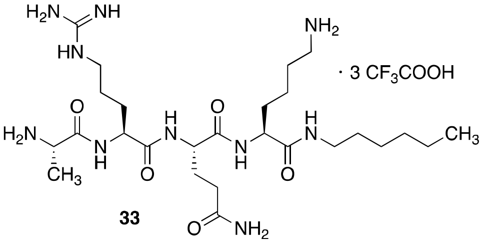


**33**

**H-Lys-Ala-Lys-NH-*n*-hexadecyl trisTFA (34)** was synthesized according to general procedure A, using **hexadecyl amine** as $R^2$-$NH_2$. After purification via RP-HPLC, compound **34** (41 mg) was obtained as a white powder. $^1$H NMR (400 MHz, DMSO-$d_6$): δ 8.55 (d, $J$ = 7.6 Hz, 1H), 8.08 (br, 2H), 7.97 (d, $J$ = 8.0 Hz, 1H), 7.81 (t, $J$ = 5.6 Hz, 1H), 7.78 – 7.58 (m, 5H), 4.38 – 4.28 (m, 1H), 4.15 – 4.07 (m, 1H), 3.75 (br, 1H), 3.12 – 2.95 (m, 3H), 2.60 – 2.40 (m, 4H), 1.75 – 1.18 (m, 41H), 0.86 (t, $J$ = 6.4 Hz, 3H). LCMS (ESI): *m/z* calcd for $C_{31}H_{65}N_6O_3^+$ [M + H]$^+$, 569.51; found 569.54.

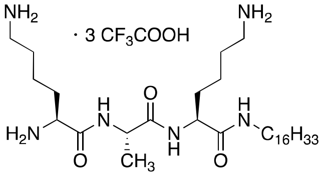


**34**

**H-Lys-Ala-Ala-Lys-NH-*n*-hexadecyl trisTFA (35)** was synthesized according to general procedure A, using ***n*-hexadecyl amine** as $R^2$-$NH_2$. After purification via RP-HPLC, compound **35** (40 mg) was obtained as a white powder. $^1$H NMR (400 MHz, DMSO-$d_6$): δ 8.59 (d, $J$ = 6.8 Hz, 1H), 8.18 (d, $J$ = 7.6 Hz, 1H), 8.15 – 7.98 (m, 3H), 7.90 – 7.84 (m, 1H), 7.83 – 7.42 (m, 7H), 4.43 – 4.36 (m, 1H), 4.30 – 4.24 (m, 1H), 4.23 – 4.12 (m, 1H), 3.82 – 3.72 (m, 1H), 3.10 – 2.95 (m, 2H), 2.84 – 2.67 (m, 4H), 1.75 – 1.46 (m, 7H), 1.42 – 1.15 (m, 33H), 0.85 (t, $J$ = 6.0 Hz, 3H). LCMS (ESI): *m/z* calcd for $C_{34}H_{70}N_7O_5^+$ [M + H]$^+$, 640.5; found 640.5.

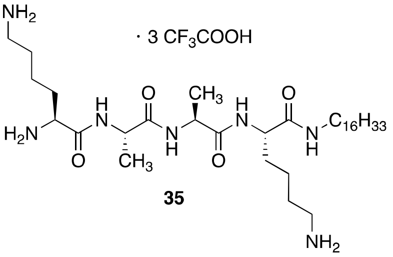


**35**

**H-Ala-Arg-Gln-Lys-NH-*n*-hexadecyl trisTFA (36)** was synthesized according to general procedure A, using ***n*-hexadecyl amine** as $R^2$-$NH_2$. After purification via RP-HPLC, compound **36** (30 mg) was obtained as a white powder. $^1$H NMR (400 MHz, DMSO-$d_6$): δ 8.63 (br, 1H), 8.30 – 8.11 (m, 1H), 7.96 (d, $J$ = 7.6 Hz, 1H), 7.90 – 7.56 (m, 6H), 7.26 (s, 1H), 7.24 – 6.94 (m, 3H), 6.77 (s, 1H), 4.35 – 4.26 (m, 1H), 4.25 – 4.08 (m, 2H), 3.93 – 3.80 (m, 1H), 3.18 – 2.90 (m, 5H), 2.83 – 2.70 (m, 2H), 2.15 – 2.04 (m, 2H), 1.97 – 1.46 (m, 10H), 1.45 – 1.11 (32H), 0.86 (t, $J$ = 6.4 Hz, 3H). LCMS (ESI): *m/z* calcd for $C_{36}H_{73}N_{10}O_5^+$ [M + H]$^+$, 725.58; found 725.81.

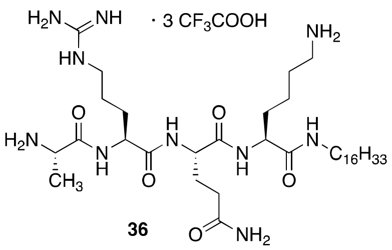


**36**

**Palmitoyl-Lys-Ala-Lys-$NH_2$ bisTFA (37)** was synthesized according to the general procedure for SPPS, using MBHA Rink amide resin and **palmitic acid** as $R^1$-OH. After purification via RP-HPLC, compound **37** (25 mg) was obtained as a white powder. $^1$H NMR (400 MHz, DMSO-$d_6$): δ 7.95 (d, $J$ = 7.2 Hz, 1H), 7.76 (d, $J$ = 7.6 Hz, 1H), 7.68 (br, 6H), 7.25 (s, 1H), 7.02 (s, 1H), 4.36 – 4.07 (m, 3H), 2.83 – 2.70 (m, 4H), 2.17 – 2.08 (m, 2H), 1.73 – 1.60 (m, 2H). LCMS (ESI): $m/z$ calcd for $C_{31}H_{63}N_6O_4^+$ [M + H]$^+$, 583.49; found 583.60.

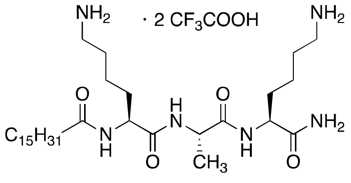

**37**

**Palmitoyl-Lys-Ala-Ala-Lys-$NH_2$ bisTFA (38)** was synthesized according to the general procedure for SPPS, using MBHA Rink amide resin and **palmitic acid** as $R^1$-OH. After purification via RP-HPLC, compound **38** (11 mg) was obtained as a white powder. $^1$H NMR (500 MHz, DMSO): δ 8.05 (d, $J$ = 7.2 Hz, 1H), 8.02 – 7.94 (m, 2H), 7.95 – 7.66 (m, 6H), 7.30 (s, 1H), 7.04 (s, 1H), 4.37 – 4.09 (m, 5H), 2.83 – 2.66 (m, 4H), 2.12 (t, $J$ = 7.4 Hz, 2H), 1.77 – 1.40 (m, 10H), 1.38 – 1.09 (m, 26H), 0.90 – 0.79 (m, 3H). LCMS (ESI): $m/z$ calcd for $C_{34}H_{68}N_7O_5^+$ [M + H]$^+$, 654.53; found 654.70.

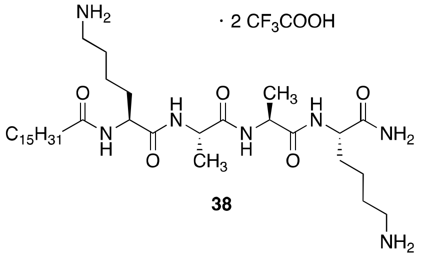

**38**

**Palmitoyl-Ala-Arg-Gln-Lys-$NH_2$ bisTFA (39)** was synthesized according to the general procedure for SPPS, using MBHA Rink amide resin and **palmitic acid** as $R^1$-OH. After purification via RP-HPLC, compound **39** (15 mg) was obtained as a white powder. $^1$H NMR (400 MHz, DMSO-$d_6$): δ 8.11 – 7.94 (m, 2H), 7.90 (d, $J$ = 7.6 Hz, 1H), 7.83 (d, $J$ = 8.4 Hz, 1H), 7.65 (br, 3H), 7.53 – 7.42 (m, 1H), 7.33 – 6.83 (m, 7H), 6.76 (s, 1H), 4.33 – 4.07 (m, 4H), 3.15 – 3.05 (m, 2H), 2.85 – 2.69 (m, 2H), 2.20 – 2.00 (m, 4H), 1.99 – 1.85 (m, 1H), 1.84 – 1.61 (m, 3H), 1.60 – 1.40 (m, 8H), 1.36 – 1.17 (m, 26H), 0.86 (t, $J$ = 6.8 Hz, 3H). LCMS (ESI): $m/z$ alcd for $C_{36}H_{71}N_{10}O_6^+$ [M +H]$^+$, 739.56; found 739.80.

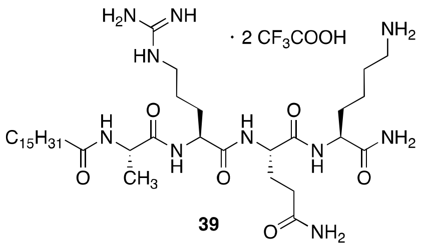

**39**

**Palmitoyl-Lys-Lys-$NH_2$ bisTFA (40)** was synthesized according to the general procedure for SPPS, using MBHA Rink amide resin (0.30 g) and **palmitic acid** as $R^1$-OH. After purification via RP-HPLC, compound **40** (65 mg, 66%) was obtained as a white powder. $^1$H NMR (400 MHz, MeOH-$d_4$): δ 4.37 (dd, $J$ = 9.4, 4.9 Hz, 1H), 4.25 (dd, $J$ = 8.0, 6.4 Hz, 1H), 2.93 (t, $J$ = 7.5 Hz, 4H), 2.30 – 2.16 (m, 2H), 2.05 – 1.21 (m, 39H), 0.95 – 0.85 (m, 3H). $^{13}$C NMR (101 MHz, MeOH-$d_4$): δ 176.8, 174.6, 54.98, 54.1, 40.63, 40.59, 36.9, 33.2, 32.7, 32.2, 30.92, 30.90, 30.88, 30.80, 30.61, 30.59, 30.5, 28.2, 28.1, 27.1, 23.90, 23.86, 14.6. HRMS (ESI): $m/z$ calcd for $C_{28}H_{57}N_5NaO_3^+$ [M + Na]$^+$ 534.4354; found 534.4367.

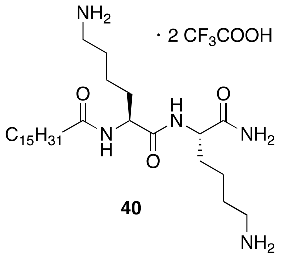

**40**

**Palmitoyl-Lys-Arg-$NH_2$ bisTFA (41)** was synthesized according to the general procedure for SPPS, using MBHA Rink amide resin (0.30 g) and **palmitic acid** as $R^1$-OH. After purification via RP-HPLC, compound **41** (32 mg, 27%) was obtained as a white powder. $^1$H NMR (400 MHz, MeOH-$d_4$): δ 4.38 (dd, $J$ = 8.6, 4.8 Hz, 1H), 4.33 – 4.18 (m, 1H), 4.14 (t, $J$ = 7.2 Hz, 1H), 3.21 (m, 1H), 2.93 (m, 3H), 2.39 – 2.15 (m, 2H), 2.10 – 1.04 (m, 41H), 0.90 (t, $J$ = 6.5 Hz, 3H). $^{13}$C NMR (101 MHz, MeOH-$d_4$): δ 177.1, 177.0, 176.8, 176.4, 175.9, 175.4, 174.5, 158.8, 79.7, 79.4, 79.1, 55.7, 54.9, 54.5, 53.8, 51.5, 42.1, 40.59, 40.55, 36.9, 36.6, 33.2, 32.2, 31.85, 31.79, 30.95, 30.92 30.91, 30.89, 30.82, 30.79, 30.62, 30.60, 30.58, 30.52, 28.4, 28.2, 28.0, 27.1, 26.9, 26.4, 24.2, 24.0, 23.89. 23.86, 17.5, 14.6. HRMS (ESI): $m/z$ calcd for $C_{28}H_{57}N_7O_3^+$ [M + H]$^+$ , 540.4596; found 540.4594.

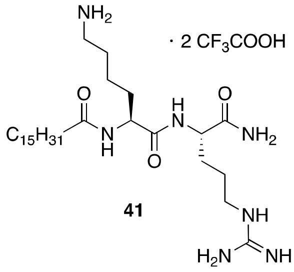

**41**

**Palmitoyl-Arg-Arg-NH2 bisTFA (42)** was synthesized according to the general procedure for SPPS, using MBHA Rink amide resin (0.30 g) and **palmitic acid** as $R^1$-OH. After purification via RP-HPLC, compound **42** (11 mg, 8.8%) was obtained as a white powder. $^1$H NMR (400 MHz, MeOH-d4): δ 4.53 – 4.23 (m, 2H), 3.72 – 2.96 (m, 8H)[1], 2.27 (t, J = 7.6 Hz, 2H), 2.08 – 1.54 (m, 9H), 1.54 – 1.11 (m, 21H), 0.92 (t, J = 6.6 Hz, 3H). $^{13}$C NMR (101 MHz, MeOH-d4): δ 176.8, 176.4, 174.3, 158.8, 79.7, 79.4, 79.1, 54.6, 53.8, 50.0, 42.12, 42.09, 36.9, 33.2, 30.93, 30.92, 30.90, 30.8, 30.63, 30.60, 30.58, 30.52, 30.5, 30.0, 27.1, 26.41, 26.39, 23.9, 14.6. HRMS (ESI): *m/z* calcd for $C_{28}H_{57}N_9O_3^+$ $[M + H]^+$ , 568.4657; found 568.4662.

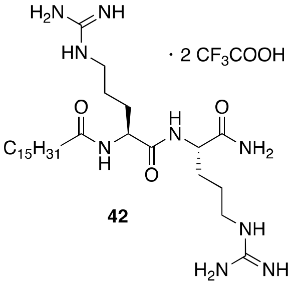

42

**Palmitoyl-Arg-Lys-NH2 bisTFA (43)** was synthesized according to the general procedure for SPPS, using MBHA Rink amide resin (0.30 g) and **palmitic acid** as $R^1$-OH. After purification via RP-HPLC, compound **43** (20 mg, 17%) was obtained as a white powder. $^1$H NMR (400 MHz, MeOH-*d*$_4$): δ 4.38 (dd, *J* = 9.4, 4.9 Hz, 1H), 4.29 (dd, *J* = 7.6, 6.0 Hz, 1H), 3.20 (t, *J* = 6.8 Hz, 2H), 2.93 (t, *J* = 7.5 Hz, 2H), 2.25 (dd, *J* = 8.4, 6.8 Hz, 2H), 2.03 – 1.11 (m, 36H), 0.97 – 0.82 (m, 3H). $^{13}$C NMR (101 MHz, MeOH-*d*$_4$): δ 176.8, 176.7, 174.3, 158.8, 54.6, 54.0, 42.2, 40.7, 36.9, 33.2, 32.8, 30.95, 30.93, 30.91, 30.8, 30.64, 30.62, 30.5, 30.0, 28.1, 27.1, 26.4, 23.9, 14.6. HRMS (ESI): *m/z* calcd for $C_{28}H_{57}N_7O_3^+$ $[M + H]^+$ , 540.4596; found 540.4599.

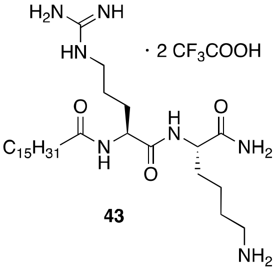

43

**Palmitoyl-Arg-Gln-Lys-NH2 bisTFA (44)** was synthesized according to the general procedure for SPPS, using MBHA Rink amide resin and **palmitic acid** as $R^1$-OH. After purification via RP-HPLC, compound **44** (30 mg) was obtained as a white powder. $^1$H NMR (500 MHz, DMSO-*d*$_6$): δ 8.09 (d, *J* = 7.3 Hz, 1H), 8.06 (d, *J* = 7.3 Hz, 1H), 7.82 (d, *J* = 8.1 Hz, 1H), 7.70 (s, 3H), 7.62 – 7.55 (m, 1H), 7.53 – 6.70 (m, 8H), 4.24 – 4.03 (m, 3H), 3.17 – 3.01 (m, 2H), 2.75 (t, *J* = 7.7 Hz, 2H), 2.56 – 2.51 (m, 2H), 2.19 – 2.04 (m, 4H), 1.96 – 1.84 (m, 1H), 1.82 – 1.61 (m, 3H), 1.60 – 1.41 (m, 8H), 1.41 – 1.17 (m, 26H), 0.89 – 0.81 (m, 3H). $^{13}$C NMR (126 MHz, DMSO-*d*$_6$): δ 173.92, 173.45, 172.91, 171.81, 170.98, 156.71, 52.57, 52.49, 52.17, 40.42, 38.67, 35.13, 31.37, 31.28, 31.22, 29.07, 29.05, 29.00, 28.97, 28.81, 28.73, 28.69, 27.62, 26.63, 25.17, 25.04, 22.23, 22.08, 13.94.

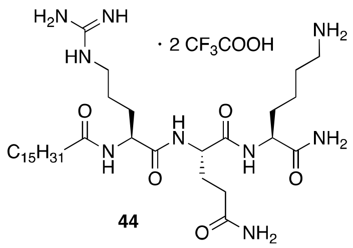

44

**Palmitoyl-Lys-Pro-Lys-NH2 bisTFA (45)** was synthesized according to the general procedure for SPPS, using MBHA Rink amide resin (0.30 g) and **palmitic acid** as $R^1$-OH. After purification via RP-HPLC, compound **45** (57 mg, 44%) was obtained as a white powder. $^1$H NMR (400 MHz, MeOH-*d*$_4$): δ 4.60 (dd, *J* = 8.1, 6.2 Hz, 1H), 4.42 (dd, *J* = 8.3, 5.2 Hz, 1H), 4.28 (dd, *J* = 9.1, 5.1 Hz, 1H), 3.98 – 3.83 (m, 1H), 3.66 (dt, *J* = 12.8, 6.6 Hz, 1H), 2.95 (t, *J* = 7.4 Hz, 4H), 2.35 – 2.15 (m, 3H), 2.16 – 1.40 (m, 15H), 1.29 (s, 22H), 0.98 – 0.83 (m, 3H). $^{13}$C NMR (101 MHz, MeOH-*d*$_4$): δ 176.8, 176.4, 174.6, 173.1, 111.5, 61.9, 54.3, 52.5, 40.65, 40.57, 36.7, 33.2, 32.5, 31.9, 30.91, 30.87, 30.8, 30.6, 30.5, 28.3, 28.0, 27.0, 26.2, 23.86, 23.84, 23.5, 14.6. HRMS (ESI): *m/z* calcd for $C_{33}H_{64}N_6O_4^+$ $[M + Na]^+$ , 631.4881; found 631.4875.

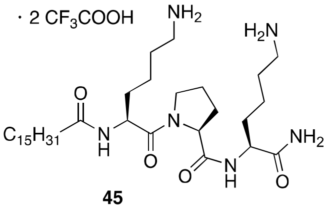

45

**Palmitoyl-Lys-Ala-Pro-Lys-NH2 bisTFA (46)** was synthesized according to the general procedure for SPPS, using MBHA Rink amide resin (0.30 g) and **palmitic acid** as $R^1$-OH. After purification via RP-HPLC, compound **46** (78 mg, 55%) was obtained as a white powder. $^1$H NMR (400 MHz, MeOH-*d*$_4$): δ 4.61 (q, *J* = 7.0 Hz, 1H), 4.42 (dd, *J* = 8.5, 4.5 Hz, 1H), 4.34 (dt, *J* = 8.6, 5.6 Hz, 2H), 3.86 – 3.76 (m, 1H), 3.67 (dt, *J* = 9.8, 6.4 Hz, 1H), 2.93 (q, *J* = 7.4 Hz, 4H), 2.23 (t, *J* = 7.6 Hz, 3H), 2.15 – 1.18 (m, 44H), 0.98 – 0.79 (m, 3H). $^{13}$C NMR (101 MHz, MeOH-*d*$_4$): δ 176.7, 176.6, 174.5, 174.0, 174.0, 173.7, 61.9, 54.2, 54.0, 48.8, 40.7, 40.6, 36.9, 33.2, 32.6, 32.4, 30.91, 30.89, 30.87, 30.78, 30.64, 30.59, 30.5, 28.2, 28.0, 27.1, 26.2, 23.85, 23.83, 23.76, 17.0, 14.6. HRMS (ESI): *m/z* calcd for $C_{36}H_{69}N_7O_5^+$ $[M + Na]^+$ , 702.5252; found 702.5249.

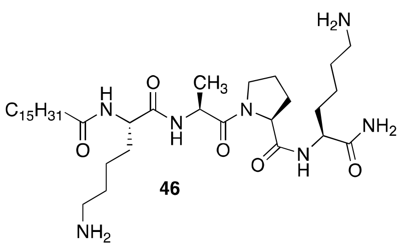

46

[1] Integration is assumed as they are overlapping with the solvent.

**Palmitoyl-Lys-Pro-Ala-Lys-NH2 bisTFA (47)** was synthesized according to the general procedure for SPPS, using MBHA Rink amide resin (0.30 g) and **palmitic acid** as R[1]-OH. After purification via RP-HPLC, compound **47** (76 mg, 54%) was obtained as a white powder. $^1$H NMR (400 MHz, MeOH-$d_4$): δ 4.59 (dd, $J$ = 7.9, 6.3 Hz, 1H), 4.43 (dd, $J$ = 8.2, 4.6 Hz, 1H), 4.38 – 4.20 (m, 2H), 4.02 – 3.80 (m, 1H), 3.77 – 3.57 (m, 1H), 2.93 (q, $J$ = 7.9 Hz, 3H), 2.36 – 2.14 (m, 3H), 2.13 – 1.21 (m, 37H), 1.02 – 0.74 (m, 3H). $^{13}$C NMR (101 MHz, MeOH-$d_4$): δ 176.6, 176.4, 175.1, 174.7, 173.0, 61.9, 54.1, 52.7, 51.1, 40.64, 40.58, 36.7, 33.2, 32.7, 31.9, 30.92, 30.90, 30.89, 30.88, 30.77, 30.75, 30.6, 30.5, 28.3, 28.1, 27.0, 26.2, 23.87, 23.80, 23.6, 17.8, 14.6. HRMS (ESI): $m/z$ calcd for $C_{36}H_{69}N_7O_5^+$ [M + Na]$^+$ , 702.5252; found 702.5252.

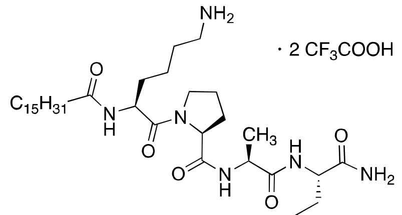


**47**

**Palmitoyl-Arg-His-NH2 bisTFA (48)** was synthesized according to the general procedure for SPPS, using MBHA Rink amide resin (0.30 g) and **palmitic acid** as R[1]-OH. After purification via RP-HPLC, compound **48** (12 mg, 18%) was obtained as a white powder. $^1$H NMR (500 MHz, MeOH-$d_4$): δ(ppm) 8.92 – 8.60 (m, 1H), 7.36 (s, 1H), 4.75 – 4.68 (m, 1H), 4.27 (dd, $J$ = 8.1, 5.7 Hz, 1H), 3.31 – 3.06 (m, 4H), 2.27 (t, $J$ = 7.6 Hz, 2H), 1.92 – 1.55 (m, 6H), 1.30 (s, 24H), 0.92 (t, $J$ = 6.8 Hz, 3H). $^{13}$C NMR (126 MHz, MeOH-$d_4$): δ(ppm) 176.8, 174.23, 174.21, 158.6, 54.7, 53.2, 41.9, 36.7, 33.1, 30.77, 30.74, 30.64, 30.5, 30.4, 29.7, 26.9, 26.3, 23.7, 14.4. HRMS (ESI): $m/z$ calcd for $C_{28}H_{53}N_8O_3^+$ [M + H]$^+$ , 549.4196; found 549.4242.

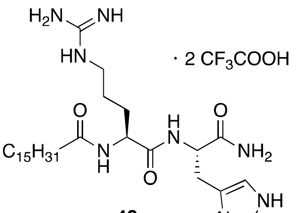


**48**

**Palmitoyl-Arg-Ala-Phe-NH2 TFA (49)** was synthesized according to the general procedure for SPPS, using MBHA Rink amide resin (1.0 g) and **palmitic acid** as R[1]-OH. After purification via RP-HPLC, compound **49** (17 mg, 5%) was obtained as a white powder. $^1$H NMR (400 MHz, DMSO-$d_6$): δ 8.11 – 7.98 (m, 2H), 7.83 (d, $J$ = 8.0 Hz, 1H), 7.46 – 7.38 (m, 1H), 7.36 (s, 1H), 7.30 – 7.16 (m, 6H), 7.14 – 6.56 (m, 4H), 4.42 – 4.36 (m, 1H), 4.29 – 4.12 (m, 2H), 3.12 – 2.98 (m, 3H), 2.90 – 2.78 (m, 1H), 2.18 – 2.07 (m, 2H), 1.70 – 1.56 (m, 5H), 1.31 – 1.20 (m, 24H), 0.86 (t, $J$ = 6.4 Hz, 3H). HRMS (ESI): $m/z$ calcd for $C_{34}H_{60}N_7O_4^+$ [M + H]$^+$, 630.4701; found 630.4683.

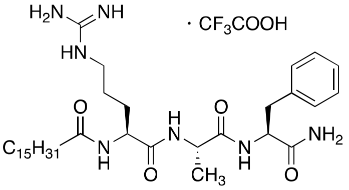


**49**

**Palmitoyl-Arg-Gln-Hph-NH2 TFA (50)** was synthesized according to the general procedure for SPPS, using MBHA Rink amide resin (1.0 g) and **palmitic acid** as R[1]-OH. After purification via RP-HPLC, compound **50** (15, 3%) was obtained as a white powder. $^1$H NMR (400 MHz, DMSO-$d_6$): δ 8.24 (d, $J$ = 7.2 Hz, 1H), 8.09 (d, $J$ = 7.2 Hz, 1H), 7.92 (d, $J$ = 8.0 Hz, 1H), 7.50 – 7.40 (m, 1H), 7.38 – 6.59 (m, 13H), 4.30 – 4.10 (m, 3H), 3.17 – 3.03 (m, 2H), 2.67 – 2.53 (m, 2H), 2.22 – 2.07 (m, 4H), 2.03 – 1.74 (m, 4H), 1.73 – 1.60 (m, 1H), 1.60 – 1.40 (m, 5H), 1.30 – 1.16 (m, 24H), 0.86 (t, $J$ = 6.8 Hz, 3H). HRMS (ESI): $m/z$ calcd for $C_{37}H_{65}N_8O_5^+$ [M + H]$^+$, 701.5072; found 701.5052.

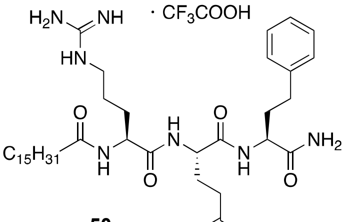


**50**

**Palmitoyl-Ala-Arg-Gln-Phe-NH2 TFA (51)** was synthesized according to the general procedure for SPPS, using MBHA Rink amide resin (1.0 g) and **palmitic acid** as R[1]-OH. After purification via RP-HPLC, compound **51** (16 mg, 3%) was obtained as a white powder. $^1$H NMR (400 MHz, DMSO-$d_6$): δ 8.08 – 7.99 (m, 2H), 7.98 – 7.84 (m, 2H), 7.45 – 7.38 (m, 2H), 7.37 – 6.56 (m, 12H), 4.60 – 4.12 (m, 4H), 3.55 – 3.40 (m, 1H), 3.12 – 2.93 (m, 3H), 2.90 – 2.64 (m, 1H), 2.20 – 1.98 (m, 4H), 1.90 – 1.61 (m, 5H), 1.32 – 1.15 (m, 27H), 0.86 (t, $J$ = 6.8 Hz, 3H). HRMS (ESI): $m/z$ calcd for $C_{39}H_{68}N_9O_6^+$ [M + H]$^+$, 758.5287; found 758.5269.

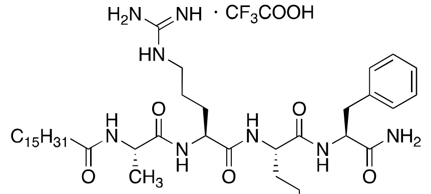


**51**

**Palmitoyl-Lys-Ala-Phe-NH2 TFA (52)** was synthesized according to the general procedure for SPPS, using MBHA Rink amide resin (1.0 g) and **palmitic acid** as R[1]-OH. After purification via RP-HPLC, compound **52** (130 mg, 30%) was obtained as a white powder. $^1$H NMR (500 MHz, DMSO-$d_6$): δ 7.98 (d, $J$ = 7.0 Hz, 1H), 7.95 (d, $J$ = 7.8 Hz, 1H), 7.78 (d, $J$ = 8.2 Hz, 1H), 7.66 (s, 3H), 7.34 (s, 1H), 7.27 – 7.22 (m, 2H), 7.22 – 7.15 (m, 3H), 7.09 (s, 1H), 4.37 (td, $J$ = 8.5, 5.0 Hz, 1H), 4.22 – 4.12 (m, 2H), 3.01 (dd, $J$ = 13.8, 5.0 Hz, 1H), 2.83 (dd, $J$ = 13.8, 8.8 Hz, 1H), 2.75 (s, 2H), 2.14 – 2.08 (m, 2H), 1.65 – 1.55 (m, 1H), 1.55 – 1.41 (m, 5H), 1.38 – 1.18 (m, 28H), 1.14 (d, $J$ = 7.1 Hz, 3H), 0.88 – 0.82 (m, 3H). $^{13}$C NMR (126 MHz, DMSO-$d_6$): δ 172.63, 172.52, 171.80, 171.62, 140.85, 136.22, 129.79, 129.15, 127.99,

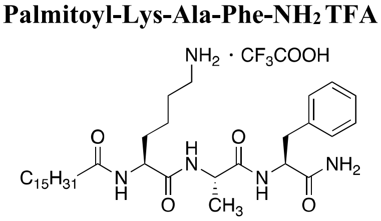


**52**

126.20, 53.64, 52.23, 49.05, 47.90, 38.73, 37.42, 35.13, 31.27, 31.13, 29.03, 29.02, 28.99, 28.94, 28.79, 28.68, 28.66, 26.64, 25.21, 22.29, 22.08, 17.90, 13.94. LCMS (ESI): *m/z* calcd for $C_{34}H_{60}N_5O_4^+$ [M + H]$^+$, 602.46; found 602.28.

**Palmitoyl-Lys-Ala-His-NH2 bisTFA (53)** was synthesized according to the general procedure for SPPS, using MBHA Rink amide resin (0.30 g) and **palmitic acid** as R$^1$-OH. After purification via RP-HPLC, compound **53** (25 mg, 16%) was obtained as a white powder. $^1$H NMR (500 MHz, MeOH-*d4*): δ 8.83 – 8.67 (m, 1H), 7.36 (s, 1H), 4.65 (dd, *J* = 7.9, 5.2 Hz, 1H), 4.30 (dd, *J* = 8.3, 5.9 Hz, 1H), 4.23 (q, *J* = 7.2 Hz, 1H), 3.30 – 3.25 (m, 1H), 3.13 (dd, *J* = 15.3, 7.9 Hz, 1H), 2.97 – 2.91 (m, 2H), 2.25 (t, *J* = 7.6 Hz, 2H), 1.83 (ddt, *J* = 13.5, 8.7, 6.4 Hz, 1H), 1.78 – 1.20 (m, 34H), 0.90 (t, *J* = 6.9 Hz, 3H). $^{13}$C NMR (126 MHz, MeOH-*d4*): δ 176.6, 175.1, 174.5, 174.2, 163.2 (TFA), 162.9 (TFA), 134.9, 131.2, 118.6, 54.5, 53.3, 51.1, 40.5, 36.8, 33.1, 32.3, 30.79, 30.77, 30.75, 30.7, 30.49, 30.47, 30.4, 28.1, 28.0, 26.9, 26.8, 23.73, 23.69, 17.4, 14.4. HRMS (ESI): *m/z* calcd for $C_{31}H_{58}N_7O_4^+$ [M + H]$^+$ , 592.4506; found 592.4551.

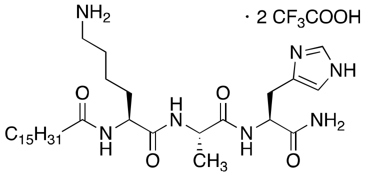

53

**Palmitoyl-Phe-Ala-Phe-NH2 (54)** was synthesized according to the general procedure for SPPS, using MBHA Rink amide resin (1.0 g) and **palmitic acid** as R$^1$-OH. After cleavage from the resin, the crude material was purified via flash column chromatography ($SiO_2$, MeOH / DCM, from 1 : 99 to 3 : 17) instead of using RP-HPLC. Compound **55** (160 mg, 43%) was obtained as an off-white solid. $^1$H NMR (500 MHz, DMSO-*d6*): δ 8.09 (d, *J* = 7.1 Hz, 1H), 7.99 (d, *J* = 8.3 Hz, 1H), 7.79 (d, *J* = 8.2 Hz, 1H), 7.34 (s, 1H), 7.28 – 6.99 (m, 8H), 4.49 (ddd, *J* = 10.5, 8.3, 4.0 Hz, 1H), 4.41 (td, *J* = 8.4, 5.1 Hz, 1H), 4.20 (p, *J* = 7.1 Hz, 1H), 2.99 (ddd, *J* = 23.6, 13.9, 4.5 Hz, 2H), 2.84 (dd, *J* = 13.8, 8.6 Hz, 1H), 2.72 – 2.66 (m, 1H), 1.99 (t, *J* = 7.3 Hz, 2H), 1.93 – 1.83 (m, 2H), 1.57 – 0.95 (m, 23H), 0.85 (t, *J* = 6.8 Hz, 3H). HRMS (ESI): *m/z* calcd for $C_{37}H_{56}N_4NaO_4^+$ [M + Na]$^+$ 643.4194; found 643.4189.

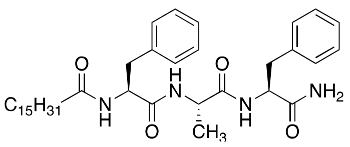

54

**Palmitoyl-Lys-Ala-Ala-Phe-NH2 TFA (55)** was synthesized according to the general procedure for SPPS, using MBHA Rink amide resin (0.75 g) and **palmitic acid** as R$^1$-OH. After purification via RP-HPLC, compound **55** (119 mg, 32%) was obtained as a white powder.$^1$H NMR (500 MHz, DMSO-*d6*): δ 8.00 (d, *J* = 7.0 Hz, 1H), 7.94 (d, *J* = 7.4 Hz, 2H), 7.72 (d, *J* = 8.2 Hz, 1H), 7.61 (br, 2H), 7.09 – 7.33 (m, 7H), 4.35 – 4.41 (m, 1H), 4.12 – 4.24 (m, 3H), 2.98 – 3.05 (m, 1H), 2.79 – 2.86 (m, 1H), 2.71 – 2.76 (m, 2H), 2.11 (t, *J* = 7.4 Hz, 2H), 1.59 – 1.66 (m, 1H), 1.44 – 1.54 (m, 5H), 1.21 – 1.34 (m, 27H), 1.17 (d, *J* = 7.1, 3H), 1.14 (d, *J* = 7.1 Hz, 3H), 0.85 (t, *J* = 6.7 Hz, 3H). $^{13}$C NMR (126 MHz, DMSO-*d6*): δ 172.60, 172.50, 172.01, 171.67, 158.62 – 156.90 (m, TFA), 137.73, 129.19, 127.98, 126.20, 53.58, 52.28, 48.51, 48.17, 38.72, 37.43, 35.11, 31.28, 31.17, 29.04, 28.99, 28.95, 28.81, 28.69, 28.66, 26.71, 25.21, 22.28, 22.08, 17.85, 17.81, 13.94. HRMS (ESI): *m/z* calcd for $C_{37}H_{65}N_6O_5^+$ [M + H]$^+$: 673.50164; found: 673.50194.

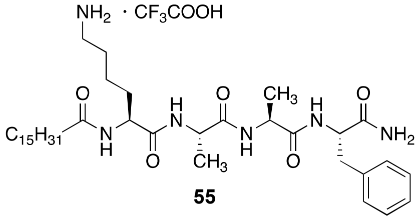

55

51

**Palmitoyl-Lys-Ala-Ala-Hph-NH2 TFA (56)** was synthesized according to the general procedure for SPPS, using MBHA Rink amide resin (1.0 g) and **palmitic acid** as R$^1$-OH. After purification via RP-HPLC, compound **56** (46 mg, 10%) was obtained as a white powder. $^1$H NMR (500 MHz, DMSO-*d6*): δ 8.02 (d, *J* = 7.1 Hz, 2H), 7.95 (d, *J* = 7.6 Hz, 1H), 7.81 (d, *J* = 8.0 Hz, 1H), 7.62 (s, 3H), 7.30 – 7.22 (m, 3H), 7.20 – 7.13 (m, 3H), 7.09 – 7.05 (m, 1H), 4.30 – 4.22 (m, 2H), 4.22 – 4.11 (m, 2H), 2.78 – 2.70 (m, 2H), 2.64 – 2.51 (m, 2H), 2.11 (t, *J* = 7.4 Hz, 2H), 2.01 – 1.90 (m, 1H), 1.87 – 1.76 (m, 1H), 1.69 – 1.58 (m, 1H), 1.55 – 1.43 (m, 5H), 1.35 – 1.14 (m, 32H), 0.88 – 0.82 (m, 3H). $^{13}$C NMR (126 MHz, DMSO-*d6*): δ 173.27, 172.58, 172.12, 171.90, 171.73, 141.37, 128.29, 128.26, 125.80, 52.35, 52.00, 48.44, 48.23, 38.72, 35.11, 33.83, 31.27, 31.13, 29.03, 29.01, 28.99, 28.94, 28.80, 28.68, 28.65, 26.63, 25.19, 22.28, 22.07, 17.92, 17.73, 13.94. LCMS (ESI): *m/z* calcd for $C_{38}H_{67}N_6O_5^+$ [M + H]$^+$, 687.5; found 687.2.

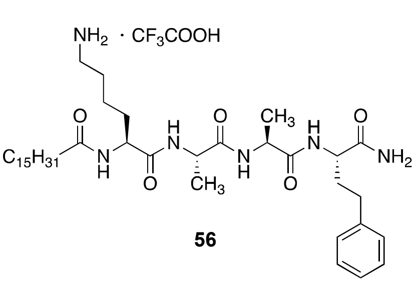

56

**Palmitoyl-Lys-Ala-Ala-His-$NH_2$ bisTFA (57)** was synthesized according to the general procedure for SPPS, using MBHA Rink amide resin (0.30 g) and **palmitic acid** as $R^1$-OH. After purification via RP-HPLC, compound **57** (32 mg, 18%) was obtained as a white powder. $^1$H NMR (500 MHz, MeOH-$d_4$): δ 8.78 (s, 1H), 7.38 (s, 1H), 4.65 (dd, $J$ = 8.6, 4.8 Hz, 1H), 4.39 – 4.08 (m, 3H), 3.36 – 3.33 (m, 1H), 3.12 (dd, $J$ = 15.4, 8.7 Hz, 1H), 3.01 – 2.87 (m, 2H), 2.28 (t, $J$ = 8.2, 6.9 Hz, 2H), 1.88 – 1.77 (m, 1H), 1.77 – 1.22 (m, 37H), 0.90 (t, $J$ = 6.9 Hz, 3H). $^{13}$C NMR (126 MHz, MeOH-$d_4$): δ 177.1, 175.3, 174.91, 174.88, 174.3, 135.0, 131.3, 118.6, 106.4, 55.5, 53.3, 51.03, 50.98, 40.5, 36.7, 33.1, 32.0, 30.78, 30.77, 30.75, 30.7, 30.51, 30.47, 30.4, 28.1, 26.8, 23.8, 23.7, 17.6, 17.4, 14.4. HRMS (ESI): $m/z$ calcd for $C_{34}H_{63}N_8O_5^+$ $[M + H]^+$ , 663.4921; found 663.4910.

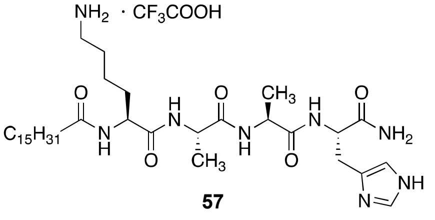


**57**

**Palmitoyl-Lys-Ala-Ala-Trp-$NH_2$ TFA (58)** was synthesized according to the general procedure for SPPS, using MBHA Rink amide resin (0.5 g) and **palmitic acid** as $R^1$-OH. After purification via RP-HPLC, compound **58** (15 mg, 6%) was obtained as a white powder. $^1$H NMR (400 MHz, DMSO-$d_6$): δ 8.04 – 7.95 (m, 2H), 7.92 (d, $J$ = 8.0 Hz, 1H), 7.71 (d, $J$ = 8.0 Hz, 1H), 7.68 – 7.50 (m, 4H), 7.37 – 7.26 (m, 2H), 7.15 – 7.00 (m, 3H), 6.99 – 6.92 (m, 2H), 4.49 – 4.32 (m, 1H), 4.30 – 4.10 (m, 3H), 3.13 (dd, $J$ = 5.6 Hz, 14.4 Hz, 1H), 2.98 (dd, $J$ = 7.6 Hz, 14.8 Hz, 1H), 2.80 – 2.68 (m, 2H), 2.18 – 2.05 (m, 2H), 1.74 – 1.56 (m, 1H), 1.55 – 1.40 (m, 5H), 1.40 – 1.10 (m, 32H), 0.86 (t, $J$ = 6.8 Hz, 3H). LCMS (ESI): $m/z$ calcd for $C_{39}H_{66}N_7O_5^+$ $[M + H]^+$, 712.51; found 712.74.

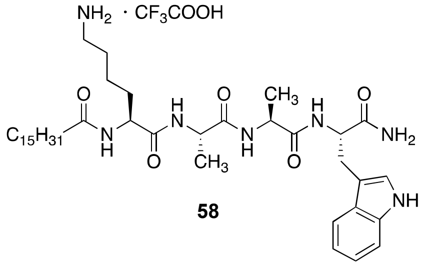


**58**

**Palmitoyl-Phe-Ala-Ala-Phe-$NH_2$ (59)** was synthesized according to the general procedure for SPPS, using MBHA Rink amide resin (1.0 g) and **palmitic acid** as $R^1$-OH. After cleavage from the resin, the crude material was purified via flash column chromatography ($SiO_2$, MeOH / DCM, from 1 : 99 to 3 : 17) instead of using RP-HPLC. Compound **61** (143 mg, 34%) was obtained as an off-white solid. $^1$H NMR (500 MHz, DMSO): δ 8.13 (d, $J$ = 7.1 Hz, 1H), 8.02 – 7.88 (m, 2H), 7.79 (d, $J$ = 8.2 Hz, 1H), 7.33 (s, 1H), 7.28 – 7.12 (m, 8H), 7.08 (s, 1H), 4.50 (ddd, $J$ = 10.5, 8.3, 4.0 Hz, 1H), 4.39 (td, $J$ = 8.5, 5.1 Hz, 1H), 4.27 – 4.14 (m, 2H), 3.01 (ddd, $J$ = 13.9, 4.5, 2.2 Hz, 2H), 2.82 (dd, $J$ = 13.8, 8.8 Hz, 1H), 2.71 (dd, $J$ = 13.9, 10.5 Hz, 1H), 1.99 (t, $J$ = 7.3 Hz, 2H), 1.38 – 0.97 (m, 27H), 0.90 – 0.80 (m, 3H). HRMS (ESI): $m/z$ calcd for $C_{40}H_{61}N_5NaO_5^+$ $[M + Na]^+$ 714.4565; found 714.4552.

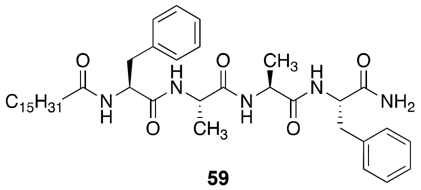


**59**

**Palmitoyl-Lys-Pro-Ala-Phe-$NH_2$ TFA (60)** was synthesized according to the general procedure for SPPS, using MBHA Rink amide resin (1.0 g) and **palmitic acid** as $R^1$-OH. After purification via RP-HPLC, compound **60** (44 mg, 9%) was obtained as a white powder. $^1$H NMR (500 MHz, DMSO-$d_6$): δ 8.07 (d, $J$ = 6.9 Hz, 1H), 8.01 (d, $J$ = 7.9 Hz, 1H), 7.71 – 7.60 (m, 4H), 7.30 (d, $J$ = 2.1 Hz, 1H), 7.28 – 7.14 (m, 5H), 7.12 (d, $J$ = 2.1 Hz, 1H), 4.52 – 4.43 (m, 1H), 4.38 (td, $J$ = 8.6, 4.9 Hz, 1H), 4.29 (dd, $J$ = 8.2, 4.4 Hz, 1H), 4.14 – 4.04 (m, 1H), 3.70 – 3.61 (m, 1H), 3.56 – 3.48 (m, 1H), 3.05 (dd, $J$ = 13.9, 4.9 Hz, 1H), 2.83 (dd, $J$ = 13.9, 8.9 Hz, 1H), 2.78 – 2.69 (m, 2H), 2.09 (td, $J$ = 7.3, 2.3 Hz, 2H), 2.04 – 1.95 (m, 1H), 1.93 – 1.72 (m, 3H), 1.67 – 1.42 (m, 5H), 1.23 (d, $J$ = 4.9 Hz, 25H), 1.13 (d, $J$ = 7.2 Hz, 3H), 0.88 – 0.82 (m, 3H). $^{13}$C NMR (126 MHz, DMSO-$d_6$): δ 172.64, 172.12, 171.80, 171.58, 170.39, 170.38, 137.79, 129.15, 127.98, 126.22, 59.38, 53.44, 50.02, 48.80, 46.84, 38.71, 37.36, 34.89, 31.27, 30.55, 29.03, 29.02, 28.99, 28.96, 28.94, 28.76, 28.68, 28.65, 26.70, 25.19, 24.44, 22.07, 21.96, 17.62, 13.93. LCMS (ESI): $m/z$ calcd for $C_{39}H_{67}N_6O_5^+$ $[M + H]^+$, 699.52; found 699.82.

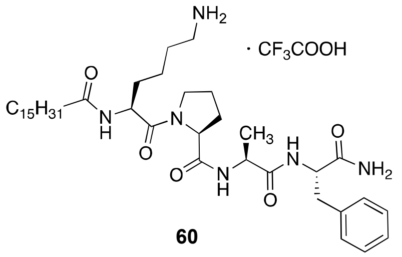


**60**

**Palmitoyl-Lys-Pro-Ala-Hph-NH2 TFA (61)** was synthesized according to the general procedure for SPPS, using MBHA Rink amide resin (1.0 g) and **palmitic acid** as R[1]-OH. After purification via RP-HPLC, compound **61** (34 mg, 7%) was obtained as a white powder. $^1$H NMR (500 MHz, DMSO-$d_6$): δ 8.12 (d, $J$ = 6.9 Hz, 1H), 8.02 (d, $J$ = 7.9 Hz, 1H), 7.77 (d, $J$ = 8.1 Hz, 1H), 7.65 (s, 3H), 7.32 – 7.22 (m, 3H), 7.22 – 7.13 (m, 3H), 7.09 (d, $J$ = 2.1 Hz, 1H), 4.47 (td, $J$ = 8.3, 5.5 Hz, 1H), 4.39 – 4.28 (m, 1H), 4.26 – 4.09 (m, 2H), 3.72 – 3.61 (m, 1H), 3.57 – 3.49 (m, 1H), 2.79 – 2.66 (m, 2H), 2.66 – 2.52 (m, 1H), 2.13 – 1.71 (m, 8H), 1.68 – 1.57 (m, 1H), 1.56 – 1.40 (m, 5H), 1.38 – 1.04 (m, 30H), 0.87 – 0.83 (m, 3H). $^{13}$C NMR (126 MHz, DMSO-$d_6$): δ 173.30, 172.16, 172.08, 171.67, 170.38, 141.33, 128.29, 125.83, 59.46, 51.85, 50.09, 48.72, 46.85, 38.71, 34.88, 33.74, 31.27, 31.23, 30.52, 29.04, 29.02, 28.99, 28.94, 28.76, 28.68, 28.65, 26.70, 25.19, 24.42, 22.07, 21.99, 17.58, 13.94. LCMS (ESI): $m/z$ calcd for $C_{40}H_{69}N_6O_5^+$ [M + H]$^+$, 713.53; found 713.72.

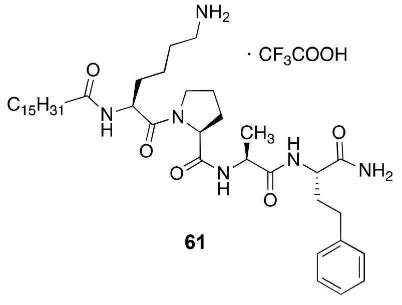


61

**Palmitoyl-Lys-Pro-Ala-Trp-NH2 TFA (62)** was synthesized according to the general procedure for SPPS, using MBHA Rink amide resin (0.50 g) and **palmitic acid** as R[1]-OH. After purification via RP-HPLC, compound **62** (20 mg, 8%) was obtained as a white powder. $^1$H NMR (500 MHz, DMSO-$d_6$): δ 10.81 (s, 1H), 8.09 (d, $J$ = 6.9 Hz, 1H), 7.99 (d, $J$ = 7.9 Hz, 1H), 7.86 – 7.60 (m, 4H), 7.56 (d, $J$ = 7.9 Hz, 1H), 7.33 – 7.27 (m, 2H), 7.12 – 7.01 (m, 3H), 7.00 – 6.92 (m, 1H), 4.51 – 4.37 (m, 2H), 4.28 (dd, $J$ = 8.3, 4.5 Hz, 1H), 4.21 – 4.06 (m, 1H), 3.71 – 3.58 (m, 1H), 3.57 – 3.46 (m, 1H), 3.14 (dd, $J$ = 14.8, 5.3 Hz, 1H), 2.99 (dd, $J$ = 14.8, 7.8 Hz, 1H), 2.78 – 2.67 (m, 2H), 2.15 – 2.03 (m, 2H), 2.03 – 1.91 (m, 1H), 1.91 – 1.68 (m, 3H), 1.68 – 1.39 (m, 6H), 1.38 – 1.13 (m, 29H), 0.85 (t, $J$ = 6.9 Hz, 3H). $^{13}$C NMR (126 MHz, DMSO-$d_6$): δ 173.15, 172.11, 171.79, 171.62, 170.33, 136.01, 127.42, 123.34, 120.82, 118.43, 118.21, 111.22, 109.97, 59.31, 52.99, 49.96, 48.74, 46.81, 38.67, 34.90, 31.27, 30.53, 29.04, 29.02, 28.99, 28.94, 28.76, 28.69, 28.66, 27.56, 26.66, 25.20, 24.44, 22.08, 21.92, 17.63, 13.94. LCMS (ESI): $m/z$ calcd for $C_{41}H_{68}N_7O_5^+$ [M + H]$^+$, 738.5; found 738.2.

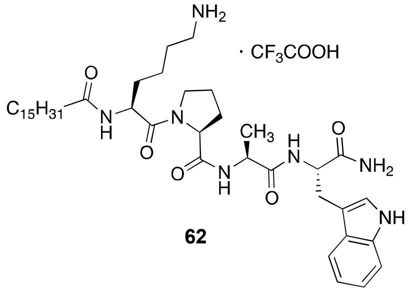


62

**Palmitoyl-Lys-Pro-His-Hph-NH2 TFA (63)** was synthesized according to the general procedure for SPPS, using MBHA Rink amide resin (1.0 g) and **palmitic acid** as R[1]-OH. After purification via RP-HPLC, compound **63** (42 mg, 10%) was obtained as a white powder. $^1$H NMR (500 MHz, $D_2O$): δ 7.24 – 7.29 (m, 1H), 7.09 – 7.19 (m, 4H), 7.02 – 7.07 (m, 1H), 4.69 (t, $J$ = 7.6 Hz, 1H), 4.44 – 4.50 (m, 1H), 4.37 (t, $J$ = 7.0 Hz, 1H), 4.25 (t, $J$ = 7.2 Hz, 1H), 3.50 – 3.71 (m, 2H), 3.19 – 3.37 (m, 2H), 2.87 – 2.97 (m, 2H), 2.65 – 2.75 (m, 1H), 2.53 – 2.63 (m, 1H), 1.86 – 2.21 (m, 6H), 1.41 – 1.84 (m, 8H), 1.03 – 1.36 (m, 27H), 0.84 (t, $J$ = 6.7 Hz, 3H). $^{13}$C NMR (126 MHz, $D_2O$): δ 175.83, 174.97, 173.73, 171.67, 171.26, 162.55 (q, $J$ = 35.2 Hz, TFA), 140.88, 133.63, 128.90, 128.43, 128.23, 125.82, 117.62, 116.46 (q, $J$ = 292.6 Hz, TFA), 60.79, 53.26, 52.58, 51.96, 47.58, 39.07, 35.38, 32.92, 31.86, 31.49, 30.06, 29.73, 29.70, 29.65, 29.61, 29.42, 29.34, 29.08, 26.50, 25.58, 24.72, 22.56, 21.93, 13.83. HRMS (ESI): $m/z$ calcd for $C_{43}H_{71}N_8O_5^+$ [M + H]$^+$, 779.5547; found 779.5532.

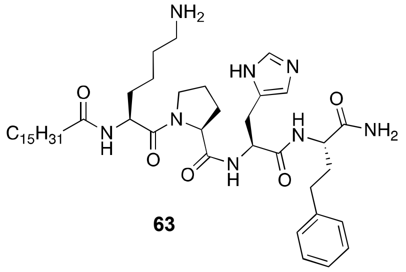


63

**Palmitoyl-Lys-Pro-Phg-Lys-NH2 bisTFA (64)**was synthesized according to the general procedure for SPPS, using MBHA Rink amide resin (0.75 g) and **palmitic acid** as R[1]-OH. After purification via RP-HPLC, compound **64** (121 mg, 40%) was obtained as a white powder. $^1$H NMR (500 MHz, $D_2O$): δ 7.20 – 7.52 (m, 5H), 5.53 (s, 1H), 4.38 – 4.53 (m, 2H), 4.25 (t, $J$ = 7.2 Hz, 1H), 3.75 (b, 1H), 3.51 (b, 1H), 2.84 – 2.98 (m, 4H), 2.19 (b, 3H), 1.57 – 1.98 (m, 11H), 1.34 – 1.49 (m, 6H), 1.13 – 1.32 (m, 25H), 0.87 (t, $J$ = 6.6 Hz, 3H). $^{13}$C NMR (126 MHz, $D_2O$): δ 171.38, 170.57, 168.78, 167.61, 166.83, 157.81 (q, $J$ = 35.2 Hz, TFA), 131.12, 124.22, 123.85, 122.90, 111.80 (q, J = 292.5 Hz, TFA), 56.29, 52.85, 49.09, 43.02, 34.41, 30.44, 27.23, 25.67, 25.12, 25.03, 24.97, 24.72, 24.55, 24.43, 21.82, 21.64, 20.70, 20.26, 17.93, 17.47, 9.18. HRMS (ESI): $m/z$ calcd for $C_{41}H_{71}N_7O_5Na^+$ [M + Na]$^+$, 764.5414; found 764.5431.

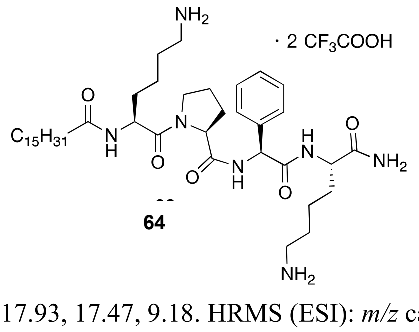


64

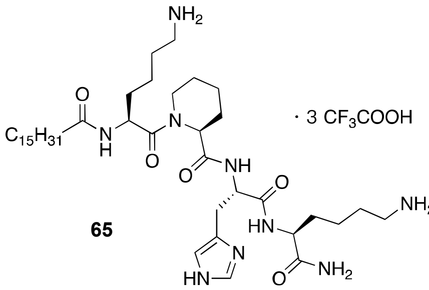

65

**Palmitoyl-Lys-Pip-His-Lys-NH2 bisTFA (65)**was synthesized according to the general procedure for SPPS, using MBHA Rink amide resin (0.75 g) and **palmitic acid** as R[1]-OH. After purification via RP-HPLC, compound **65** (23 mg, 5%) was obtained as a white powder. $^1$H NMR (500 MHz, $D_2O$): δ 8.51 – 8.66 (m, 1H), 7.30 (s, 1H), 5.05 (s, 1H), 4.26 – 4.40 (m, 2H), 3.90 (d, *J* = 13.0 Hz, 1H), 3.14 – 3.36 (m, 2H), 2.94 – 3.06 (m, 4H), 2.24 – 2.34 (m, 2H), 2.11 (d, *J* = 13.4 Hz, 1H), 1.57 – 1.89 (m, 12H), 1.38 – 1.51 (m, 4H), 1.28 (s, 24H), 0.87 (t, *J* = 6.7 Hz, 3H). HRMS (ESI): *m/z* calcd for $C_{40}H_{74}N_9O_5^+$ [M + H]$^+$: 760.5813; found 760.5840.

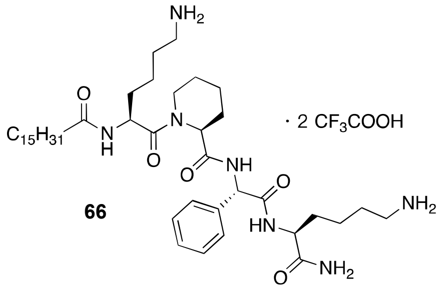

66

**Palmitoyl-Lys-Pip-Phg-Lys-NH2 bisTFA (66)** was synthesized according to the general procedure for SPPS, using MBHA Rink amide resin (0.75 g) and **palmitic acid** as R[1]-OH. After purification via RP-HPLC, compound **66** (33 mg, 8%) was obtained as a white powder. $^1$H NMR (500 MHz, $D_2O$): δ 7.15 – 7.59 (m, 5H), 5.58 – 5.64 (m, 1H), 5.20 (s, 1H), 4.24 – 4.34 (m, 1H), 2.18 (b, 3H), 1.09 – 1.85 (m, 43H), 0.83 (t, *J* = 6.7 Hz, 3H). $^{13}$C NMR (126 MHz, $D_2O$): δ 171.18, 170.87, 169.60, 168.08, 166.97, 166.44, 157.81 (q, *J* = 35.0 Hz, TFA), 131.72, 124.20, 123.82, 122.80, 122.49, 111.75 (d, *J* = 291.6 Hz, TFA), 52.35, 49.01, 48.71, 34.43, 34.37, 30.76, 27.14, 26.04, 25.67, 25.02, 24.94, 24.71, 24.62, 24.50, 24.00, 21.91, 21.64, 20.85, 17.84, 17.41, 17.34, 9.12. HRMS (ESI): *m/z* calcd for $C_{42}H_{74}N_7O_5^+$ [M + H]$^+$, 756.5751; found 756.5755.

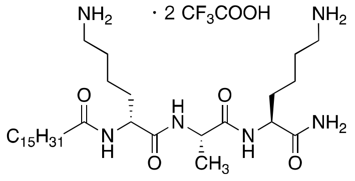

67

**Palmitoyl-D-Lys-Ala-Lys-NH2 bisTFA (67)** was synthesized according to the general procedure for SPPS, using MBHA Rink amide resin (0.30 g) and **palmitic acid** as R[1]-OH. After purification via RP-HPLC, compound **67** (72 mg, 45%) was obtained as a white powder. $^1$H NMR (400 MHz, MeOH-*d*4): δ 4.33 – 4.19 (m, 2H), 4.14 (t, *J* = 7.3 Hz, 1H), 2.93 (t, *J* = 7.6 Hz, 4H), 2.31 – 2.17 (m, 2H), 2.05 – 1.08 (m, 40H), 0.90 (t, *J* = 6.4 Hz, 3H). $^{13}$C NMR (101 MHz, MeOH-*d*4): δ 177.1, 177.0, 175.9, 175.4, 55.7, 54.6, 51.5, 40.61, 40.56, 36.6, 33.2, 31.9, 31.8, 30.95, 30.93, 30.90, 30.8, 30.63, 30.61, 30.59, 28.4, 28.0, 26.9, 24.2, 24.0, 23.9, 17.5, 14.6. HRMS (ESI): *m/z* calcd for $C_{31}H_{62}N_6O_4^+$ [M + H]$^+$ , 583.4905; found 583.4901.

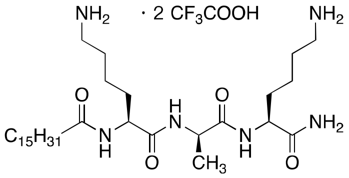

68

**Palmitoyl-Lys-D-Ala-Lys-NH2 bisTFA (68)** was synthesized according to the general procedure for SPPS, using MBHA Rink amide resin (0.30 g) and **palmitic acid** as R[1]-OH. After purification via RP-HPLC, compound **68** (75 mg, 46%) was obtained as a white powder.$^1$H NMR (400 MHz, MeOH-*d*4): δ 4.39 – 4.26 (m, 2H), 4.20 (dd, *J* = 8.1, 6.3 Hz, 1H), 2.93 (d, *J* = 7.4 Hz, 4H), 2.25 (td, *J* = 7.4, 2.8 Hz, 2H), 2.02 – 1.23 (m, 39H), 0.95 – 0.86 (m, 3H). $^{13}$C NMR (101 MHz, MeOH-*d*4): δ 177.0, 176.8, 175.2, 174.9, 163.2, 162.9, 55.4, 54.5, 51.0, 40.61, 40.57, 36.8, 33.2, 32.3, 32.1, 30.92, 30.91, 30.89, 30.88, 30.8, 30.63, 30.59, 30.5, 28.3, 28.00, 27.0, 23.94, 23.93, 23.86, 17.7, 14.6. HRMS (ESI): *m/z* calcd for $C_{31}H_{62}N_6O_4^+$ [M + Na]$^+$ , 605.4725; found 605.4728.

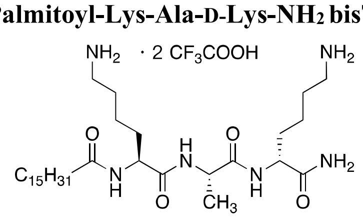

69

**Palmitoyl-Lys-Ala-D-Lys-NH2 bisTFA (69)** was synthesized according to the general procedure for SPPS, using MBHA Rink amide resin (0.30 g) and **palmitic acid** as R[1]-OH. After purification via RP-HPLC, compound **69** (90 mg, 56%) was obtained as a white powder. $^1$H NMR (400 MHz, MeOH-*d*4): δ 4.43 – 4.15 (m, 3H), 2.93 (td, *J* = 7.9, 2.5 Hz,41H), 2.25 (dd, *J* = 8.3, 6.8 Hz, 4H), 2.06 – 1.87 (m, 1H), 1.87 – 1.02 (m, 40H), 0.99 – 0.83 (m, 3H). $^{13}$C NMR (101 MHz, MeOH-*d*4): δ 177.0, 176.8, 175.5, 174.7, 54.8, 54.5, 51.1, 40.62, 40.58, 36.9, 33.2, 32.5, 32.2, 30.94, 30.92, 30.90, 30.81, 30.63, 30.61, 30.5, 28.3, 28.2, 27.0, 24.0, 23.9, 23.8, 17.5, 14.6. HRMS (ESI): *m/z* calcd for $C_{31}H_{62}N_6O_4^+$ [M + H]$^+$ , 583.4905; found 583.4896.

**Palmitoyl-D-Lys-Ala-Phe-NH2 TFA (70)** was synthesized according to the general procedure for SPPS, using MBHA Rink amide resin (0.50 g) and **palmitic acid** as R[1]-OH. After purification via RP-HPLC, compound **70** (43 mg, 20%) was obtained as a white powder. $^{1}$H NMR (500 MHz, DMSO-$d_4$): δ 7.33 – 7.23 (m, 4H), 7.21 – 7.17 (m, 1H), 4.87 (d, J = 2.0 Hz, 0H), 4.48 (dd, $J$ = 11.0, 4.3 Hz, 1H), 4.11 (t, $J$ = 7.30 Hz, 1H), 4.09 (q, $J$ = 7.33 Hz, 1H), 3.28 (dd, $J$ = 14.15, 4.34 Hz, 1H), 3.07 (dd, $J$ = 13.9, 11.0 Hz, 1H), 2.97 – 2.87 (m, 2H), 2.26 (t, $J$ = 7.6 Hz, 2H), 1.81 – 1.54 (m, 6H), 1.53 – 1.21 (m, 26H), 1.17 (d, $J$ = 7.3 Hz, 3H), 0.89 (t, $J$ = 7.0 Hz, 3H). $^{13}$C NMR (126 MHz, DMSO-$d_4$): δ 176.77, 176.50, 176.08, 174.96, 139.22, 130.25, 129.44, 127.69, 56.31, 55.68, 51.58, 40.48, 37.99, 36.35, 33.11, 31.59, 30.83, 30.81, 30.79, 30.66, 30.52, 30.51, 30.47, 28.38, 26.68, 23.81, 23.77, 17.12, 14.48. HRMS (ESI): *m/z* calcd for $C_{34}H_{60}N_5O_4^+$ $[M + H]^+$, 602.4640; found 602.4638.

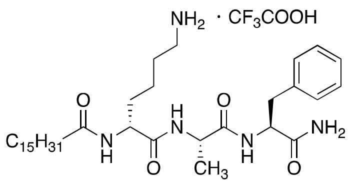
70

**Palmitoyl-D-Lys-Ala-Ala-Lys-NH2 bisTFA (71)** was synthesized according to the general procedure for SPPS, using MBHA Rink amide resin (0.30 g) and **palmitic acid** as R[1]-OH. After purification via RP-HPLC, compound **71** (77 mg, 42%) was obtained as a white powder. $^{1}$H NMR (400 MHz, MeOH-$d_4$): δ 4.36 – 4.20 (m, 3H), 4.17 (dd, $J$ = 8.1, 6.4 Hz, 1H), 2.93 (dtd, $J$ = 7.8, 5.7, 2.9 Hz, 4H), 2.15 – 2.27 (m, 2H), 1.98 – 1.11 (m, 43H), 0.95 – 0.85 (m, 3H). $^{13}$C NMR (101 MHz, MeOH-$d_4$): δ 176.8, 176.7, 175.6, 175.3, 175.2, 55.4, 54.1, 51.5, 51.2, 40.7, 40.6, 36.7, 33.2, 32.3, 32.0, 30.94, 30.93, 30.90, 30.8, 30.7, 30.6, 30.6, 28.4, 28.0, 27.0, 24.0, 23.9, 23.8, 17.6, 17.3, 14.6. HRMS (ESI): *m/z* calcd for $C_{34}H_{67}N_7O_5^+$ $[M + H]^+$ , 654.5276; found 654.5268.

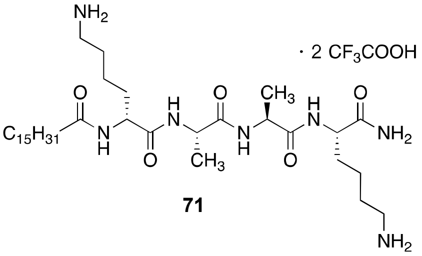
71

**Palmitoyl-Lys-D-Ala-Ala-Lys-NH2 bisTFA (72)** was synthesized according to the general procedure for SPPS, using MBHA Rink amide resin (0.30 g) and **palmitic acid** as R[1]-OH. After purification via RP-HPLC, compound **72** (83 mg, 53%) was obtained as a white powder. $^{1}$H NMR (400 MHz, MeOH-$d_4$): δ 4.39 – 4.20 (m, 4H), 3.02 – 2.84 (m, 4H), 2.27 (dd, $J$ = 8.3, 6.9 Hz, 2H), 2.03 – 1.55 (m, 10H), 1.55 – 1.17 (m, 34H), 0.97 – 0.84 (m, 3H). $^{13}$C NMR (101 MHz, MeOH-$d_4$): δ 177.0, 176.9, 175.3, 175.2, 174.4, 54.8, 54.3, 51.2, 50.8, 40.7, 40.6, 36.9, 33.2, 32.4, 32.3, 30.94, 30.93, 30.90, 30.8, 30.7, 30.6, 30.5, 28.6, 28.1, 27.0, 24.0, 23.89, 23.88, 17.6, 14.6. HRMS (ESI): *m/z* calcd for $C_{34}H_{67}N_7O_5^+$ $[M + H]^+$ , 654.5276; found 654.5275.

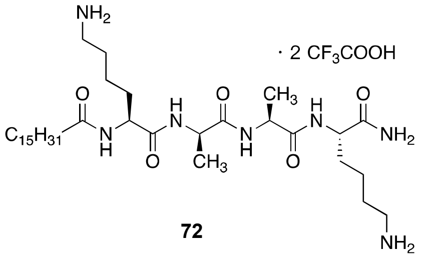
72

**Palmitoyl-Lys-Ala-D-Ala-Lys-NH2 bisTFA (73)** was synthesized according to the general procedure for SPPS, using MBHA Rink amide resin (0.30 g) and **palmitic acid** as R[1]-OH. After purification via RP-HPLC, compound **73** (83 mg, 45%) was obtained as a white powder. $^{1}$H NMR (400 MHz, MeOH-$d_4$): δ 4.39 – 4.20 (m, 4H), 3.02 – 2.84 (m, 4H), 2.27 (dd, $J$ = 8.3, 6.9 Hz, 2H), 2.03 – 1.55 (m, 10H), 1.55 – 1.17 (m, 34H), 0.97 – 0.84 (m, 3H). $^{13}$C NMR (101 MHz, MeOH-$d_4$): δ 177.0, 176.9, 175.3, 175.2, 174.4, 54.8, 54.3, 51.2, 50.8, 40.7, 40.6, 36.9, 33.2, 32.4, 32.3, 30.94, 30.93, 30.90, 30.8, 30.7, 30.6, 30.5, 28.6, 28.1, 27.0, 24.0, 23.89, 23.88, 17.6, 14.6. HRMS (ESI): *m/z* calcd for $C_{34}H_{67}N_7O_5^+$ $[M + H]^+$ , 654.5276; found 654.5275.

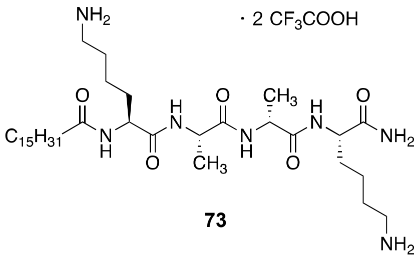
73

**Palmitoyl-D-Lys-Ala-Ala-Phe-NH2 TFA (74)** was synthesized according to the general procedure for SPPS, using MBHA Rink amide resin (1.0 g) and **palmitic acid** as R[1]-OH. After purification via RP-HPLC, compound **74** (190 mg, 40%) was obtained as a white powder. $^{1}$H NMR (500 MHz, MeOH-$d_4$): δ 7.28 – 7.23 (m, 4H), 7.22 – 7.16 (m, 2H), 4.54 (dd, $J$ = 9.2, 5.2 Hz, 1H), 4.23 (q, $J$ = 7.2 Hz, 1H), 4.20 – 4.15 (m, 2H), 3.23 (dd, $J$ = 14.1, 5.2 Hz, 0H), 2.98 – 2.89 (m, 3H), 2.25 – 2.10 (m, 2H), 1.88 – 1.80 (m, 1H), 1.77 – 1.63 (m, 3H), 1.61 – 1.39 (m, 4H), 1.37 (d, $J$ = 7.3 Hz, 3H), 1.35 – 1.19 (m, 26H), 0.92 – 0.87 (m, 3H). $^{13}$C NMR (126 MHz, MeOH-$d_4$): δ 176.56, 176.05, 175.46, 175.06, 174.95, 163.22, 162.94, 138.82, 130.28, 129.49, 127.74, 55.87, 55.19, 51.52, 51.31, 49.66, 49.49, 49.32, 49.15, 40.51, 38.55, 36.70, 33.11, 32.04, 30.83, 30.80, 30.78, 30.67, 30.51, 30.43, 28.31, 26.79, 23.85, 23.77, 17.51, 17.25, 14.47. HRMS (ESI): *m/z* calcd for $C_{37}H_{65}N_6O_5^+$ $[M + H]^+$, 673.5011; found 673.5004.

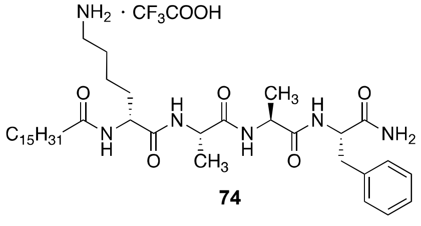
74

**Palmitoyl-Lys-Pro-Ala-D-Lys-NH2 bisTFA (75)** was synthesized according to the general procedure for SPPS, using MBHA Rink amide resin (1.0 g) and **palmitic acid** as R[1]-OH. After purification via RP-HPLC, compound **75** (62 mg, 13%) was obtained as a white powder. $^{1}$H NMR (500 MHz, DMSO-$d_4$): δ 8.12 (d, *J* = 6.9 Hz, 1H), 8.01 (d, *J* = 7.9 Hz, 1H), 7.83 (d, *J* = 8.3 Hz, 1H), 7.75 (s, 6H), 7.27 (d, *J* = 2.2 Hz, 1H), 7.06 (d, *J* = 2.1 Hz, 1H), 4.46 (td, *J* = 8.3, 5.3 Hz, 1H), 4.31 (dd, *J* = 8.3, 4.3 Hz, 1H), 4.24 – 4.15 (m, 1H), 4.10 (td, *J* = 8.9, 4.6 Hz, 1H), 3.70 – 3.60 (m, 1H), 3.56 – 3.47 (m, 1H), 2.75 (s, 4H), 2.09 (td, *J* = 7.3, 3.0 Hz, 2H), 2.06 – 1.99 (m, 1H), 1.93 – 1.76 (m, 3H), 1.76 – 1.67 (m, 1H), 1.66 – 1.41 (m, 9H), 1.38 – 1.15 (m, 32H), 0.87 – 0.83 (m, 3H). $^{13}$C NMR (126 MHz, DMSO-$d_4$): δ 173.54, 172.15, 172.12, 171.66, 170.41, 59.38, 52.06, 50.04, 48.53, 46.83, 38.68, 38.65, 34.90, 31.28, 31.07, 30.47, 29.04, 29.02, 29.00, 28.95, 28.77, 28.69, 28.67, 26.70, 26.56, 25.20, 24.44, 22.27, 22.08, 21.99, 17.64, 13.94. LCMS (ESI): *m/z* calcd for $C_{36}H_{70}N_7O_5^+$ $[M + H]^+$, 680.5; found 680.3.

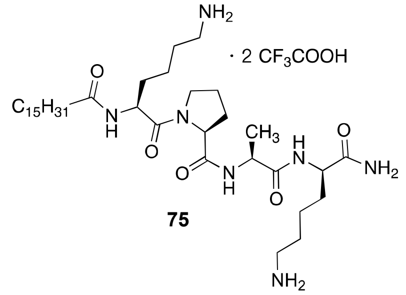

**75**

**Palmitoyl-Lys-Pro-Ala-D-Phe-NH2 TFA (76) Palmitoyl-Lys-Pro-Ala-Phe-NH2 TFA (78)** was synthesized according to the general procedure for SPPS, using MBHA Rink amide resin (0.30 g) and **palmitic acid** as R[1]-OH. After purification via RP-HPLC, compound **76** (21 mg, 8%) was obtained as a white powder. $^{1}$H NMR (400 MHz, DMSO-$d_6$): δ 8.07 (d, *J* = 7.6 Hz, 1H), 8.01 (d, *J* = 8.4 Hz, 1H), 7.96 (d, *J* = 8.8 Hz, 1H), 7.62 (br, 3H), 7.37 (s, 1H), 7.30 – 7.14 (m, 5H), 7.12 (s, 1H), 4.55 – 4.34 (m, 2H), 4.33 (m, 1H), 4.17 – 4.05 (m, 1H), 3.75 – 3.56 (m, 1H), 3.15 – 3.02 (m, 1H), 2.87 – 2.71 (m, 3H), 2.17 – 1.70 (m, 6H), 1.69 – 1.40 (m, 6H), 1.39 – 1.11 (m, 26H), 1.07 – 0.97 (m, 3H), 0.86 (t, *J* = 6.8 Hz, 3H). LCMS (ESI): *m/z* calcd for $C_{39}H_{67}N_6O_5^+$ $[M + H]^+$, 699.52; found 699.72.

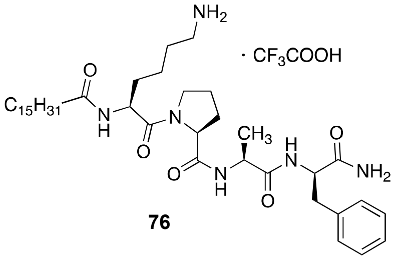

**76**

**Palmitoyl-Lys-Pro-Ala-D-Hph-NH2 TFA (77)** was synthesized according to the general procedure for SPPS, using MBHA Rink amide resin (0.50 g) and **palmitic acid** as R[1]-OH. After purification via RP-HPLC, compound **77** (55 mg, 35%) was obtained as a white powder. LCMS (ESI): *m/z* calcd for $C_{40}H_{69}N_6O_5^+$ $[M + H]^+$, 713.53; found 713.94.

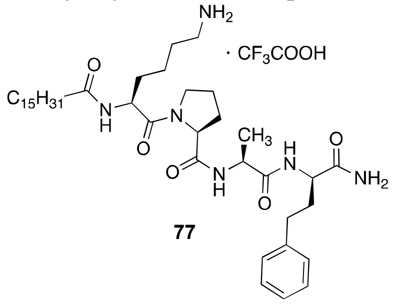

**77**

**Palmitoyl-Lys-Pro-Ala-D-Trp-NH2 TFA (78)** was synthesized according to the general procedure for SPPS, using MBHA Rink amide resin (0.50 g) and **palmitic acid** as R[1]-OH. After purification via RP-HPLC, compound **78** (19 mg, 7%) was obtained as a white powder. $^{1}$H NMR (500 MHz, DMSO-$d_4$): δ 10.80 (s, 1H), 8.10 (d, *J* = 7.2 Hz, 1H), 8.01 (d, *J* = 7.9 Hz, 1H), 7.87 (d, *J* = 8.2 Hz, 1H), 7.66 (s, 3H), 7.58 (d, *J* = 7.8 Hz, 1H), 7.37 – 7.28 (m, 2H), 7.13 – 7.01 (m, 3H), 6.99 – 6.93 (m, 1H), 4.50 – 4.44 (m, 1H), 4.39 (ddd, *J* = 9.7, 8.1, 4.3 Hz, 1H), 4.28 (dd, *J* = 8.3, 4.6 Hz, 1H), 4.16 – 4.08 (m, 1H), 3.69 – 3.62 (m, 1H), 3.53 – 3.47 (m, 1H), 3.18 (dd, *J* = 14.7, 4.3 Hz, 2H), 2.96 (dd, *J* = 14.7, 9.7 Hz, 2H), 2.84 – 2.65 (m, 3H), 2.15 – 1.96 (m, 4H), 1.92 – 1.71 (m, 4H), 1.69 – 1.39 (m, 7H), 1.22 (d, *J* = 7.5 Hz, 24H), 1.06 (d, *J* = 7.1 Hz, 3H), 0.89 – 0.81 (m, 3H). LCMS (ESI): *m/z* calcd for $C_{41}H_{68}N_7O_5^+$ $[M + H]^+$, 738.53; found 738.63.

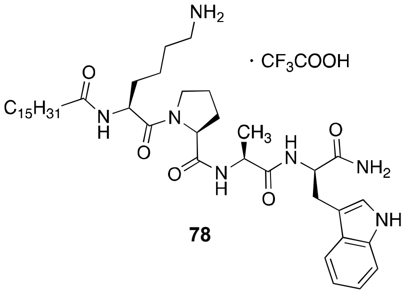

**78**

**Palmitoyl-D-Lys-Pro-Ala-Trp-NH2 TFA (79)** was synthesized according to the general procedure for SPPS, using MBHA Rink amide resin (1.0 g) and **palmitic acid** as $R^1$-OH. After purification via RP-HPLC, compound **79** (40 mg, 8%) was obtained as a white powder.

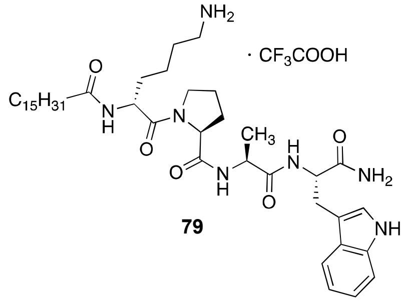


**79**

$^1$H NMR (500 MHz, MeOH-$d_4$): δ 7.64 – 7.57 (m, 1H), 7.38 – 7.29 (m, 1H), 7.15 – 7.07 (m, 2H), 7.05 – 6.99 (m, 1H), 4.66 – 4.60 (m, 1H), 4.41 (dd, J = 7.9, 6.2 Hz, 1H), 4.34 (dd, J = 8.5, 5.0 Hz, 1H), 4.25 – 4.13 (m, 1H), 3.89 – 3.80 (m, 1H), 3.62 (dt, J = 10.0, 6.9 Hz, 1H), 3.46 – 3.33 (m, 2H), 3.16 (dd, J = 15.0, 8.7 Hz, 1H), 2.90 (t, J = 7.7 Hz, 2H), 2.24 – 2.13 (m, 1H), 2.11 – 1.77 (m, 5H), 1.75 – 0.97 (m, 33H), 0.93 – 0.86 (m, 3H). $^{13}$C NMR (126 MHz, MeOH-$d_4$): δ 176.81, 176.50, 175.14, 174.96, 173.53, 138.11, 128.82, 124.19, 122.52, 119.86, 119.40, 112.38, 111.48, 62.63, 55.34, 53.17, 51.68, 49.32, 40.49, 36.34, 33.11, 31.69, 30.84, 30.82, 30.80, 30.76, 30.69, 30.65, 30.51, 30.49, 30.46, 30.37, 30.35, 30.32, 28.73, 28.39, 26.70, 25.80, 23.77, 23.66, 17.00, 14.48. LCMS (ESI): *m/z* calcd for $C_{41}H_{68}N_7O_5^+$ [M + H]$^+$, 738.53; found 738.48.

## Biology

### Biochemical Protease Assays

Single dose screening of compounds and/or $IC_{50}$ determination was performed by a fluorimetric assay for DENV protease as described before.[1, 2] The FRET substrate comprised the sequence 2-Abz-Nle-Lys-Arg-Arg-Ser-(3-$NO_2$)-Tyr-$NH_2$ ($K_m$ = 105 $\mu$M). By catalytic activity of the protease, the cleaved substrate results in increased fluorescence that can be monitored using a BMG Labtech Fluostar OPTIMA Microtiter fluorescence plate reader (excitation wavelength of 320 nm and a monitored emission wavelength of 405 nm). Dilutions were made starting from 10 mM stock solutions in DMSO and final concentrations were measured in triplicates. The inhibitors were preincubated for 15 min with the DENV protease (100 nM) in the assay buffer (50 mM Tris-HCl pH 9.0, ethylene glycol (10% v/v), and 0.0016% Brij 58). The enzymatic reaction was initiated by the addition of the FRET substrate (final concentration 50 µM) to obtain a final assay volume of 100 µL per well. The enzymatic activity was monitored for 15 min and determined as a slope of relative fluorescence units per second (RFU/s) for each concentration. The mean of the triplicates were plotted against the corresponding concentration in CDDVault to determine the $IC_{50}$-values. Analogous procedures were used to determine the inhibitory potency on WNV[3] and ZIKV protease.[4] For selectivity screening of active candidates, we used similar biochemical assays with human serine proteases trypsin[5] and/or thrombin[2].

### Cellular Viral Infection Assays

**Protocol 1:**

**<u>LLC-MK2 Cells: DENV2 IPOX Assay</u>**

***Cell Preparation*** **– LLC-MK2 (Monkey Rhesus Kidney cells; CCL-7.1)** were passaged in assay medium (EMEM (Lonza Cat No: BESP069F) supplemented with 10% heat-inactivated FCS (Lonza), 2% Pen/strep (Gibco), 2% L-Gln (Gibco), 2% Hepes (Lonza), 1% $NaHCO_3$ (Lonza)) prior to use in the antiviral assay. Cells were seeded in 96-wells plates ($10^5$ cells/well) in assay medium to be exposed 16-24 h later to compounds and virus. The plates were incubated at 37 °C / 5% $CO_2$ overnight to allow for cell adherence.

***Compound Preparation*** – Compounds were solubilized in DMSO and evaluated using two-fold serial dilutions (8-points dose-response curves starting at a concentration of 50 µM) in duplicate for the antiviral assays. Compounds were diluted in assay medium at 1X test concentrations. Ribavirin (Sigma Aldrich) was evaluated as a positive control compound in the antiviral assays.

***Virus Preparation and Cellular Infection*** - DENV2 New Guinea strain was grown in AP-61 insect cells (in-house cell bank) in complete Leibovitz medium containing 1% pen/strep (Gibco), 1% L-Gln (Gibco), 0.5% Hepes (Lonza), 0.5% $NaHCO_3$ (Lonza) and 10% Tryptose phosphate for the production of stock virus pools. On the day of cellular infection, an aliquot of virus was removed from the freezer (-80°C) and allowed to thaw in water in a biological safety cabinet. Virus was diluted into assay medium ($10^4$ $TCID_{50}$), and 100 µL of this was added to each well, resulting in a $TCID_{50}$ of 100. Cells were incubated for 2 h at 37 °C / 5% $CO_2$ and washed 3 times with blank assay medium. Directly after washing, 100 µl of the compound dilutions were added to each well.

***Plate Format*** – Each plate contained cell control wells (cells only), virus control wells (cells plus virus), duplicate drug toxicity wells per compound (cells plus drug only), as well as duplicate experimental wells (drug plus cells plus virus).

***Immunoperoxidase Staining and Toxicity Determination*** – Virus infected cells were visualized using a DENV2 immunoperoxidase staining protocol. Two days after infection, cells were inactivated with ethanol 70% for 30 min and washed with PBS. Fixed plates were incubated with PBS containing 0.05% $H_2O_2$ for 20 min at 37 °C and washed again 3 times with PBS. Plates were incubated for 1 h with 50 µl monoclonal anti-DENV-2 NS1 antibody (Milipore; diluted 1:500 in EMEM). Samples were washed once with PBS containing 0.05% Tween20, and twice with PBS only. Secondary polyclonal goat anti mouse IgG HRP (Dako; diluted 1:2000) was added 50 µl per well and incubated for 1hr at 37 °C in the dark. Following 3 washing steps with PBS, 100 µl AEC (3-Amino-9-Ethylcarbazole) substrate buffer (containing 0.03% hydrogen peroxide, 3% DMF) was added to each well and incubated for 30 min at room temperature in the dark. Bidest water was added after removal of the substrate solution, and all virus positive cells per well (marked by brown/red staining) were counted under a microscope. Visual scoring of toxicity per well was performed in parallel.

***Data Analysis*** – First, the numbers of infected cells in duplicate wells were averaged. Subsequently, the average of compound plus virus treated wells was normalized against the average of DMSO plus virus treated wells to calculate percentage inhibition. Processed dose-response data were uploaded in CDD Vault,[6] delivering $EC_{50}$ values for each compound. Qualitative toxicity profiles were uploaded in parallel.

**Vero Cells:**

***Antiviral assay –*** Vero cells were plated in DMEM containing 10% FBS in 96-wells plates at a concentration of 1.5 × $10^4$/well and incubated at 37 °C for 12-16 h, until ~80% confluency was reached. Next day, DENV-2 was diluted to a working concentration of 10,000 TCID50/mL in DMEM containing 2% FBS, resulting in 1000 TCID50 per well. 100µl of the diluted virus was added to the respective wells (except the cell control) and the plates were incubated at 37 °C / 5% $CO_2$ for 1 h and 30 min (also known as virus adsorption phase). During the time of virus adsorption phase, the compounds were diluted to 50 µM from a stock concentration of 10mM, and subsequently titrated in a eight-step, two-fold serial dilution up to a dilution of 0.4 µM (each compound was tested in duplicate). Each plate also contained a positive control (PC), negative control (NC) (both PC and NC were diluted 2-fold, four step dilution starting concentration 25 µM) and virus control (VC) (eight replicates) to which only virus was added. The virus was not added to the cell control (CC) (eight replicates). Once the adsorption phase was over, the wells were washed twice with PBS, and incubated for approx. 48 h (± 2 h) at 37° C / 5%. After incubation, the wells were fixed with 2.5% formalin for 15 min at room temperature (RT), followed by complete inactivation of the plate with 80% MeOH for 10 min. For the staining of the plate, wells were first washed once with PBS and subsequently permeabilized using 0.1% Triton-X100 in 70% ethanol for 30 min at room temperature. The cells were washed once with PBS. Then the wells were emptied and incubated with Dengue virus-2 NS3 polyclonal primary antibody (Sigma; 1:1500) in dilution buffer (PBS containing 0.5% BSA), for 1 h at 37 °C. Cells were then washed twice with PBS/0.5% Tween-20 and once with PBS, followed by incubation with goat anti-rabbit IgG (H+L) Alexa Fluor 488 antibody (Invitrogen; 1:7000) for 1 h at 37 °C. Cells were washed twice with PBS. Following this, wells were incubated with 4',6-Diamidino-2-Phenylindole, Dihydrochloride (DAPI) (Invitrogen; 1:6000) (nuclear counterstain) diluted in PBS for 15 min at room temperature, and then washed twice with PBS. Plates were read with the Cytation1V Imaging Reader (BioTek) at a 4× objective and analyzed by the Gen5 software (BioTek).

***Cellular cytotoxicity assay –*** In vitro cytotoxicity of compounds (starting at 50 µM) was tested by assessing cellular metabolic activity using an MTT assay. Briefly, Vero cells were plated in DMEM containing 10% FBS in 96-well plates at a concentration of 1.5 × $10^4$/well and incubated at 37 °C for 12-16 h, until ~80% confluency was reached (also known as substrate plate). The next day, compounds were diluted (in a round bottom 96-well plate) to 50 µM from a stock concentration of 10mM, and subsequently titrated in an eight-step, two-fold serial dilution up to a dilution of 0.4 µM (each compound was tested in duplicate). Each plate also contained a cell control (at least sixteen replicates each plate), in which no compounds were added. The diluted compounds were transferred to the substrate plate and incubated for approx. 48 h (± 2 h) at 37 °C / 5%. Once the incubation time was over, the plates were washed twice with PBS. 100 µl of DMEM was added to each well followed by the addition of 10% MTT solution of total volume. The plates were incubated for approximately 4 h at 37 °C / 5% $CO_2$, resulting in formation of formazan crystals. Once the crystals were clearly visible, 100 µl solubilization solution was added to each well to dissolve the crystals. The plates were incubated for approximately 16 h at 37 °C / 5% $CO_2$. Finally, the absorbance of each well was detected at 570 nm wavelength with a reference wavelength higher than 650 nm using iMark™ Microplate Absorbance Reader. Based on the obtained data, the percentage viability was calculated.

***Data analysis and calculations –*** For the antiviral assay, the fluorescent immunostaining (DAPI and Alexa Fluor 488) was captured by the Cytation1V Imager DAPI and GFP channels and processed and analyzed by the Gen5 software. In

the software, each image of the well was divided into 12 sections (4 × 3) and cropped to the size of the well. During processing, the images were stitched, and the edge of the wells (due to the effect of auto-fluorescence) excluded from the analyses. The cell counts for Alexa Fluor 488 and DAPI were automatically generated by the software for each entire plate.

The antiviral activity of the compounds against DENV-2 was determined by calculating the inhibition percentage of each compound at each dilution and represents the reduction in the number of infected cells relative to the virus control. An Excel calculator template was used to calculate the inhibition percentage using the number of infected cells. The following equation was used:

$$\text{\% Inhibition} = \left[ \frac{\text{(Average Number of infected cells in virus control - Average mumber of infected cells in specific dilution)}}{\text{Average Number of infected cells in virus control}} \right] \text{X} \quad 100$$

Data normalization was performed considering any value below "0" was considered as "0" and any value above "100" was considered as "100".

For the cellular cytotoxicity assay, the optical density of each plate was measured using iMark™ Microplate Absorbance Reader, in a dual mode at a wavelength of 570 nm, with a reference wavelength above 650 nm. To determine the percentage of viable cells the following formula was used:

Percent Viability = (O.D of treated well)/Average O.D of Cell control and DMSO control)*100

Followed by determination of Fifty percent cytotoxic concentration ($CC_{50}$) which may be defined as the extract concentration that reduced the cell viability by 50% when compared to untreated controls.

**Protocol 2: Anti-Dengue Virus Cytoprotection Assay**

***Cell Preparation*** – BHK21 cells (Syrian hamster kidney cells; ATCC catalog CCL-10) were passaged in DMEM supplemented with 10% FBS, 2 mM L-Gln, 100 U/mL penicillin, and 100 µg/mL streptomycin in T-75 flasks prior to use in the antiviral assay. On the day preceding the assay, the cells were split 1 : 2 to assure they were in an exponential growth phase at the time of infection. Total cell and viability quantification was performed using a hemocytometer and Trypan Blue dye exclusion. Cell viability was confirmed to be greater than 95% for the cells to be utilized in the assay. The cells were resuspended at $5 \times 10^3$ cells per well in tissue culture medium and added to flat bottom microtiter plates in a volume of 100 µL. The plates were incubated at 37 °C / 5% $CO_2$ overnight to allow for cell adherence.

***Compound Preparation*** – Compounds were solubilized in DMSO and evaluated using single doses and/or five serial half-logarithmic dilutions (starting at a concentration of 50 µM) in triplicate for the antiviral assays. Compounds were diluted in assay medium (DMEM supplemented with 2% heat-inactivated FBS, 2 mM L-Gln, 100 U/mL penicillin, and 100 µg/mL streptomycin) at 2X test concentrations and 100 µL per well transferred to the 96 well plate following removal of the medium from the cell monolayers. Ribavirin (Sigma Aldrich) was evaluated as a positive control compound in the antiviral assays.

***Virus Preparation*** – DENV2 New Guinea strain was previously obtained from ATCC (VR-1584) and was grown in LLC-MK2 (Rhesus monkey kidney cells, CCL-7.1) cells for the production of stock virus pools. On the day of cellular infection, an aliquot of virus was removed from the freezer (-80ºC) and allowed to thaw in water in a biological safety cabinet. Virus was diluted into assay medium such that the amount of virus added to each well in a volume of 100 µL was the amount determined to yield 85 to 95% cell killing at 6 days post-infection.

***Plate Format*** – Each plate contained cell control wells (cells only), virus control wells (cells plus virus), duplicate drug toxicity wells per compound (cells plus drug only), as well as triplicate experimental wells (drug plus cells plus virus).

***Efficacy and Toxicity XTT*** - Following incubation at 37ºC in a 5% $CO_2$ incubator, the test plates were stained with the tetrazolium dye XTT (2,3-bis(2-methoxy-4-nitro-5-sulfophenyl)-5-[(phenylamino)carbonyl]-2H-tetrazolium hydroxide).

XTT-tetrazolium is known to be metabolized by the mitochondrial enzymes of metabolically active cells to a soluble formazan product, allowing rapid quantitative analysis of the inhibition of virus-induced cell killing by antiviral test substances. XTT solution was prepared daily as a stock of 1 mg/mL in RPMI1640. Phenazine methosulfate (PMS) solution was prepared at 0.15 mg/mL in PBS and stored in the dark at -20 °C. XTT / PMS stock was prepared immediately before use by adding 40 µL of PMS per mL of XTT solution. Fifty microliters of XTT/PMS was added to each well of the plate and the plate was reincubated for 4 h at 37 °C. Plates were sealed with adhesive plate sealers and shaken gently or inverted several times to mix the soluble formazan product and the plate was read spectrophotometrically at 450 / 650 nm with a Molecular Devices Vmax plate reader.

***Data Analysis*** - The raw data were collected from Softmax Pro and imported into a Microsoft Excel spreadsheet for analysis. Mean optical density values at each compound concentration were first corrected for corresponding colorimetric wells (compound in media only). Averaged data of compound wells (+virus) were normalized against DMSO (+virus) treated wells to calculate inhibition at each tested compound dose as percentage of reduction for viral cytopathic effect (CPE). Data of compound wells (-virus) were normalized against DMSO (-virus) treated wells to calculate toxicity at each tested compound dose. Processed dose-response data were uploaded in CDD Vault,[6] delivering $EC_{50}$ and $CC_{50}$ values.

**Protocol 3: WNV and ZIKV antiviral assay**

***Virus preparation*** – All experiments containing the active WNV and ZIKV were performed at Fraunhofer Institute for Cell Therapy and Immunology, Leipzig in BSL-3 and BSL-2 facilities, respectively. Vero E6 cells were grown in T175 flasks to a confluence of approx. 80-90% and were infected at a multiplicity of infection (MOI) of 0.01 focus forming units (ffu) per cell in 5 mL serum free Dulbecco's modified Eagle's medium (DMEM). After 1 h at 37 °C, 20 ml of DMEM with 2 % FCS were added and cells were incubated for 30 h or 48-72 h at 37 °C with 5% $CO_2$ for WNV (isolate WNV New York 2000 – crow 3356 AF404756.1, kindly provided by Jonas Schmidt-Chanasit, Bernhard Nocht Institute for Tropical Medicine, Hamburg) and ZIKV (isolate Dominican Republic/2016/PD1, kindly provided by Luisa Barzon, University of Padova) culture, respectively until cytopathic effect was visible. Virus containing supernatant was centrifuged at 4000 *g* for 10 min at 4 °C and stored in 0.5 ml aliquots at -80 °C. To determine the viral titers, virus containing supernatants were serially diluted before addition to confluent Vero E6 monolayers in 96-well plates. After an incubation of 1 h at 37 °C, supernatant was removed and cells were overlaid with 1.2 % methyl cellulose in DMEM with 2% FCS and incubated for 24-30 h at 37 °C in 5% $CO_2$. Cells were then fixed with 4% paraformaldehyde in PBS for 15-30 min at room temperature, permeabilized and blocked with perm-wash buffer (0.1% saponin, 0.1% BSA in PBS). WNV and ZIKV ffu were stained using a monoclonal mouse anti fusion-loop antibody (4G2, absolute antibody) at 1:2,000 dilution and a secondary rabbit anti-mouse IgG HRP-conjugated antibody (Dako) at 1:2,000 dilution in perm-wash buffer. After the addition of TrueBlue substrate (Seracare), spots were automatically counted with an ELISpot reader (AID Diagnostika).

***Compounds preparation and testing*** – Compounds were prepared in serial 2-fold dilutions in DMEM without FCS and mixed with 50-150 focus forming units of WNV and ZIKV, resulting in final compound concentrations of 50 µM to 0.39 µM. The same dilutions were prepared with DMSO, which served as a control. Virus-compound mixtures were then added to confluent Vero E6 cell monolayers in 96-well plates. After an incubation at 37 °C for 1 h, the supernatants were removed, cells were washed with PBS and subsequently overlaid with serially diluted compounds (50 µM-0.39 µM) in DMEM with 2 % FCS and 1.2% Avicel. Cells were washed twice with PBS after an incubation of 24-30 h at 37 °C in 5 % $CO_2$, fixed with 4% paraformaldehyde in PBS for 15-30 min at room temperature, permeabilized and blocked with perm-wash buffer (0.1% saponin, 0.1% BSA in PBS). WNV and ZIKV ffu were stained using a monoclonal mouse anti fusion-loop antibody (4G2, absolute antibody) at 1:2,000 dilution and a secondary rabbit anti-mouse HRP-conjugated antibody (Dako) at 1:2,000 dilution in perm-wash buffer. After the addition of TrueBlue substrate (Seracare), spots were automatically counted with an ELISpot reader (AID Diagnostika). WNV and ZIKV ffu were normalized to the DMSO control from the corresponding dilutions and means of two to three independent experiments were used to calculate $EC_{50}$ values with GraphPad Prism and a dose-response function with a variable slope. Processed dose-response data were uploaded in CDD Vault,[6] delivering $EC_{50}$ values for each compound. Qualitative toxicity profiles were uploaded in parallel.

**In vivo PK**

All animal studies were performed with the approval of and under the guidelines of the ethics committee of the test facility, see S48 for Certificate. The pharmacokinetic profile of **73** was investigated in female C57BL6 (N = 3 per group).

Animals were kept fasting 10-12 h prior to dosing, feeding was allowed 2 h post dose. For IV, IP and SC administration 5 mL / kg of solution formulation was administered. For IV administration animals (N = 3) were dosed 5 mg / kg through tail vein, using 27-gauze needle, with 1 mg / mL of **73** formulated in 20% aqueous HPβCD + 5% DMSO. For IP (using 27-gauze needle, N= 3) and SC (through gastric gauge, N= 3) administration animals were dosed 10 mg / kg with 2 mg / mL of **73** formulated in 20% aqueous HPβCD. An aliquot of the formulation solution was taken before the dosing begins and at the end of dosing. The dosing solution was immediately diluted with a suitable solvent and stored at approximately –20 °C or below for subsequent LCMS analysis to disprove eventual degradation of the test compounds in the formulation throughout the study. Mice were anesthetized using gaseous anaesthesia and lateral saphenous vein was used for sampling while taking aseptic precautions. The back of the hind leg was shaved until saphenous vein was visible. The animal was restrained, hind limb was immobilized and slight pressure was applied gently above the knee joint. The vein was punctured using a 20-gauge needle and 30 µL of blood was collected in pre-labelled pre-chilled tubes. Blood samples were collected 0.083, 0.25, 1, 2, 4, 8 and 24 h after IV administration, and 0.25, 1, 2, 4, 6, 8 and 24 h after IP, SC and PO administration. After collection of blood samples at each time point,[2] the blood samples were stored on ice, prior to centrifugation. The blood samples were centrifuged within 0.5 hour of collection to separate plasma, at 2500 x g for 15 min at 4 °C and plasma was stored at –20 °C. 25 µL of plasma sample was precipitated with 200 µL of acetonitrile containing the internal standard (Telmisartan 200 ng / mL) then Vortexed for 5 min at 850 rpm, centrifuged at 4000 rpm for 5 min at 4 °C. 110 µL of supernatant was separated and diluted with 130 µL of 1 : 1 = methanol / water and submitted to LC-MS / MS analysis (LC: Analytical UPLC Agilent 1260 infinity; column: Phenomenex, Kinetex EVO C18 100A 50 mm x 4.6 mm, 5 µm; flow rate: 1.00 mL / min; solvents: A, 10 mM aqueous ammonium acetate + 0.1% aqueous formic acid; B, methanol; gradient: 90% to 10%A in 1.0 min, 10%A for 1.4 min, 10% to 90%A in 0.1 min, 90%A for 1.1 min; MS: API 5500+; Rt, ion transitions monitored, DP and CE: **73**, 1.47 min, m / z Q1 327.80, Q3 129.10, 90 V and 55 eV; Telmisartan, 1.67 min, m / z Q1 515.30, Q2 276.20, 60 V, 45 eV; MS parameters: CXP, 10; CAD, 9; CUR, 45; GS1, 50; GS2, 55; TEM, 550; ISV, 5500; IHE, ON). Total ion chromatogram for each compound compared to the internal standard and calibration curve was used for quantitation. Pharmacokinetic parameters were calculated by non-compartmental analysis (NCA) using sparse sampling approach with Phoenix software version 8.1.

[2] Time windows for blood collection ±0.50 min for 0.033 h, ±2 min for 0.25 h and 0.5 h, ±5 min for 1 to 8 hours and ±15 min for 24 h were not considered as deviation.

## Supplementary Data

**Comparative view amongst the DENV2 antiviral cellular assays used in this work.**

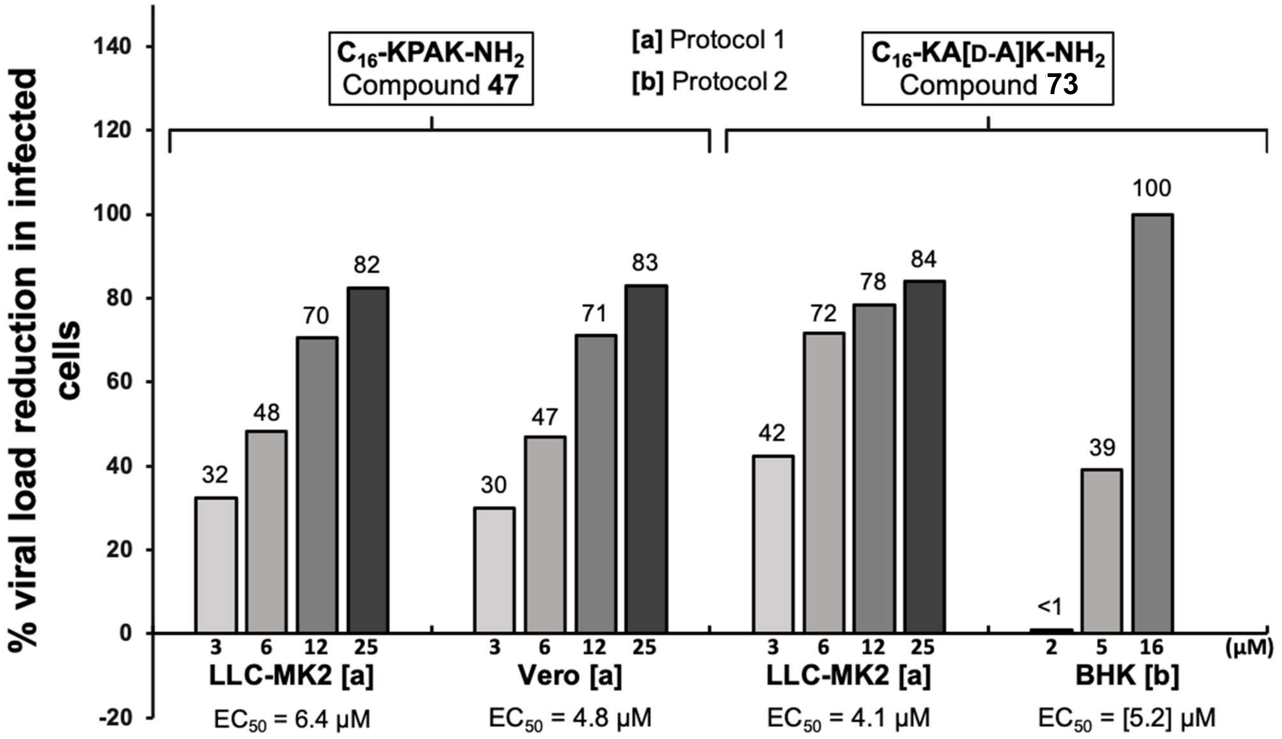


**Figure S1. Antiviral efficacy of 47 and 73 across the different cell lines and protocols used in this work. Full DRCs are reported below.**

As shown in Figure S1 the antiviral efficacy of lipopeptide **47** through Protocol 1 is conserved using either LLC-MK2 or Vero cells, obtaining comparable % of viral load reduction in the significant (3-25 µM) concentration range. The antiviral efficacy of lipopeptide **73** is conserved through Protocol 1, using LLC-MK2 cells, and Protocol 2, using BHK cells. The $EC_{50}$ reported in brackets means not enough points have been collected to determine an appropriate DRC. The apparent disparity between % of viral load reduction per analogous concentration point in LLC-MK2 and BHK cells is due to the different reporter systems used in Protocol 1 and Protocol 2. Through Protocol 1 the viral load reduction was obtained via immunocytochemistry, measuring the residual virus in treated infected cells compared to untreated infected cells. Through Protocol 2 the viral load reduction was measured as the residual living infected cells amongst treated cells, compared to untreated infected cells. The difference in recording methods caused a sharper increase in viral load reduction through Protocol 2 than in Protocol 1; nonetheless the $EC_{50}$ values found were in the same concentration range. Lipopeptides **47** and **73** did not show significant toxicity in the relevant (3-25 µM) concentration range amongst the cell lines and protocols.

| 47 | Protocol 1, LLC-MK2 Cells | 47 | Protocol 1, Vero Cells |
|---|---|---|---|

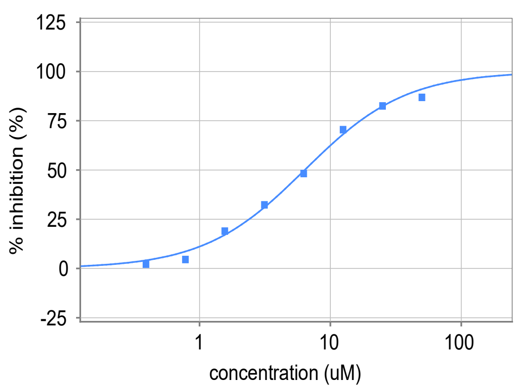


EC$_{50}$ = 6.4 µM

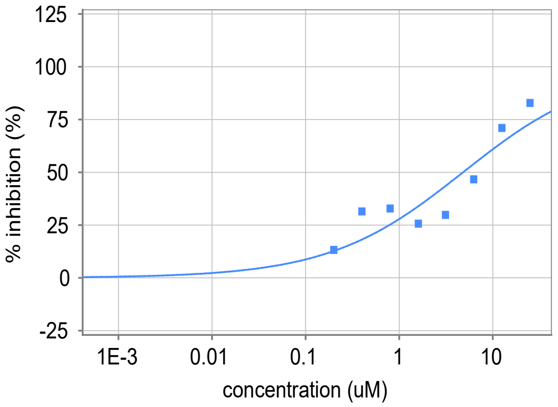


EC$_{50}$ = 4.8 µM

| 73 | Protocol 1, LLC-MK cells | 73 | Protocol 2, BHK cells |
|---|---|---|---|

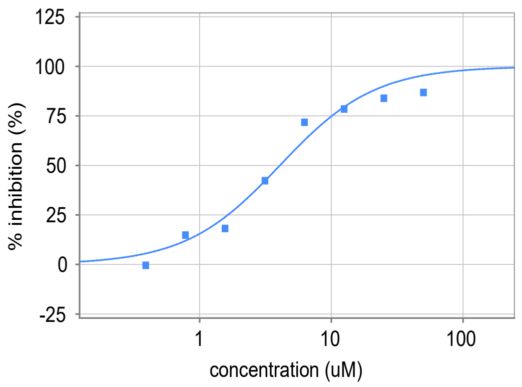


EC$_{50}$ = 4.1 µM

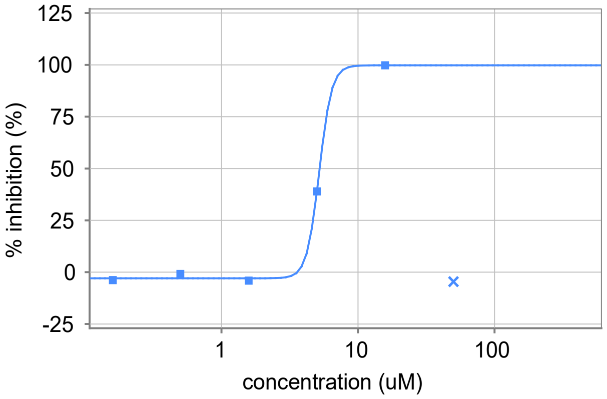


EC$_{50}$ = 5.2 µM

**Figure S2. DRCs of the cellular antiviral efficacy of lipopeptides 47 and 73 across the different cell lines and protocols used in this work.**

## Dose Response Curves (DRCs)

### Library I

### NS2B-NS3 inhibition

**37**

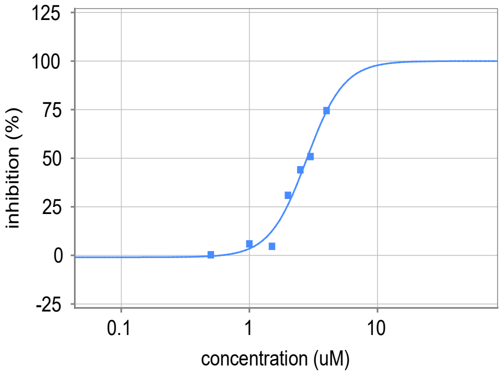


**34**

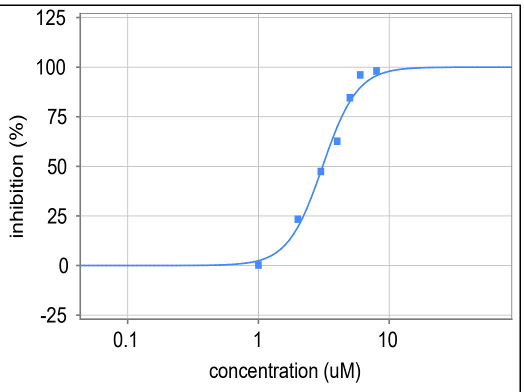


**38**

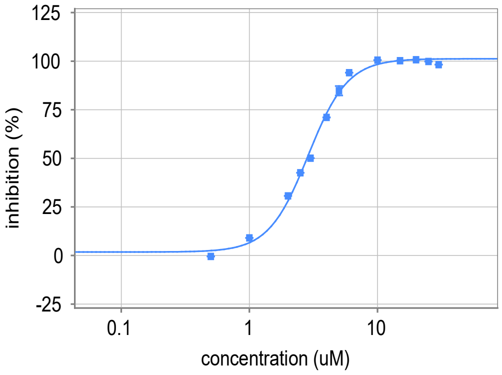


**35**

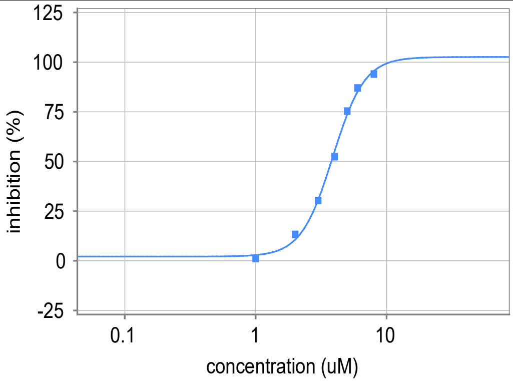


**39**

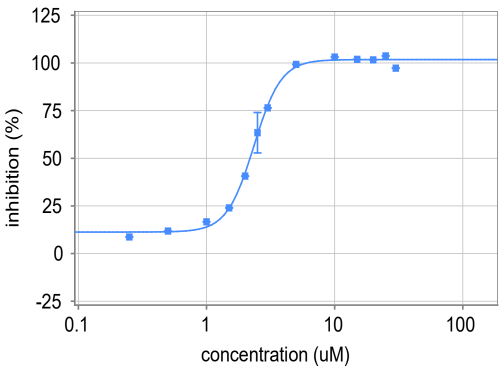


**36**

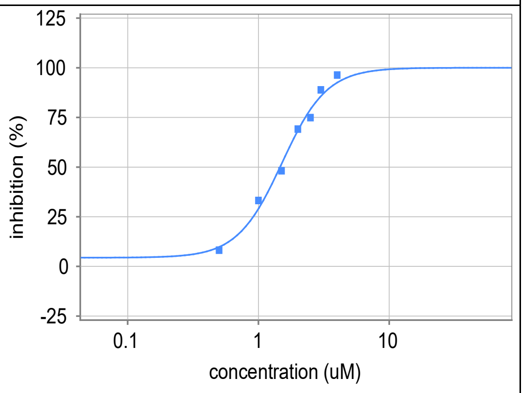

**DENV2 cellular infection DRC**

**37** Protocol 1, LLC-MK cells

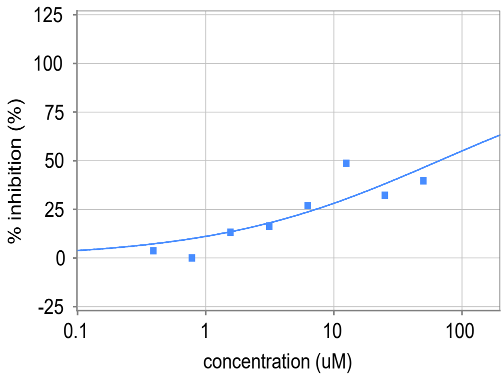


**34** Protocol 1, LLC-MK cells

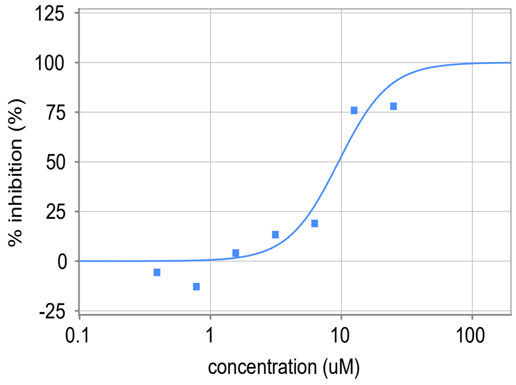


**38** Protocol 1, LLC-MK cells

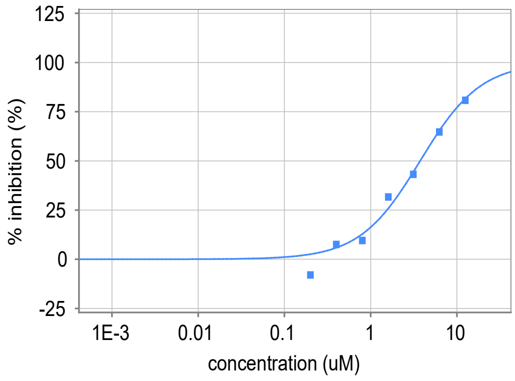


**35** Protocol 1, LLC-MK cells

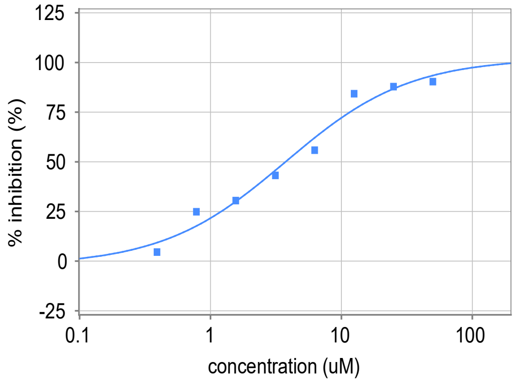


**39** Protocol 1, LLC-MK cells

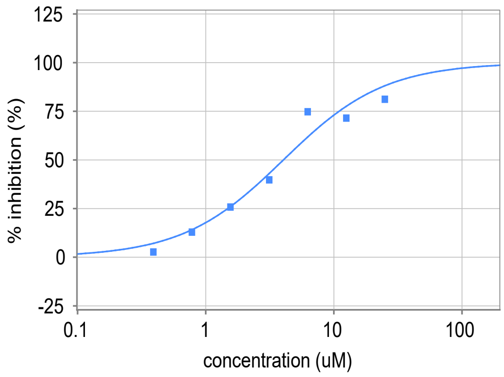


**36** Protocol 1, LLC-MK cells

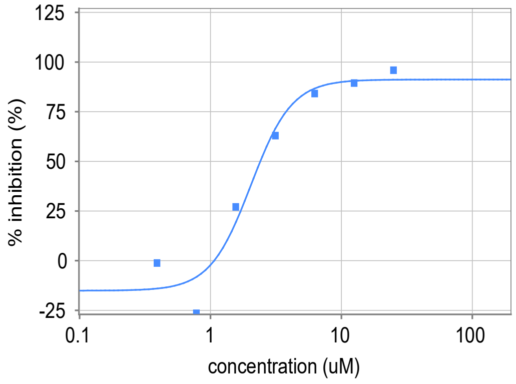

## Viability

**Table S 1. Observed cellular toxicity for compounds in Library I per concentration point.[a]**

| Handling code | Concentration (µM) | Tox | cells |
|---|---|---|---|
| **34** | 50 | TT | LLC-MK2 |
| | 25 | T | LLC-MK2 |
| **38** | 50 | TTT | LLC-MK2 |
| | 25 | TT | LLC-MK2 |
| | 12.5 | T | LLC-MK2 |
| **39** | 50 | TTT | LLC-MK2 |
| | 25 | T | LLC-MK2 |
| **36** | 50 | TTT | LLC-MK2 |

[a] Percentage of not viable cells @ concentration compared to cells treated with DMSO: T = mild toxicity (5-25%); TT = medium toxicity (25-50%); TTT = severe toxicity (>50%).

### Library II

### DENV2 NS2B-NS3 biochemical inhibition

**48**

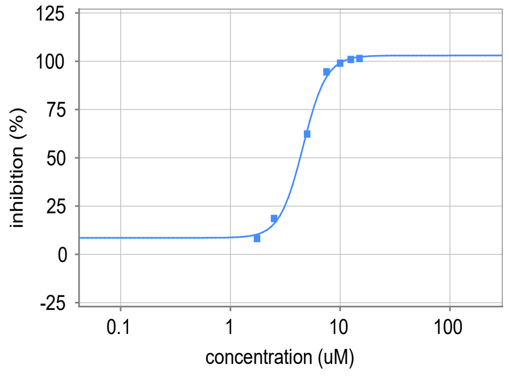


**49**

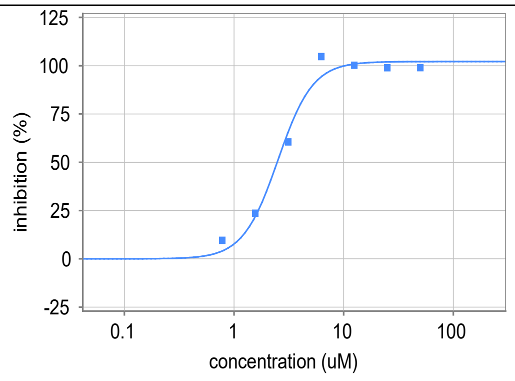


**55**

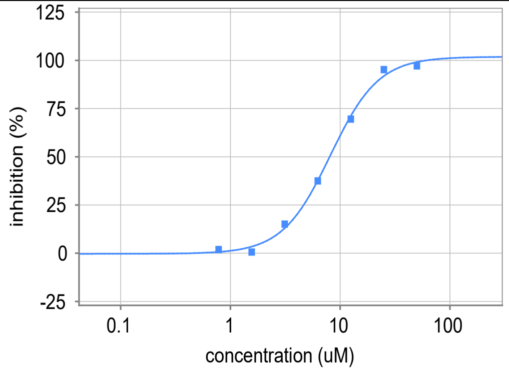


**50**

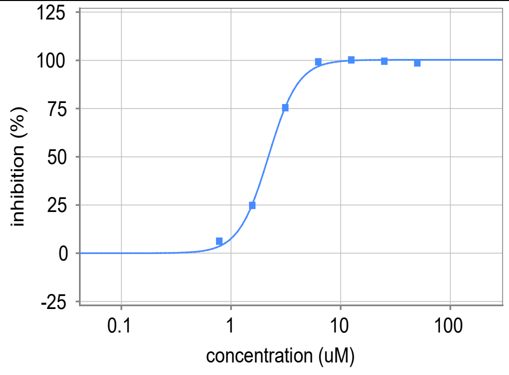


**51**

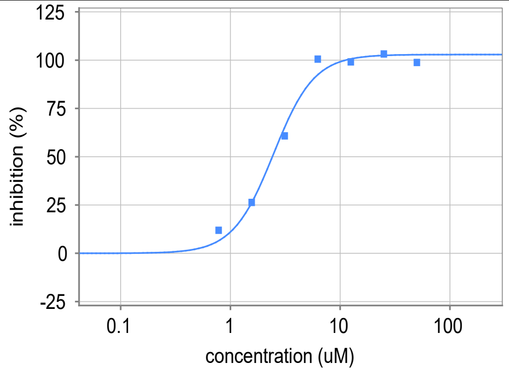


**52**

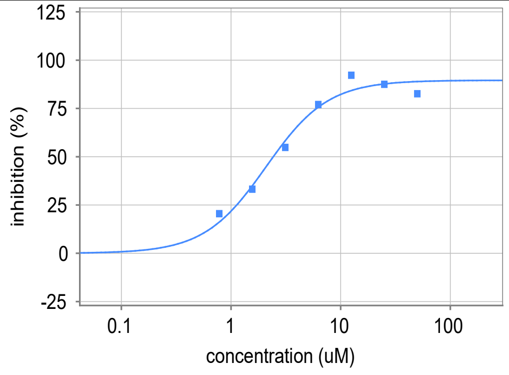

**53**

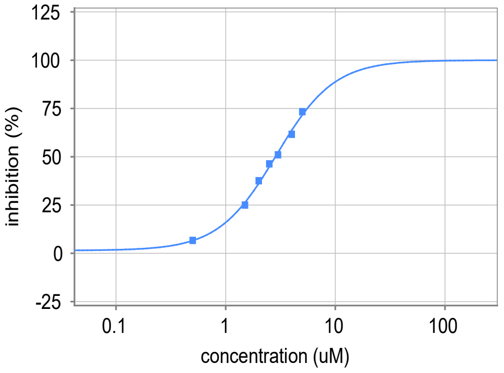


**56**

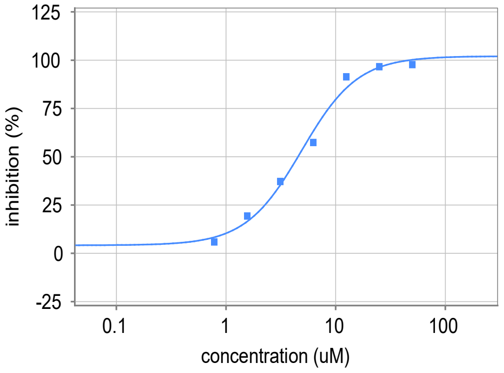


**57**

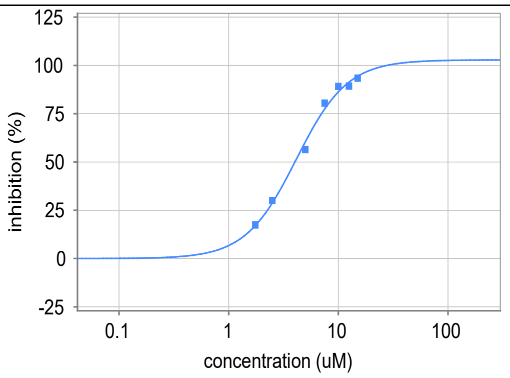


**58**

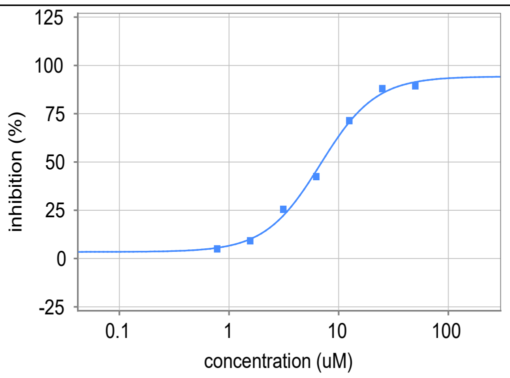


**60**

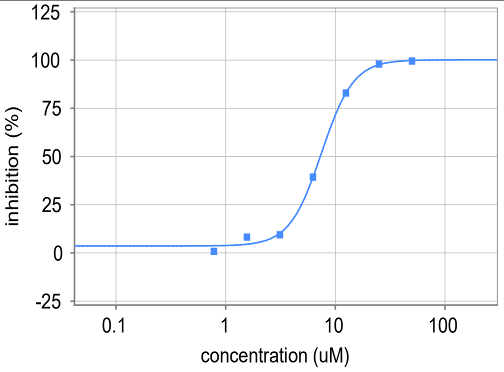


**61**

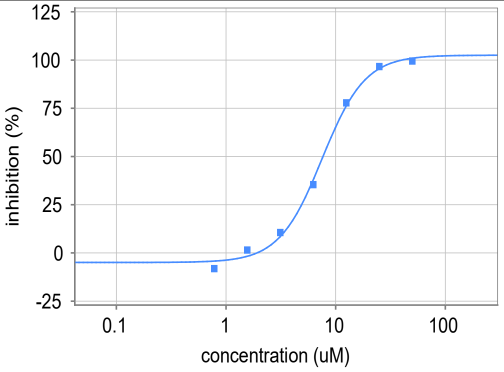


**62**

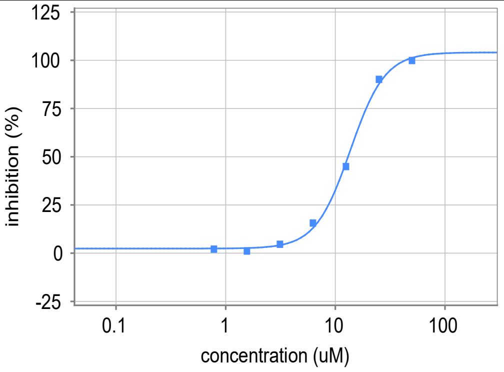


**63**

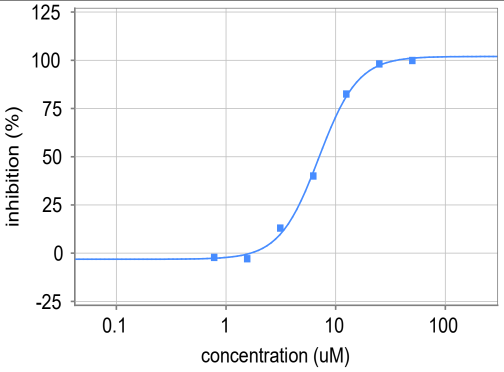

**64**

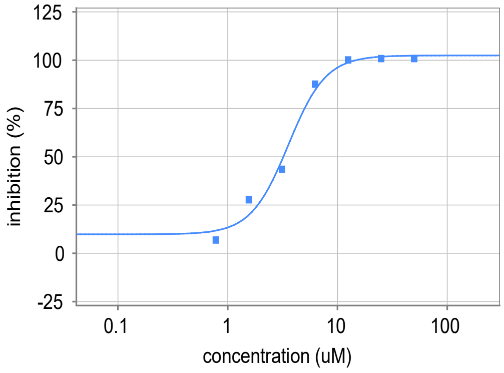


**65**

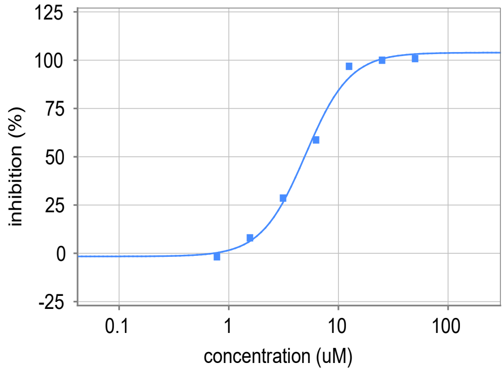


**66**

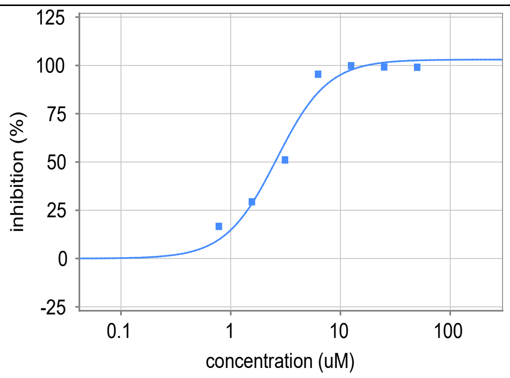


**67**

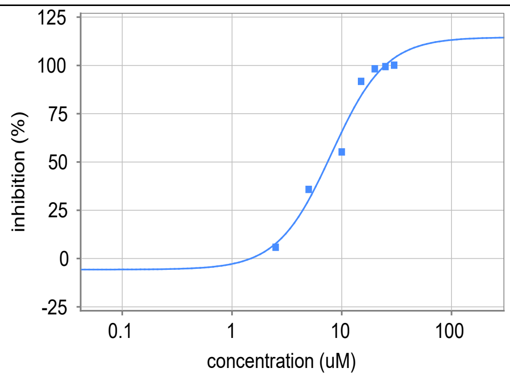


**68**

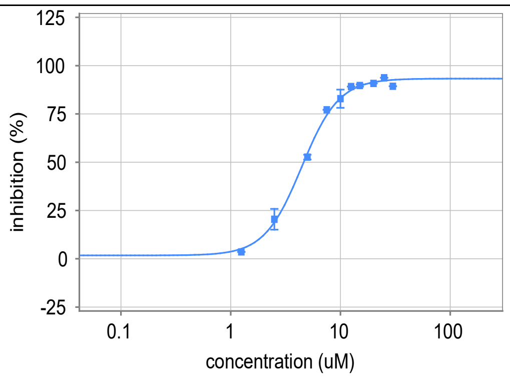


**69**

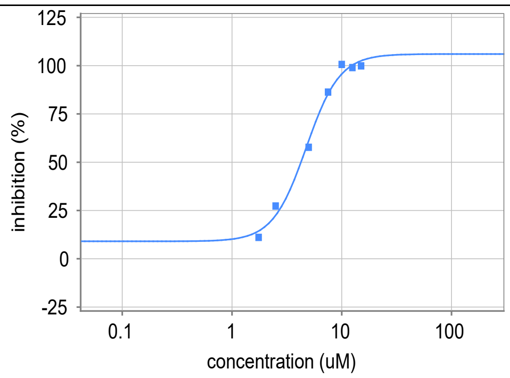


**70**

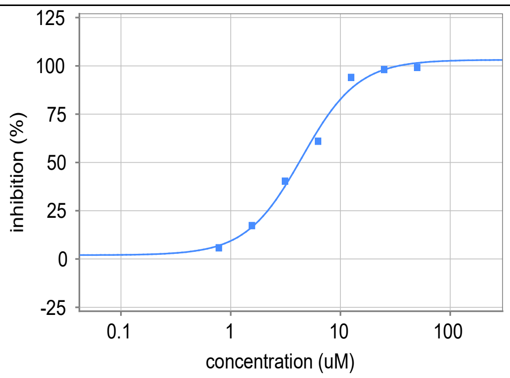

71

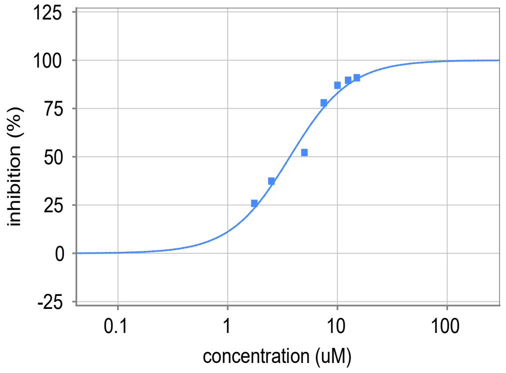


72

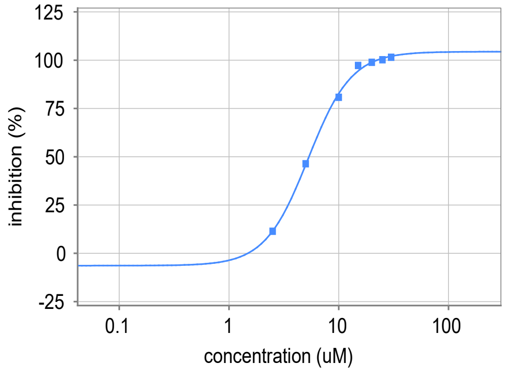


73

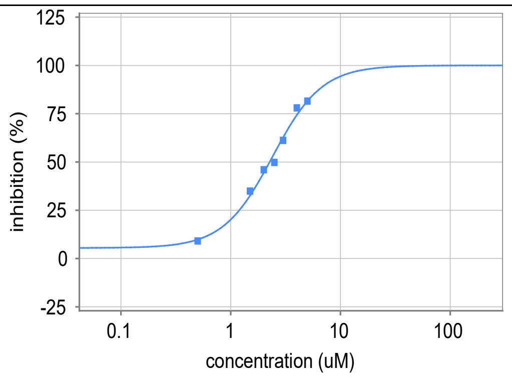


74

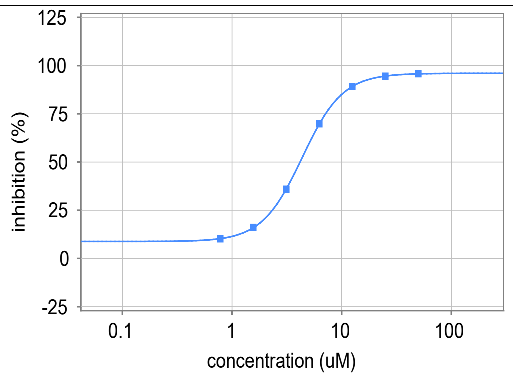


75

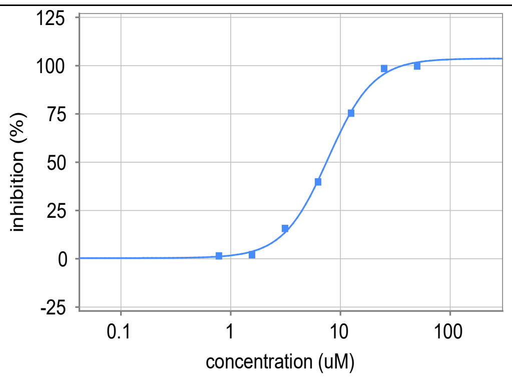


76

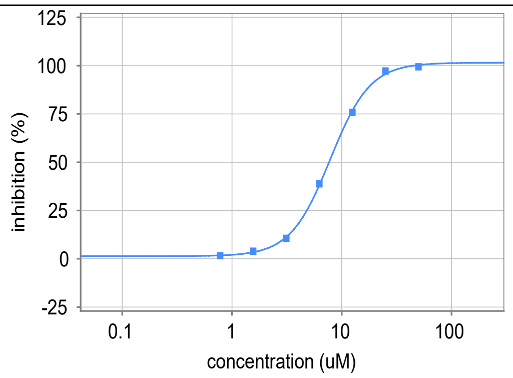


77

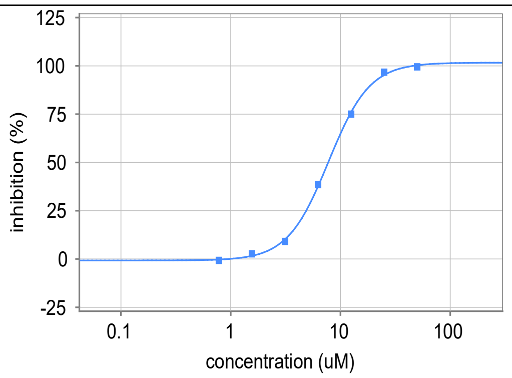


78

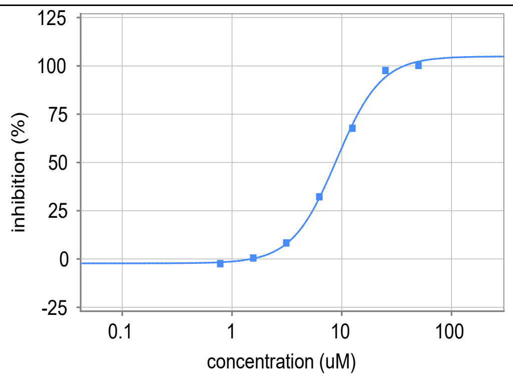

| | | |
|---|---|---|
| **79** | 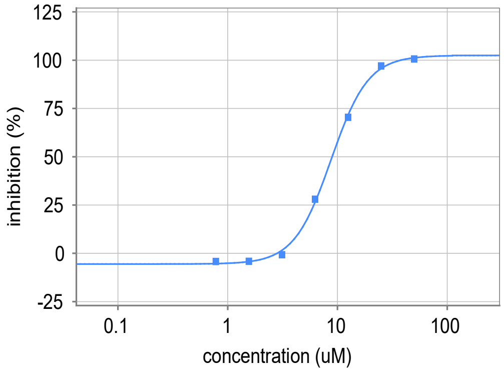 | |

## DENV2 cellular infection

| | | | |
|---|---|---|---|
| **40** | Protocol 1, LLC-MK cells | **41** | Protocol 1, LLC-MK cells |
| 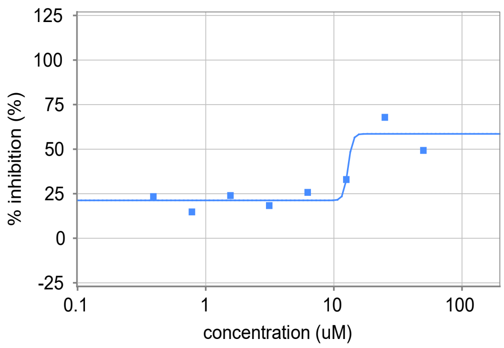 | | 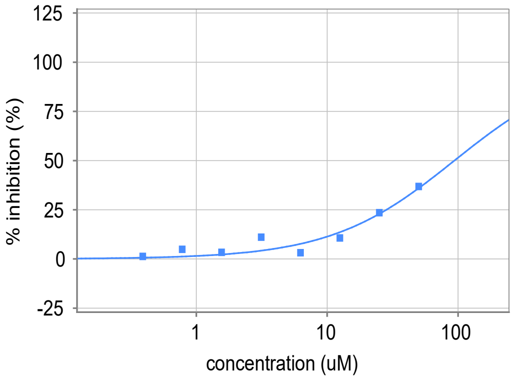 | |
| **42** | Protocol 1, Vero cells | **43** | Protocol 1, LLC-MK cells |
| 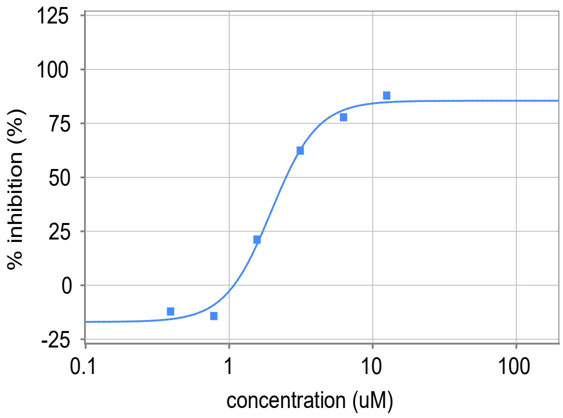 | | 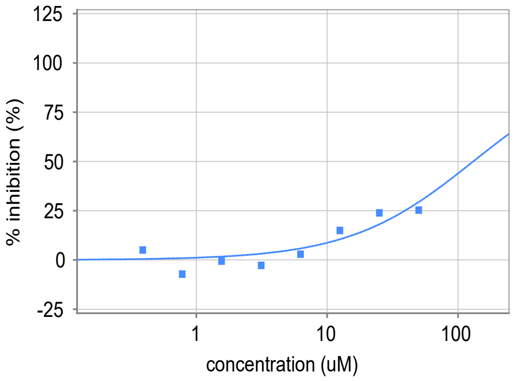 | |
| **44** | Protocol 1, Vero cells | **45** | Protocol 1, LLC-MK cells |
| 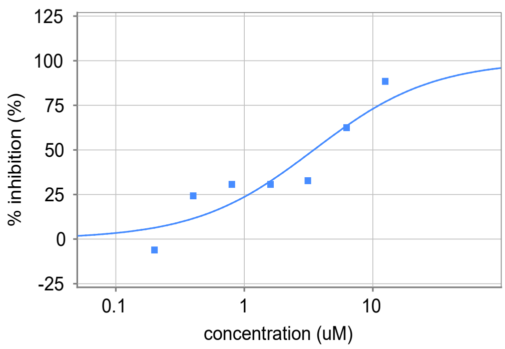 | | 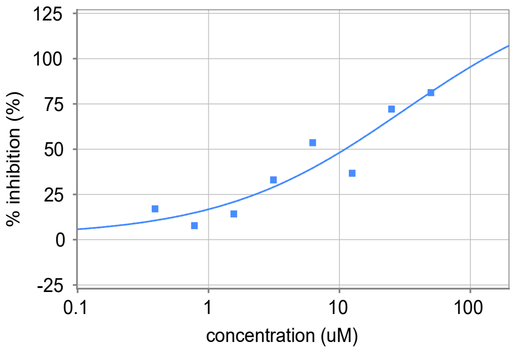 | |
| | | | |

| 46 | Protocol 1, LLC-MK cells | 47 | Protocol 1, LLC-MK cells |
|---|---|---|---|
| 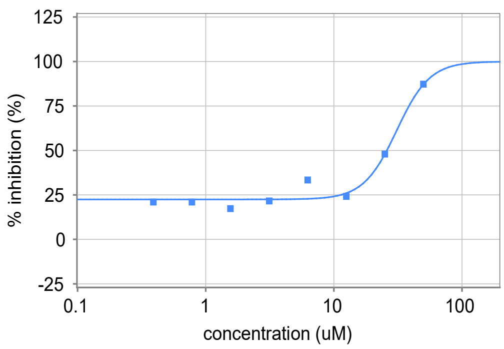 | | 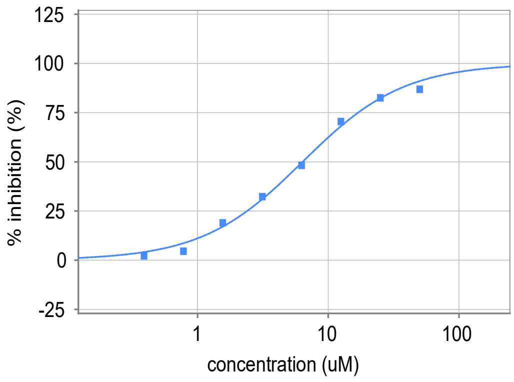 | |
| **48** | Protocol 2, BHK cells | **49** | Protocol 1, Vero cells |
| 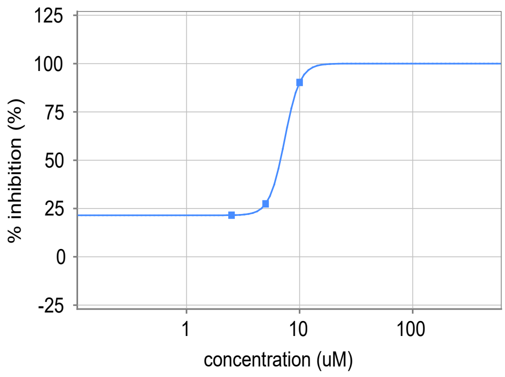 | | 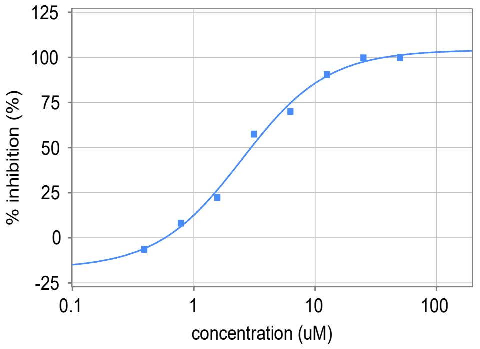 | |
| **50** | Protocol 1, LLC-MK cells | **51** | Protocol 1, Vero cells |
| 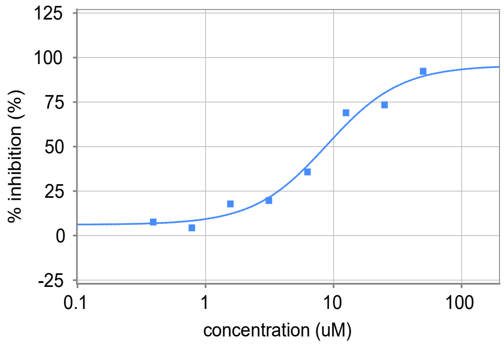 | | 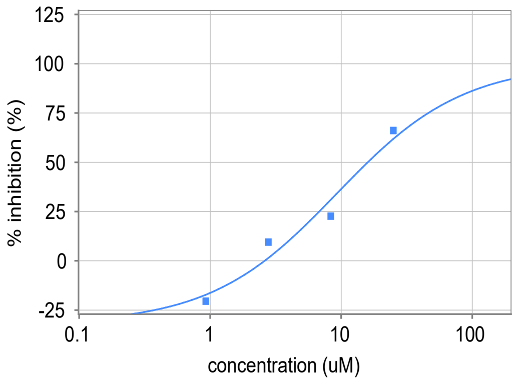 | |
| **52** | Protocol 2, BHK cells | **53** | Protocol 1, LLC-MK cells |
| 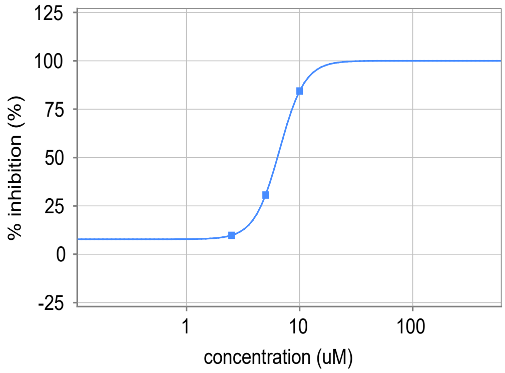 | | 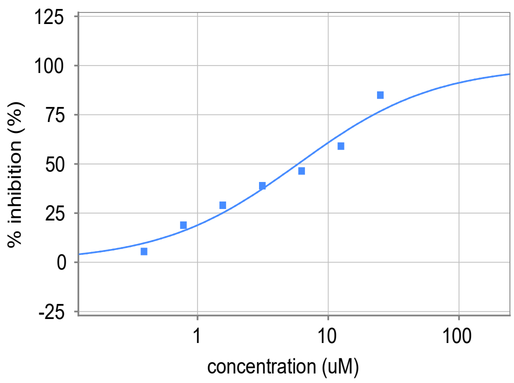 | |
| **55** | Protocol 2, BHK cells | **56** | Protocol 2, BHK cells |

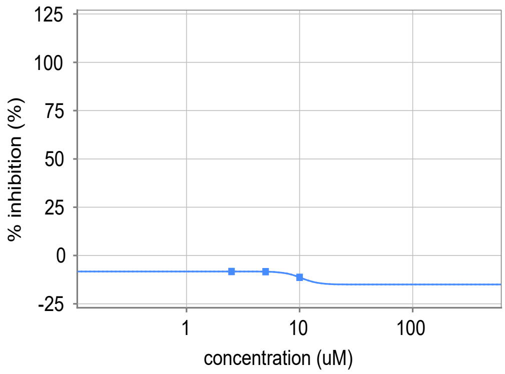


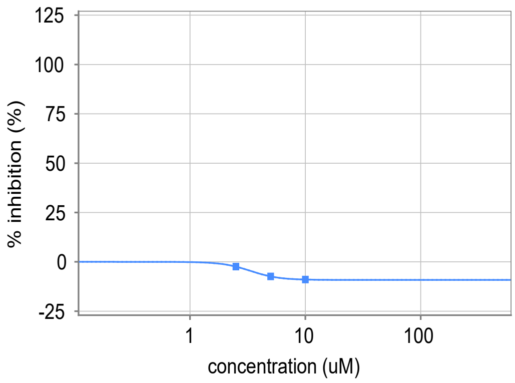


**57** Protocol 2, BHK cells

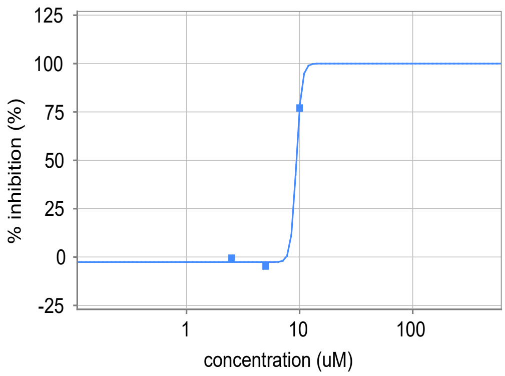


**58** Protocol 2, BHK cells

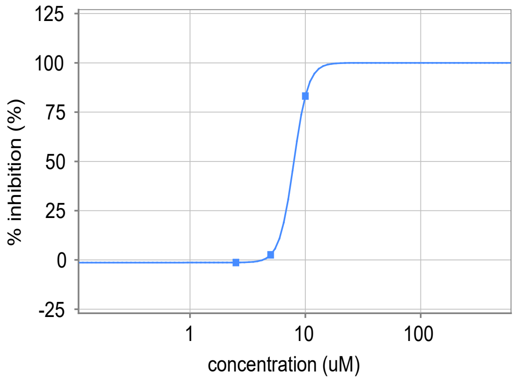


**60** Protocol 2, BHK cells

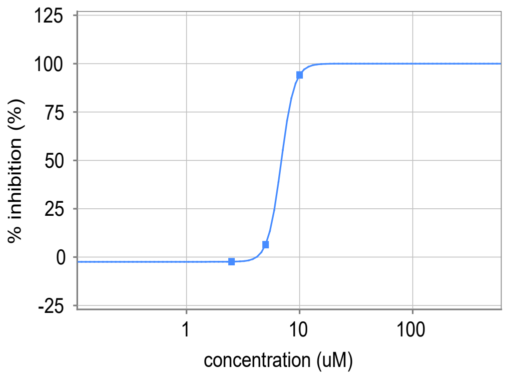


**61** Protocol 2, BHK cells

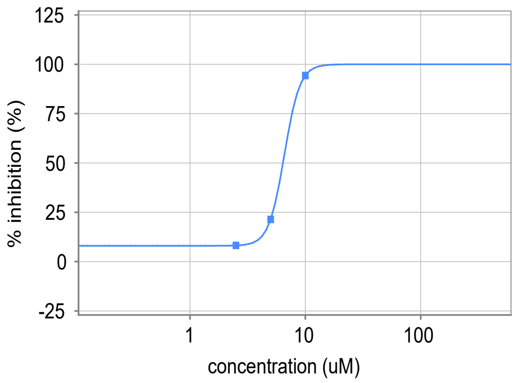

**63** Protocol 2, BHK cells

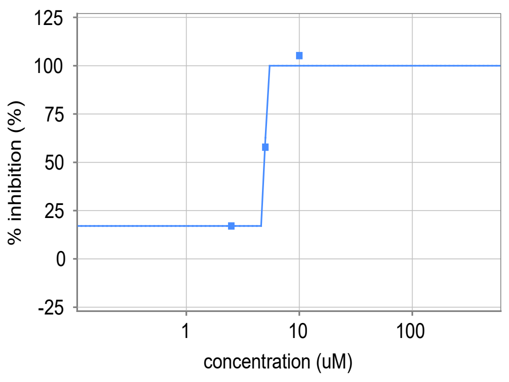


**64** Protocol 1, Vero cells

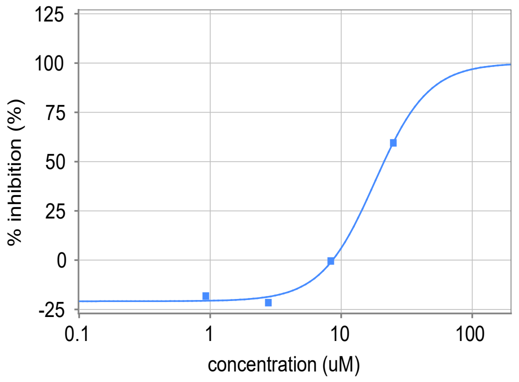


**65** Protocol 1, Vero cells

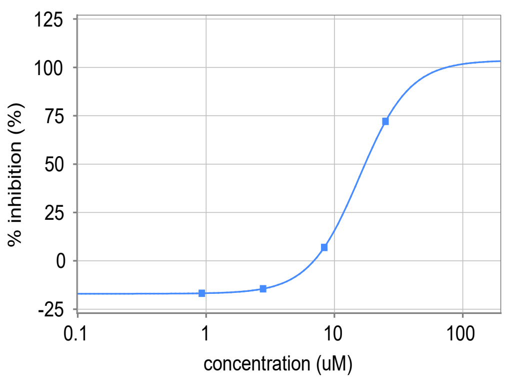


**66** Protocol 1, Vero cells

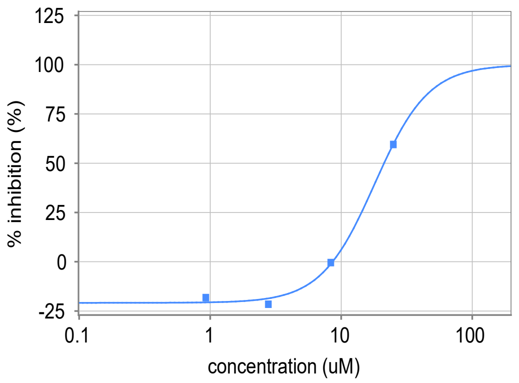


**67** Protocol 1, LLC-MK cells

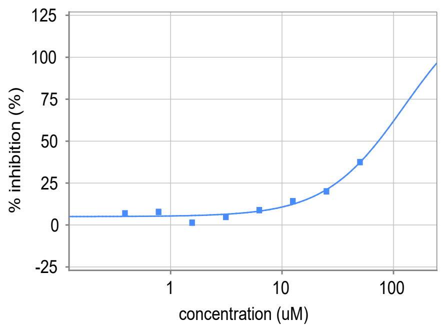


**68** Protocol 1, LLC-MK cells

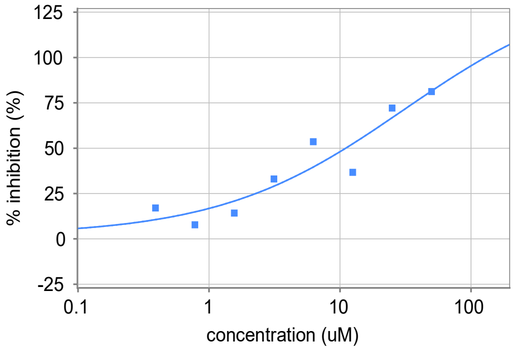


**69** Protocol 1, LLC-MK cells

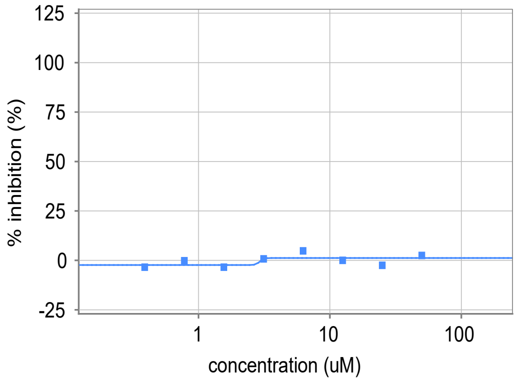


**70** Protocol 2, BHK cells

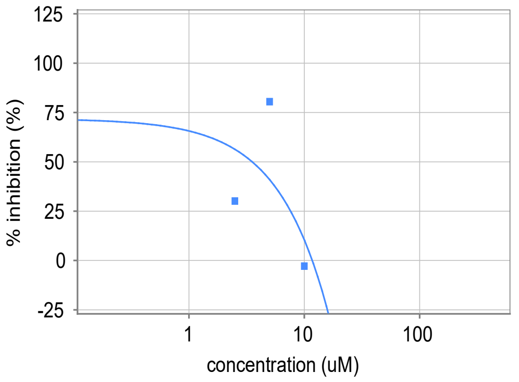

**71** Protocol 1, LLC-MK cells

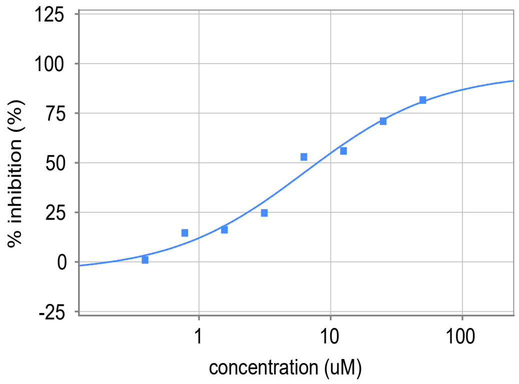


**72** Protocol 1, LLC-MK cells

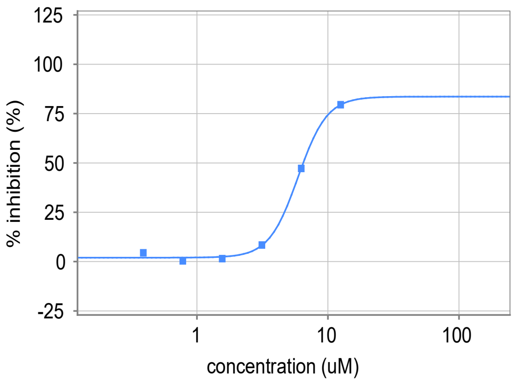


**73** Protocol 1, LLC-MK cells

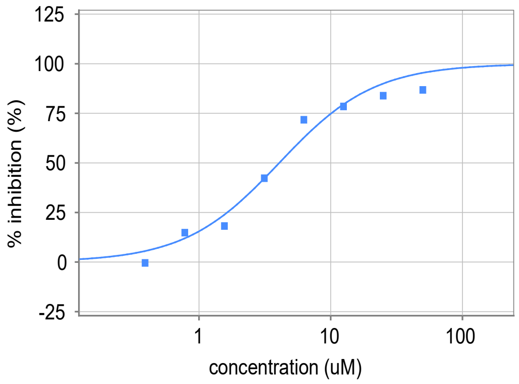


**74** Protocol 2, BHK cells

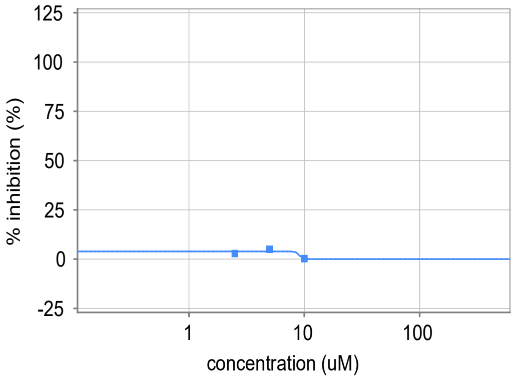


**75** Protocol 1, Vero cells

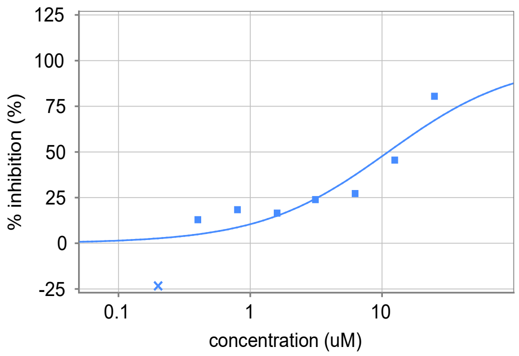


**76** Protocol 2, BHK cells

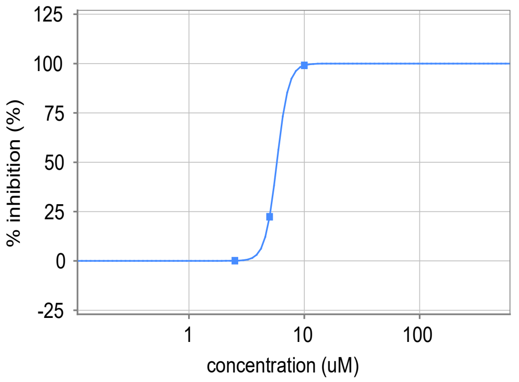


**77** Protocol 2, BHK cells

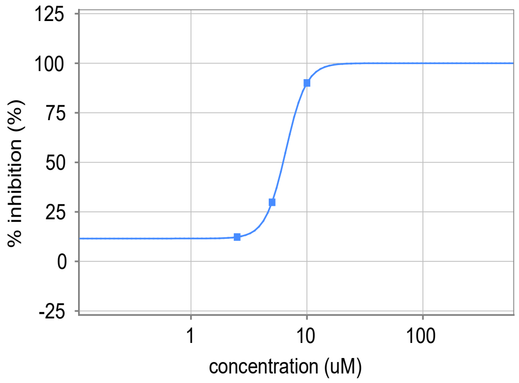


**78** Protocol 2, BHK cells

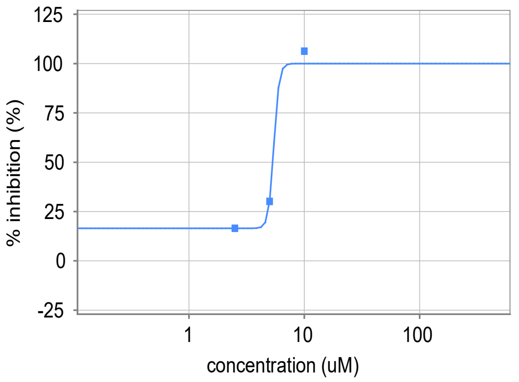

**79** Protocol 2, BHK cells

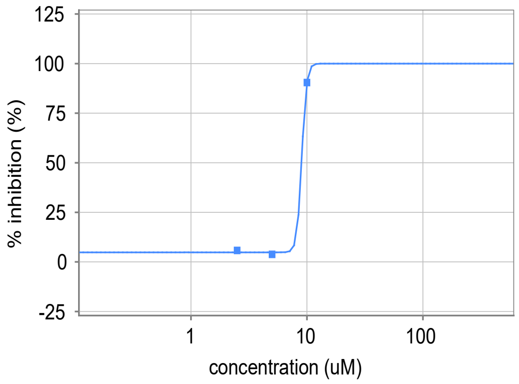


## Viability

**42** Protocol 1, Vero cells

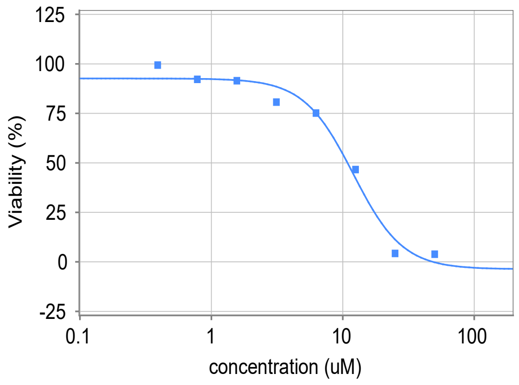


**44** Protocol 1, Vero cells

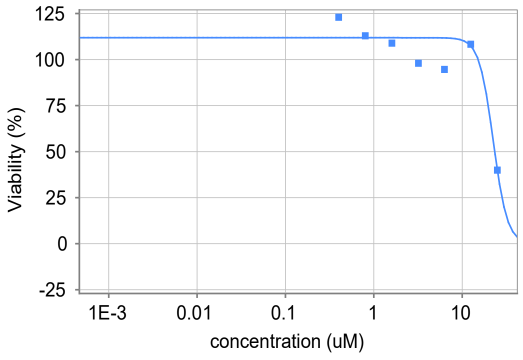


**Table S 2. Observed cellular toxicity for compounds in Library II per concentration point.[a]**

| Compound | Concentration (µM) | Tox | cells |
|---|---|---|---|
| **48** | 2.5 | T | BHK |
| | 5 | T | BHK |
| | 10 | T | BHK |
| **55** | 2.5 | T | BHK |
| | 5 | T | BHK |
| | 10 | T | BHK |
| **56** | 5 | T | BHK |
| | 10 | TT | BHK |
| **57** | 5 | T | BHK |
| | 10 | TT | BHK |
| **58** | 2.5 | T | BHK |
| | 5 | T | BHK |
| | 10 | TT | BHK |
| **60** | 2.5 | T | BHK |
| | 5 | T | BHK |
| | 10 | T | BHK |

| | | | |
|---|---|---|---|
| **61** | 5 | T | BHK |
| **63** | 2.5 | T | BHK |
| | 5 | T | BHK |
| **70** | 2.5 | T | BHK |
| | 5 | TT | BHK |
| | 10 | TTT | BHK |
| **72** | 50 | TTT | LLC-MK2 |
| | 25 | TTT | LLC-MK2 |
| | 12.5 | T | LLC-MK2 |
| **74** | 2.5 | T | BHK |
| | 5 | T | BHK |
| | 10 | TT | BHK |
| **77** | 10 | T | BHK |

[a] Percentage of not viable cells @ concentration compared to cells treated with DMSO: T = mild toxicity (5-25%); TT = medium toxicity (25-50%); TTT = severe toxicity (>50%).

**WNV and ZIKV NS2B-N3 inhibition**

| | WNV | ZIKV |
|---|---|---|
| **73** | 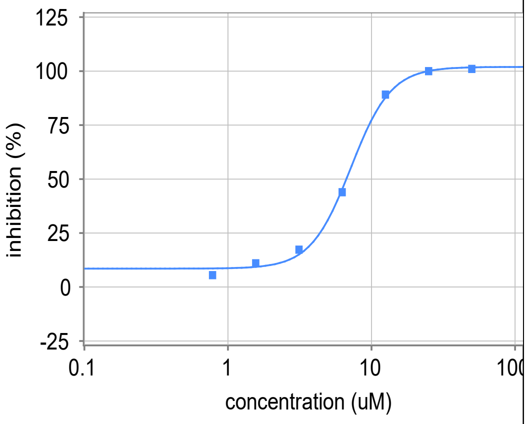 | 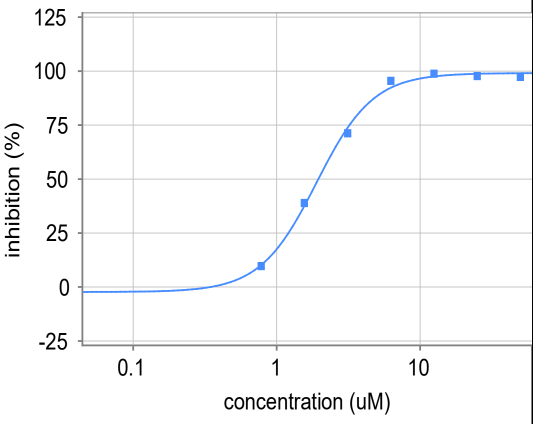 |
| **76** | 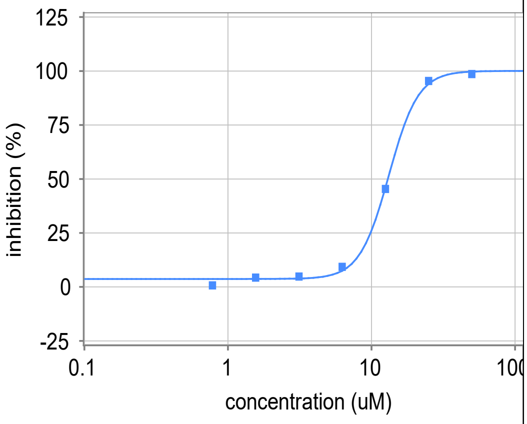 | 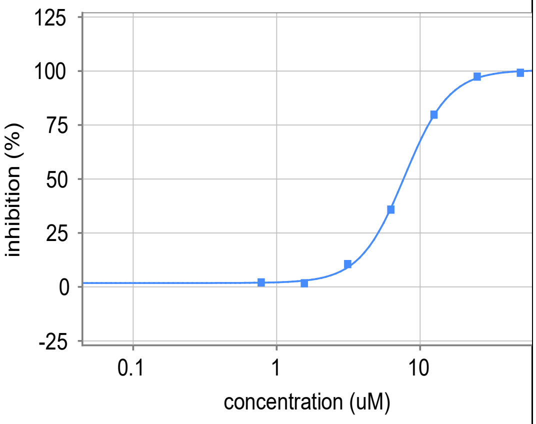 |
| **79** | 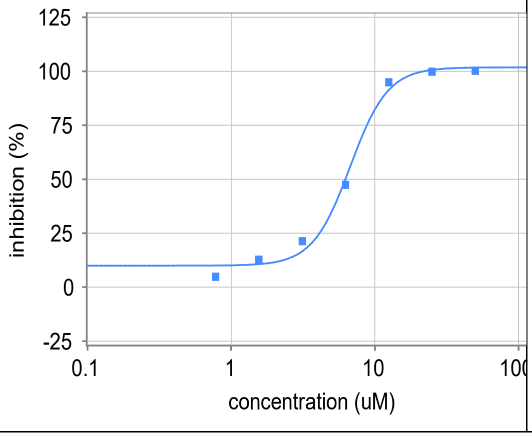 | 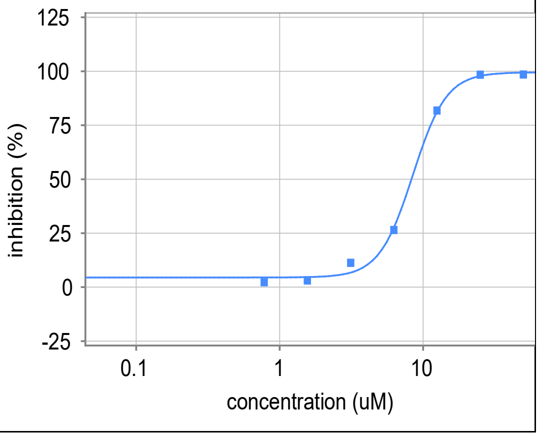 |

**WNV and ZIKV cellular viral infection data**

| | WNV | ZIKV |
|---|---|---|
| **73** | 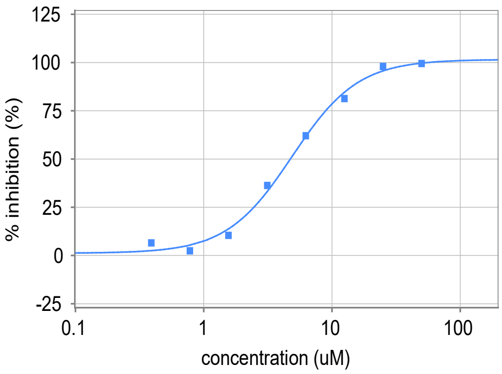 | 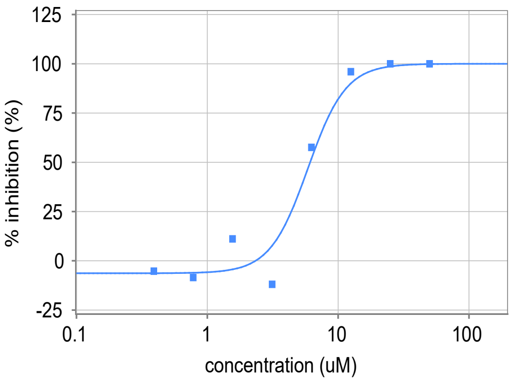 |
| **79** | 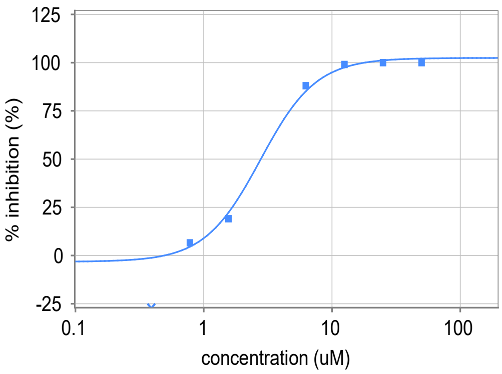 | 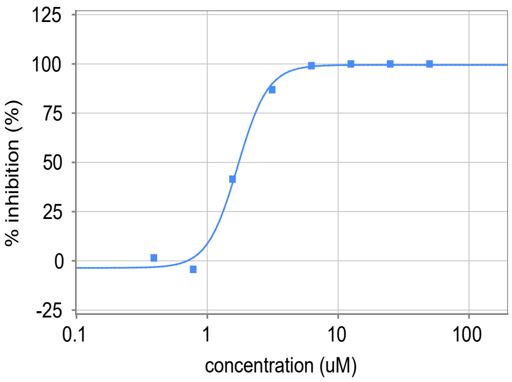 |

Protocol 3, Vero Cells

## Spectral Material

### LCMS traces

**Figure S 3. Chromatogram of 73 (RT = 13,54 min, purity 96,47%) and detected peaks, recorded at 210 nM:**

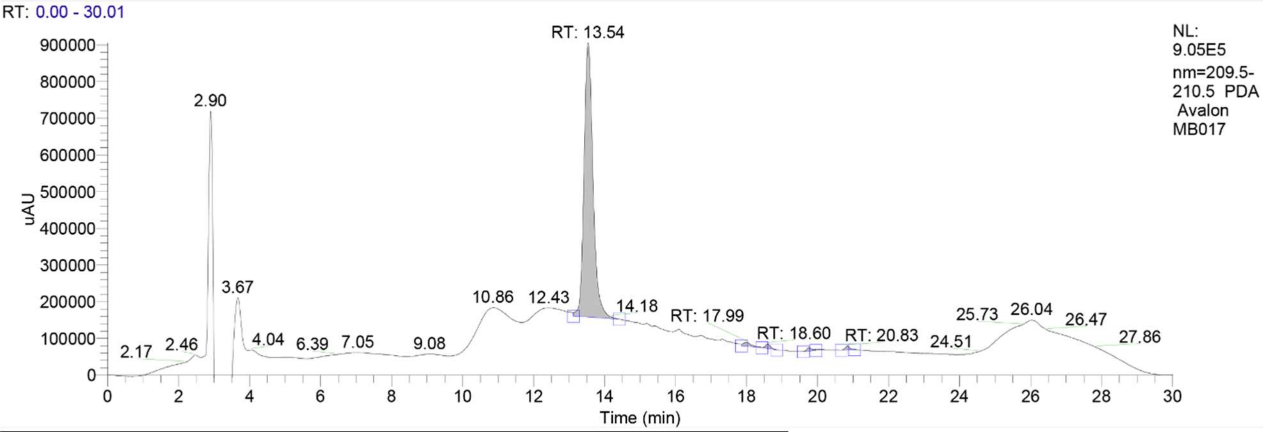


| RT: 0.00 - 30.01<br>Number of detected peaks: 5 | | | | | | |
|---|---|---|---|---|---|---|
| Apex RT | Start RT | End RT | Area | %Area | Height | %Height |
| 13,54 | 13,12 | 14,41 | 13296350 | 96,47 | 746967,3 | 94,37 |
| 17,99 | 17,86 | 18,43 | 163346,6 | 1,19 | 11093,13 | 1,4 |
| 18,6 | 18,43 | 18,85 | 119870,7 | 0,87 | 13013,87 | 1,64 |
| 19,75 | 19,59 | 19,95 | 101311,4 | 0,74 | 9442 | 1,19 |
| 20,83 | 20,67 | 21,03 | 101611,2 | 0,74 | 10998,65 | 1,39 |

**Figure S 4. Chromatogram of 79 (RT = 12.92 min, purity 98.03%.) and detected peaks, recorded at 210 nM:**

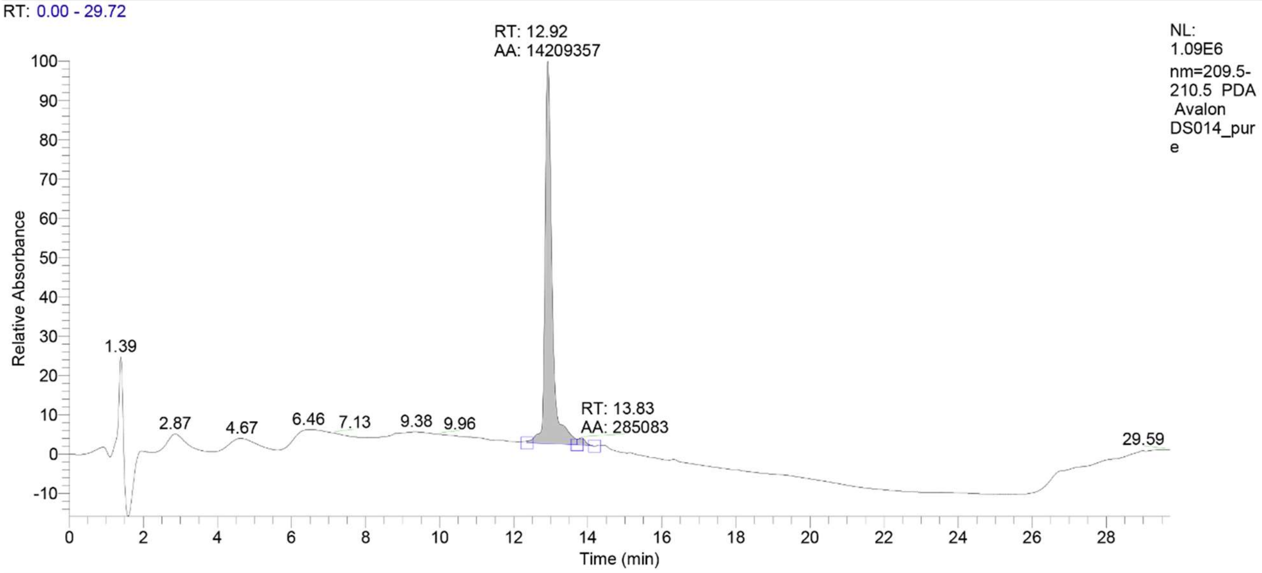


| RT: 0.00 - 29.72<br>Number of detected peaks: 2 | | | | | | |
|---|---|---|---|---|---|---|
| Apex RT | Start RT | End RT | Area | %Area | Height | %Height |
| 12,92 | 12,35 | 13,71 | 14209357 | 98,03 | 1058581 | 98,1 |
| 13,83 | 13,71 | 14,18 | 285083 | 1,97 | 20514,88 | 1,9 |

**HPLC**

**Figure S 5. HPLC trace of compound 73.**

| | | | |
|---|---|---|---|
| Injection Volume: | 35.00 ul | Date Acquired: | 17-Sep-2021 03:20:10 PM IST |
| Run Time: | 20.0 Minutes | Proc. Chnl. Descr. | 2998 PDA 210.0 nm (2998 (210-400)nm) |
| Acq. Method Set: | BPT143_1 | | |

HPLC Method Conditions :

Column : X-Bridge Peptide C18 300A (4.6X250mm) 5µm
Mobile Phase-A : 0.1% TFA in Water
Mobile Phase-B : 0.1% TFA in Acetonitrile
Gradient (T/% B) : 0/30, 14/95, 20/95, 20.1/30
Flow Rate : 1.0 mL/min
Temparature : 60℃
Diluent : Acn:Water(1:1)

Zoom Scaled Chromatogram

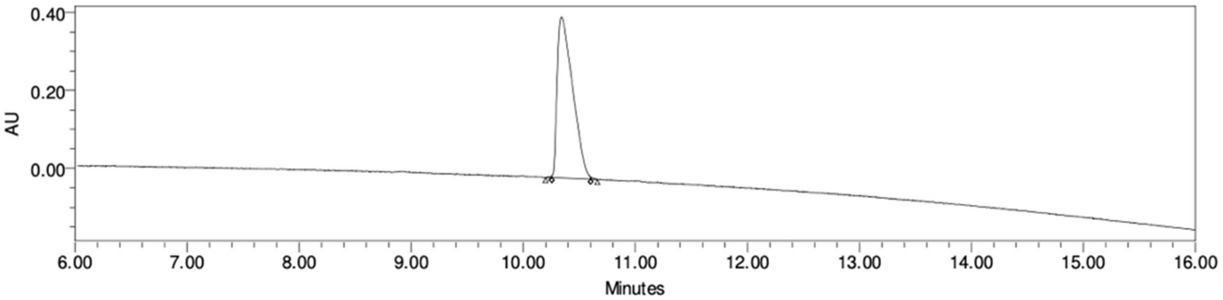


Auto-Scaled Chromatogram

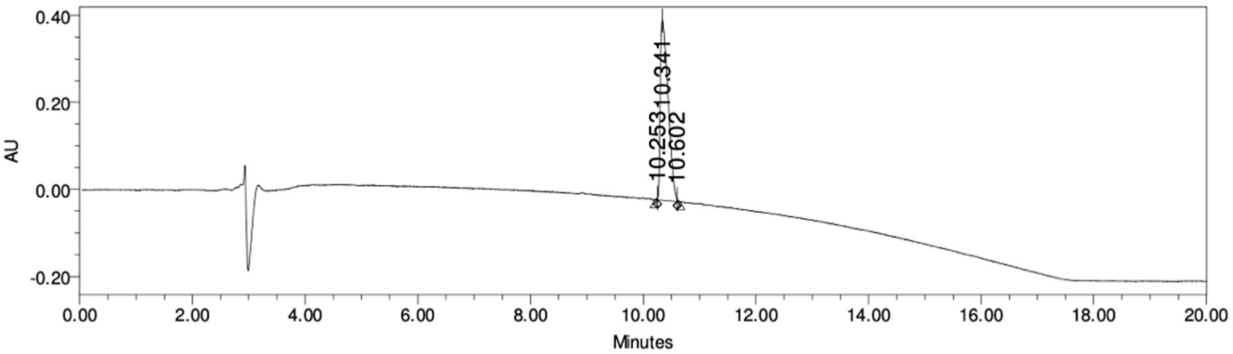


**Peak Results**

| | RT | Area | Height | % Area |
|---|---|---|---|---|
| 1 | 10.253 | 6126 | 3936 | 0.15 |
| 2 | 10.341 | 3965110 | 413905 | 99.66 |
| 3 | 10.602 | 7204 | 5415 | 0.18 |

**NMR spectra**

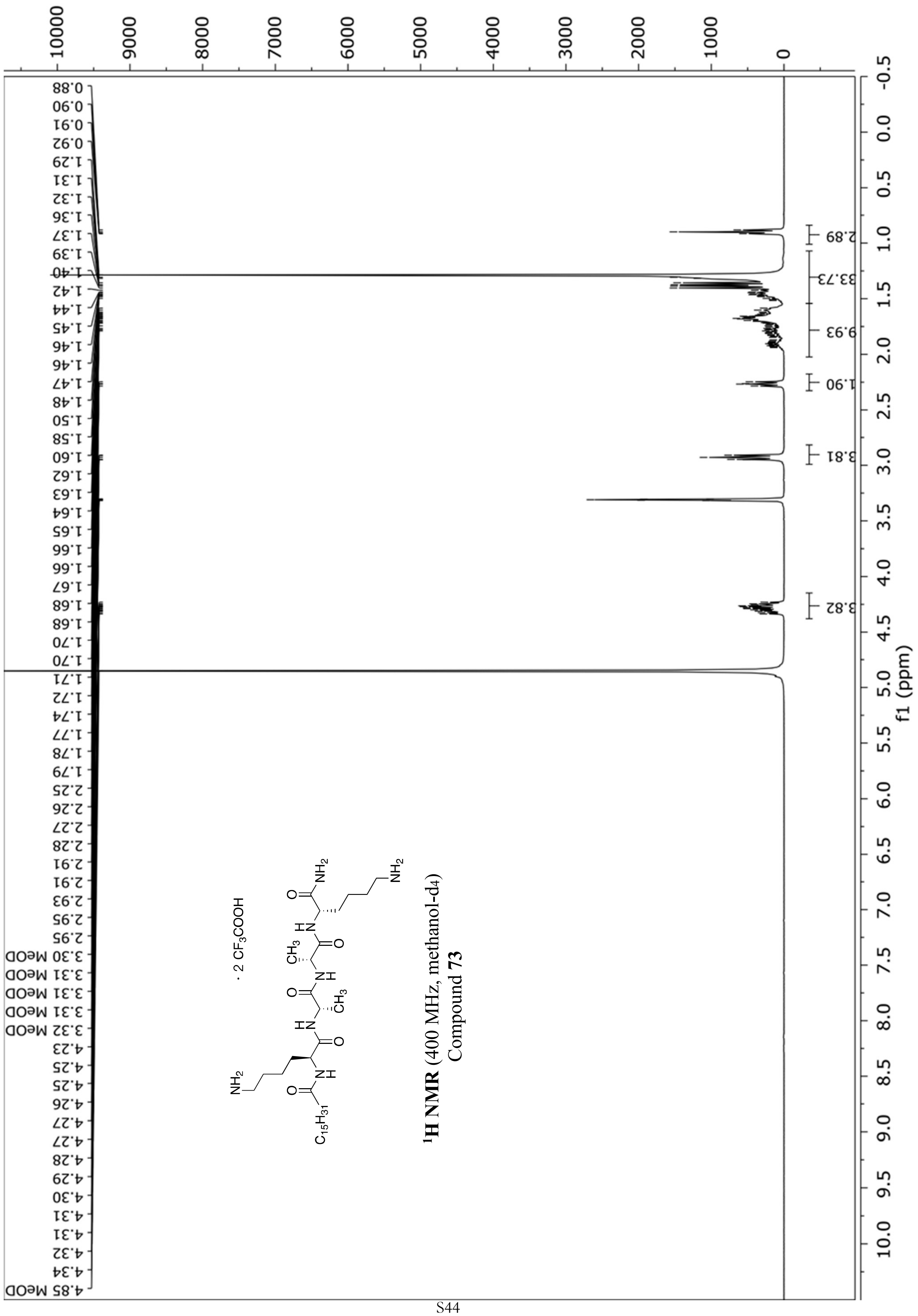


$^1$**H NMR** (400 MHz, methanol-d4)
Compound **73**

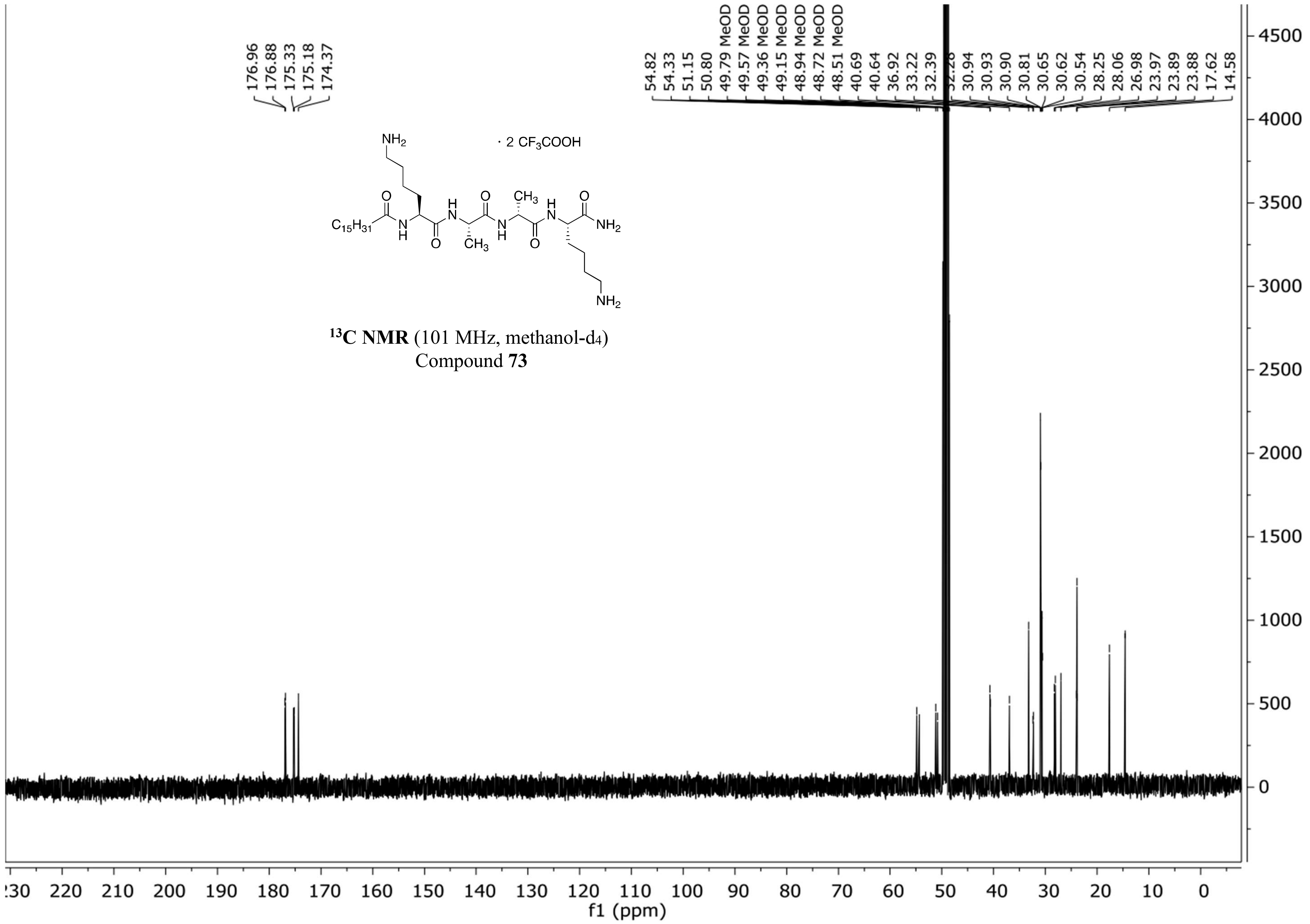


**$^{13}C$ NMR** (101 MHz, methanol-d4)
Compound **73**

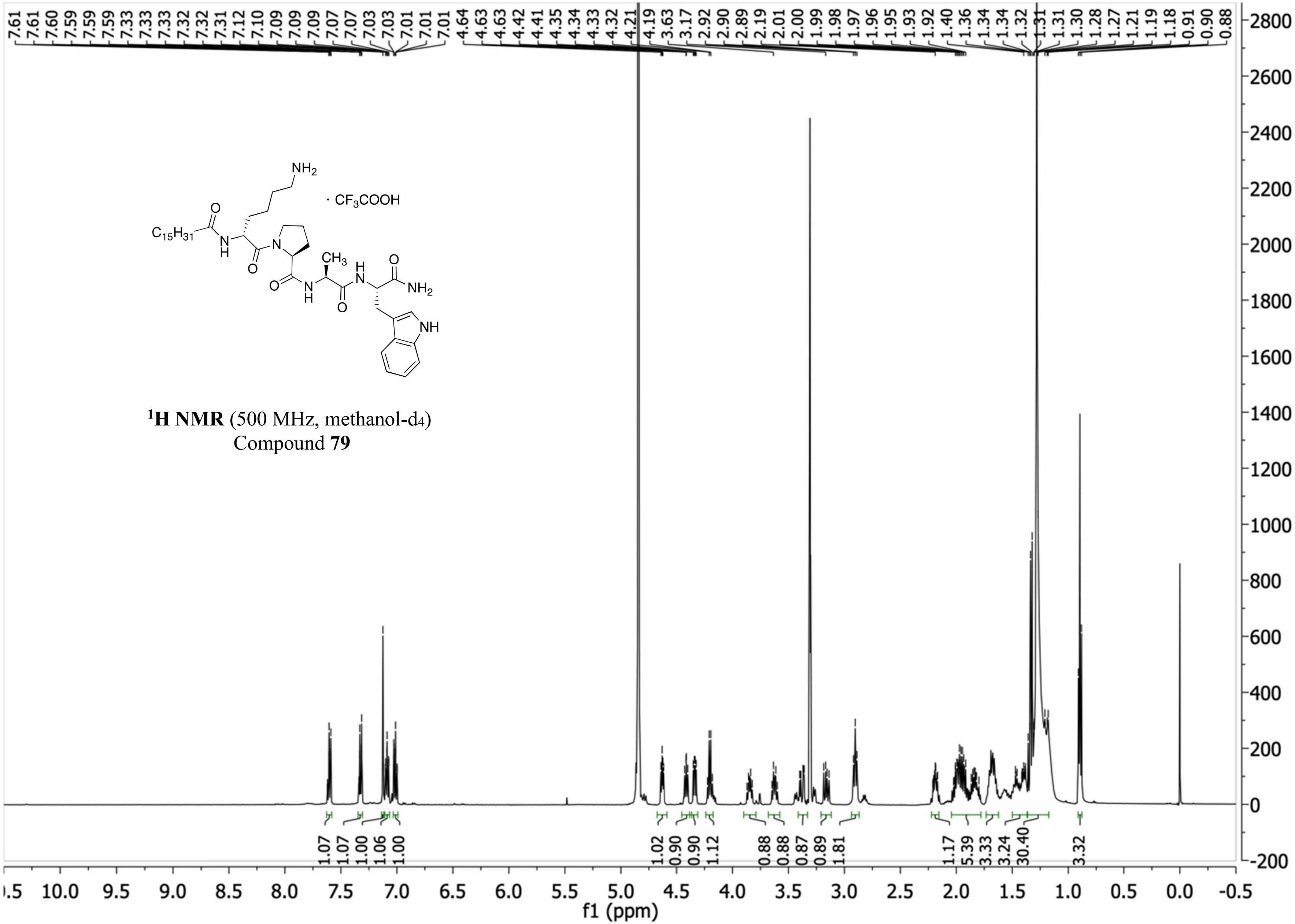


**$^1$H NMR** (500 MHz, methanol-d4)
Compound **79**

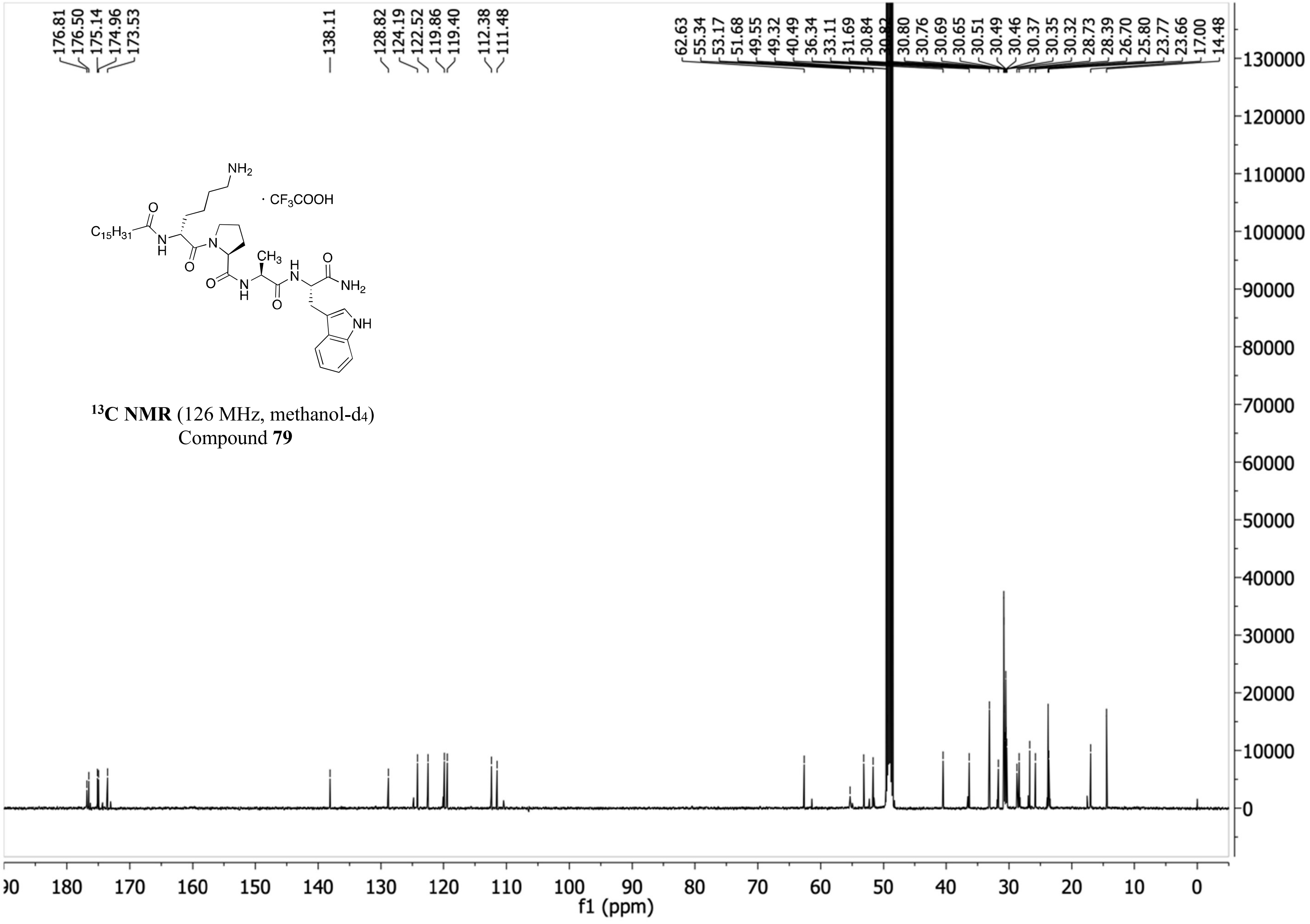


**¹³C NMR** (126 MHz, methanol-d4)
Compound **79**

# AAALAC accreditation certificate

aaalac INTERNATIONAL

[illegible]
[illegible]

June 16, 2025

Kumaran Dandapani, M. Pharm, Ph.D.
Director
Animal Pharmacology, Biology Solutions
Aragen Life Sciences Pvt Ltd
28A, Road Number 15
Industrial Development Area (IDA)
Nacharam, Hyderabad, Telangana, 500076
India

Dear Dr. Dandapani:

The AAALAC International Council on Accreditation has reviewed the report of the recent site visit to Aragen Life Sciences Private Limited, Nacharam, Hyderabad, Telangana, India. The Council commends you and the staff for providing and maintaining a high quality program of laboratory animal care and use. Especially noteworthy were the well coordinated and strong administrative support and commitment to maintaining an animal care and use program; the excellent online incident reporting system; and the in-house medical doctor arrangement. The Council is pleased to inform you that the program conforms with AAALAC International standards as set forth by the *Guide for the Care and Use of Laboratory Animals*, NRC 2011. Therefore, **FULL ACCREDITATION** shall continue.

Council acknowledges receipt of the correspondence dated March 28, 2025 detailing actions taken relative to concerns expressed by the site visitors during the exit briefing. Specifically, the items addressed satisfactorily included: incorporating allergy evaluation questions into the personal medical evaluation form; replacing the plantar aesthesiometer bottom plates and stands with new ones; revising the practice for storing the sterilized corncob and labeling its containers; repairing or replacing the autoclave water drainpipes to a proper condition; monitoring sanitation effectiveness of walls, ceiling, and floor; developing the adverse event assessment and reporting plan; replacing wooden pallets with plastic ones; and correcting the Program Description inaccuracy. Council has no further recommendations to offer for improvement of the animal care and use program at this time. We look forward to following your program activities and wish you continued success.

AAALAC International requires an Annual Report detailing changes made during the year in accredited units. In the interim, AAALAC International expects to be apprised in a timely manner of significant programmatic changes or adverse events should they occur. Please note that, at your request, AAALAC International will provide your institution with a separate letter simply verifying that your animal care and use program is accredited.

Sincerely,

Dewi K. Rowlands, M.Phil., M.B.A., Ph.D.
President, Council on Accreditation

DKR:mem
001343

cc: Shanavas Alikunju, M.S., Ph.D, Senior Vice President and Institutional Official
Sandeep Kumar Pandey, B.V.Sc., Associate Scientist and Attending Veterinarian

[illegible]

European Office
[illegible], Spain
[illegible]

North American Office
[illegible] Court, Suite 300
[illegible] USA [illegible]
[illegible]

# References

1. Nitsche, C.; Klein, C. D., Fluorimetric and HPLC-Based Dengue Virus Protease Assays Using a FRET Substrate. In *Antiviral Methods and Protocols*, Gong, E. Y., Ed. Humana Press: Totowa, New Jersey, 2013; pp 221-236.

2. Nitsche, C.; Schreier, V. N.; Behnam, M. A. M.; Kumar, A.; Bartenschlager, R.; Klein, C. D., Thiazolidinone–Peptide Hybrids as Dengue Virus Protease Inhibitors with Antiviral Activity in Cell Culture. *J. Med. Chem.* **2013,** *56* (21), 8389-8403.

3. Kühl, N.; Graf, D.; Bock, J.; Behnam, M. A. M.; Leuthold, M.-M.; Klein, C. D., A New Class of Dengue and West Nile Virus Protease Inhibitors with Submicromolar Activity in Reporter Gene DENV-2 Protease and Viral Replication Assays. *J. Med. Chem.* **2020,** *63* (15), 8179-8197.

4. Nitsche, C.; Passioura, T.; Varava, P.; Mahawaththa, M. C.; Leuthold, M. M.; Klein, C. D.; Suga, H.; Otting, G., De Novo Discovery of Nonstandard Macrocyclic Peptides as Noncompetitive Inhibitors of the Zika Virus NS2B-NS3 Protease. *ACS Med. Chem. Lett.* **2019,** *10* (2), 168-174.

5. Weigel, L. F.; Nitsche, C.; Graf, D.; Bartenschlager, R.; Klein, C. D., Phenylalanine and Phenylglycine Analogues as Arginine Mimetics in Dengue Protease Inhibitors. *J. Med. Chem.* **2015,** *58* (19), 7719-7733.

6. Spektor, A. Setting up a Dose Response Protocol. https://support.collaborativedrug.com/hc/en-us/articles/214359303-Setting-up-a-Dose-Response-Protocol (accessed 29 September).
